# Antibacterial and ATP Synthesis Modulating Compounds from *Salvia tingitana*

**DOI:** 10.1021/acs.jnatprod.9b01024

**Published:** 2020-03-17

**Authors:** Angela Bisio, Anna M. Schito, Francesca Pedrelli, Ombeline Danton, Jakob K. Reinhardt, Giulio Poli, Tiziano Tuccinardi, Thomas Bürgi, Francesco De Riccardis, Mauro Giacomini, Daniela Calzia, Isabella Panfoli, Gian Carlo Schito, Matthias Hamburger, Nunziatina De Tommasi

**Affiliations:** † Department of Pharmacy, University of Genova, Viale Cembrano 4, 16148 Genova, Italy; ‡ Department of Integrated Surgical and Diagnostical Sciences, University of Genova, Largo Rosanna Benzi 8, 16145 Genova, Italy; § Department of Pharmaceutical Sciences, University of Basel, Klingelbergstrasse 50, 4056 Basel, Switzerland; ⊥ Department of Pharmacy, University of Pisa, Via Bonanno 6, 56126 Pisa, Italy; ∥ Department of Chemical Physics, University of Geneva, 30 Quai Ernest-Ansermet, 1211 Genève 4, Switzerland; ∇ Department of Chemistry and Biology, University of Salerno, Via Giovanni Paolo II 132, 84084 Salerno, Italy; ○ Department of Informatics Bioengineering Robotics and System Engineering, University of Genova, Via all’Opera Pia, 13, 16145 Genova, Italy; □ Department of Pharmacy, University of Salerno, Via Giovanni Paolo II 132, 84084 Salerno, Italy

## Abstract

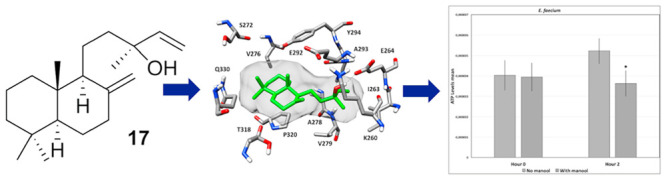

A surface extract of the aerial parts of *Salvia tingitana* afforded a nor-sesterterpenoid (**1**) and eight new sesterterpenoids (**2**–-**9**), along with five known sesterterpenoids, five labdane and one abietane diterpenoid, one sesquiterpenoid, and four flavonoids. The structures of the new compounds were established by 1D and 2D NMR spectroscopy, HRESIMS, and VCD data and Mosher’s esters analysis. The antimicrobial activity of compounds was evaluated against 30 human pathogens including 27 clinical strains and three isolates of marine origin for their possible implications on human health. The methyl ester of salvileucolide (**10**), salvileucolide-6,23-lactone (**11**), sclareol (**15**), and manool (**17**) were the most active against Gram-positive bacteria. The compounds were also tested for the inhibition of ATP production in purified mammalian rod outer segments. Terpenoids **10**, **11**, **15**, and **17** inhibited ATP production, while only **17** inhibited also ATP hydrolysis. Molecular modeling studies confirmed the capacity of **17** to interact with mammalian ATP synthase. A significant reduction of ATP production in the presence of **17** was observed in *Enterococcus faecalis* and *E. faecium* isolates.

The most evident challenge to the treatment of several infectious diseases is the increasing rate of bacterial resistance to several antibiotics.^1^ The development of new drugs has decreased alarmingly; in the past half century, only a few new classes of antibiotics have entered the clinic, and the elaboration of novel therapies is urgently required.^2−4^ The lipophilic extracts of plant surfaces were shown to possess antimicrobial activities,^5−7^ due to the secretion of defense compounds onto the cuticular layer.^8−10^ In a search for diterpenoids from *Salvia* species with activity against multidrug-resistant human clinical strains,^11−13^ the aerial parts of *Salvia tingitana* Etl. (Lamiaceae) were investigated. The species is an aromatic woody perennial shrub originating from North Africa and the Middle East and is now cultivated as an ornamental plant in different parts of the world.^14^*S. tingitana* could be extinct in North Africa,^15^ and the only known recent collection is from Saudi Arabia.^16^ For a long time the taxonomic interpretation of the species was not clear,^15−17^ but recent studies defined *S. tingitana* as a distinct species and separate from *S. sclarea* and other *Salvia* species that in the past have been considered as related to it.^14^

Herein we report the isolation and structure elucidation of compounds obtained from the CH_2_Cl_2_-soluble extract of the plant surface and their antimicrobial activity. The microbial species selected for the study were mainly Gram-positive species, belonging to the *Staphylococcus* and *Enterococcus* genera. *Staphylococci* and methicillin-resistant-*Staphylococci* (MRS), particularly *Staphylococcus aureus* (MRSA) and *Staphylococcus epidermidis* (MRSE), are normally present on human skin and mucosa. They are responsible for a wide range of mild to life-threatening infections. MRSA are considered to be major pathogens for humans, causing hospital- and community-acquired conditions, such as sepsis, pneumonia, skin and soft tissue infections, endocarditis, and many other serious ailments.^18,19^ In addition, MRSE, due to their ability to produce biofilms, can generate difficult-to-eradicate infections, like those occurring on prostheses, on intravenous catheters, or in carriers of cardiac valvular lesions.^19^ For their relevant etiological role in several clinical settings, *Enterococcus faecium* and *S. aureus* have been included in a particular group of drug-resistant pathogens, acronymically referred to as “ESKAPE” (*E. faecium*, *S. aureus*, *Klebsiella pneumoniae*, *Acinetobacter baumannii*, *Pseudomonas aeruginosa*, and *Enterobacter* spp.), against whom the search for new curative antibacterial agents has become critically urgent.^20^ The Gram-positive *Enterococcus* genus includes facultative anaerobic species that can normally inhabit the human intestine as commensals. After dispersion in the hospital environment they can survive in the wards for long periods and may easily contaminate patients and the surface of medical equipment.^21^*E. faecium* and *E. faecalis*, the two most relevant clinical species, are characterized by high levels of intrinsic and acquired antibiotic resistance, mainly expressed toward β-lactams and glycopeptides such as vancomycin (VRE).^21,22^ They are involved in several nosocomial conditions, including urinary tract infections, as well as in serious bacteraemias, endocarditis, and meningitis.^21^

In addition, these compounds were investigated for the modulation of ATP synthase activity. ATP synthase is associated directly or indirectly with various human diseases,^23,24^ and the search for natural and synthetic inhibitors of this protein complex may generate new lead compounds,^25−27^ including new antimicrobial agents.^24,28^ Modulation of ATP synthesis has been described as the underlying principle for the activity of various compounds against multidrug-resistant mycobacteria, Gram-positive pathogens^25,29^ including *S. aureus* and *Streptococcus pneumoniae,*(30) and fungi.^31^ ATP synthase is known to be conserved from bacteria to mitochondria and chloroplasts.^28,32,33^ Similar overall structures of ATP synthase monomers or dimers have been described in nonrelated organisms such as prokaryotes, yeasts, and mammalian species.^23^ The possibility of selective inhibition of the bacterial enzyme by modulation of specific bacterial subunits is at present considered at the base of pathogen ATP synthases as potential drug targets,^32^ and thus of the so-called sixth antibiotic target space.^23,34^ Given that the overall structure and energy transduction mechanism of the F-type ATP synthases are well conserved from bacteria to mammalians,^35−38^ purified mammalian rod outer segments (OS) were used as a subcellular system, allowing the rapid assay of the modulating action of the isolated compounds on the ectopic F_o_F_1_-ATP synthase.^39−41^ In fact, the OS are composed of a stack of membranous disks, naturally sealed vesicles expressing the molecular machinery for the complete oxidation of glucose, thereby comprising the tricarboxylic acid cycle,^42^ and the five complexes of respiration.^43,44^ This could provide some indication of a possible correlation between the antibacterial activity and the modulation of the ATP synthase, in view of deeper investigations. Docking, molecular dynamics (MD) simulation studies, and ligand–protein binding energy evaluations were used to analyze the interaction of the most active compound with ATP synthase. Finally, its effect on ATP production in bacterial cells was evaluated.

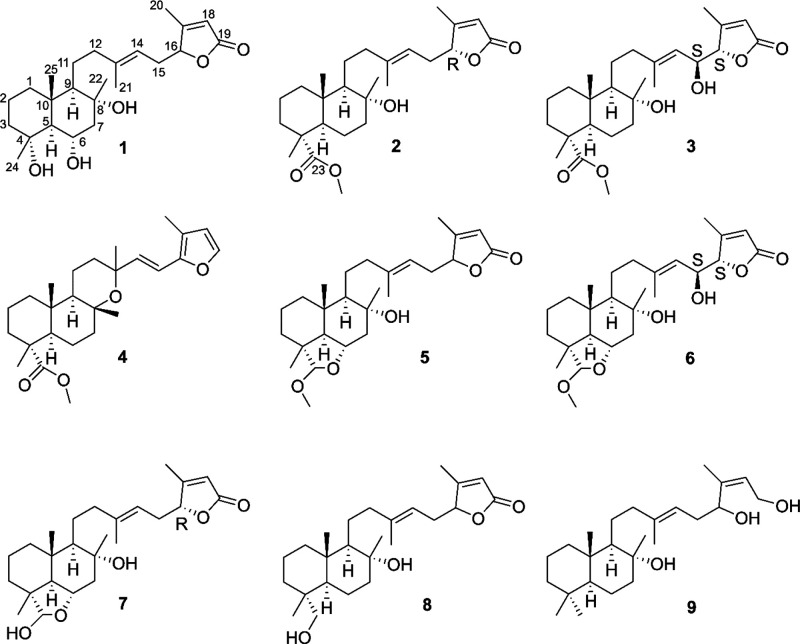



## Results and Discussion

The lipophilic extract of the plant surface of *S. tingitana* afforded one nor-sesterterpenoid (**1**), eight new sesterterpenoids (**2**–**9**), and five known sesterterpenoids, along with other known compounds including five labdane and one abietane diterpenoid, one sesquiterpenoid, and four flavonoids.

HRMS data of **1** showed a sodium adduct ion at *m*/*z* 429.2605 [M + Na]^+^ (calcd for C_24_H_38_O_5_Na^+^, 429.2611), consistent with a molecular formula of C_24_H_38_O_5_ for the parent molecule, and six indices of hydrogen deficiency. The IR data exhibited absorption bands at 3369, 1738, and 1644 cm^–1^, indicative of hydroxy, carbonyl, and olefinic groups.^45^ The ^1^H NMR data (Table 1) displayed signals corresponding to five methyls (δ_H_ 0.79, H_3_-25; δ_H_ 1.18, H_3_-22; δ_H_ 1.34, H_3_-24; δ_H_ 1.66, H_3_-21; δ_H_ 2.06, H_3_-20), two oxymethine groups (δ_H_ 3.97, ddd, *J* = 11.0, 10.0, 4.8 Hz, H-6; δ_H_ 4.89, t, *J* = 5.2, 5.2 Hz, H-16), and two protons of trisubstituted olefinic moieties (δ_H_ 5.06, H-14; δ_H_ 5.83, H-18). ^1^H–^1^H COSY and 1D TOCSY measurements allowed establishment of the spin systems C-1–C-3, C-5–C-7, C-9–C-12, and C-14–C-16. The NMR data and the index of hydrogen deficiency indicated that the structure was tricyclic. The deshielded shift of C-4 (δ_C_ 73.4), C-3 (δ_C_ 42.0), and C-5 (δ_C_ 59.7) suggested the presence of an oxygenated group at C-4, confirmed by the HMBC correlations of H_2_-2/C-4, H_2_-3/C-4, H-5/C-4, and H_3_-24/C-4. These data, and the comparison with related sesterterpenoids, led to the conclusion that **1** was a C-23 nor-sesterterpenoid. The location of a hydroxy group at C-6 was confirmed by the HMBC correlations of H-5/C-6, H-6/C-5, and H_2_-7/C-6. The presence of an α,β-unsaturated butenolide moiety^46,47^ at C-15 was inferred on the basis of the H-18 resonance, the C-19 carbonyl resonance (δ_C_ 172.6), the HMBC correlations of H_2_-15/C-17, H-16/C-18, H-18/C-16, H-18/C-17, H-18/C-19, and H_3_-20/C-16 and C-18, and the long-range COSY coupling between CH_3_-20 and H-18. The NOESY correlations between H-6, H_3_-22, H_3_-24, and H_3_-25 and the correlation of H-5 with H-9 indicated a trans-junction of the decalin system and a β-orientation of H-6 and CH_3_-24. The *E* configuration of the Δ^13(14)^ double bond was established from the ^13^C chemical shift of C-21.^48^ Thus, the structure of compound **1** was defined as (13*E*)-4α,6α,8α-trihydroxylabd-13(14),17(18)-dien-16,19-olide. Only a few nor-sesterterpenoids have been reported from species of *Salvia*.^49−51^ Compound **1** is the first C-23 nor-sesterterpenoid from a *Salvia* species.

**Table 1:** ^1^H and ^13^C NMR Data of Compounds **1**, **2**, and **3**^a^

	**1**			**2**			**3**		
position	δ_C_, type	δ_H_	HMBC	δ_C_, type	δ_H_	HMBC	δ_C_, type	δ_H_	HMBC
1	38.9, CH_2_	1.60^b^	2, 3, 5, 9, 10	38.7, CH_2_	1.67^b^	2, 3, 5, 10, 25	39.6, CH_2_	1.68^b^	2, 10, 24, 25
		1.05^b^			1.04, ddd (13.0, 12.5, 3.8)			1.07^b^	
2	18.6, CH_2_	1.62^b^	1, 3	17.6, CH_2_	1.61^b^	1, 3, 4	17.8, CH_2_	1.62^b^	1, 3, 4
		1.53^b^			1.54^b^			1.55^b^	
3	42.0, CH_2_	1.76^b^	1, 2, 4, 5, 24	37.0, CH_2_	1.73, m	1, 2, 4, 5, 24	37.2, CH_2_	1.73, m	2, 4, 5, 23, 24
		1.44^b^			1.53^b^			1.56^b^	
4	73.4, C			47.7, C			47.8, C		
5	59.7, CH	1.43^b^	1, 4, 6, 7, 9, 10, 24, 25	50.6, CH	1.77, dd (12.0, 2.3)	1, 4, 6, 7, 9, 10, 23, 24, 25	50.7, CH	1.79^b^	4, 5, 6, 7, 9, 10, 23, 24, 25
6	67.9, CH	3.97, ddd (11.0, 10.0, 4.8)	5	23.7, CH_2_	1.51^b^	5, 7, 8, 10	23.1, CH_2_	1.58^b^	5, 7, 8, 10
					1.35^b^			1.37^b^	
7	53.2, CH_2_	2.19, d (12.0, 4.8)	5, 6, 8, 9, 22	44.2, CH_2_	1.82, ddd (12.1, 3.2, 3.1)	5, 6, 8, 9	44.6, CH_2_	1.82^b^	5, 6, 8, 9
		1.63^b^			1.47, m			1.48^b^	
8	72.8, C			74.1, C			74.4, C		
9	59.2, CH	1.15, dd (4.1, 4.1)	8, 10, 11, 12, 25	61.3, CH	1.09^b^	1, 8, 10, 11, 12	61.5, CH	1.12^b^	1, 5, 7, 8, 10, 11
10	38.6, C			39.1, C			37.1, C		
11	22.9, CH_2_	1.59^b^	8, 9, 10, 12	23.4, CH_2_	1.52^b^	8, 9, 10, 12, 13	23.8, CH_2_	1.59^b^	8, 9, 12, 13
		1.37^b^			1.34^b^			1.38^b^	
12	42.1, CH_2_	2.11^b^	9, 11, 13, 14, 21	43.2, CH_2_	2.11^b^	9, 11, 13, 14, 21	43.2, CH_2_	2.19^b^	9, 11, 13, 14
		2.08^b^			2.09^b^			2.13^b^	
13	140.1, C			141.2, C			144.3, C		
14	115.8, CH	5.06, t (7.1, 7.1)	12, 15, 16, 21	116.2, CH	5.08, t (6.5, 6.5)	12, 15, 16, 21	121.9, CH	5.36, d (8.6)	12, 21
15	29.6, CH_2_	2.68, ddd (15.0, 7.1, 5.2)	13, 14, 16, 17	30.5, CH_2_	2.67, ddd (14.2, 6.5, 5.5)	13, 14, 16, 17	67.8, CH	4.65, dd (8.6, 1.0)	13, 14
		2.28, ddd (15.0, 7.1, 5.2),			2.32, ddd (14.2, 6.8, 5.5)				
16	83.7, CH	4.89, t (5.2, 5.2)	14, 15, 17, 18	84.5, CH	4.89, t (5.5, 5.5)	14, 15, 17, 18	87.1, CH	4.81, d (1.0)	14, 15
17	167.6, C			168.4, C			166.7, C		
18	116.4, CH	5.83, br s	16, 17, 19, 20	117.5, CH	5.85, s	16, 17, 19, 20	118.3, CH	5.91, s	16, 17, 19
19	172.6, CO			173.4, CO			173.3, CO		
20	13.3, CH_3_	2.06, s	16, 17, 18	14.1, CH_3_	2.06, s	16, 17, 18	14.8, CH_3_	2.15, s	16, 17, 18
21	16.0, CH_3_	1.66, s	12, 13, 14	16.5, CH_3_	1.66, s	12, 13, 14	17.3, CH_3_	1.77, s	12, 13, 14
22	24.3, CH_3_	1.18, s	7, 8, 9	23.9, CH_3_	1.11, s	7, 8, 9	24.6, CH_3_	1.13^b^	7, 8, 9
23				179.3, C			179.3, CO		
24	23.1, CH_3_	1.34, s	3, 4, 5	16.6, CH_3_	1.13, s	3, 4, 5, 23	16.6, CH_3_	1.14^b^	3, 4, 5, 23
25	15.8, CH_3_	0.79, s	1, 5, 9, 10	16.0, CH_3_	0.82, s	1, 5, 9, 10	16.5, CH_3_	0.84, s	1, 5, 9, 10
OMe				52.1, CH_3_	3.67, s	23	52.2, CH_3_	3.67, s	23

a Spectra were recorded in CDCl_3_, at 600 MHz (^1^H) and 150 MHz (^13^C). *J* values are in parentheses and reported in Hz; chemical shifts are given in ppm; assignments were confirmed by DQF-COSY, 1D-TOCSY, and HSQC experiments.

b Overlapped signal.

**Figure 1:**
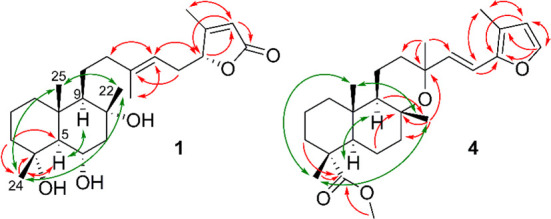
Selected HMBC (red) and NOESY (green) correlations for compounds **1** and **4** isolated from *S. tingitana*.

Compound **2** was obtained as a colorless, amorphous powder. A molecular formula of C_26_H_40_O_5_ was deduced from the HRESIMS data [*m*/*z* 433.2937 [M + H]^+^ (calcd for C_26_H_41_O_5_^+^, 433.2949)], indicating an index of hydrogen deficiency of seven. The ^1^H and ^13^C NMR data (Table 1) closely resembled those of the methyl ester of salvileucolide,^52^ with the exception of the presence of a methylene group at C-6 (δ_H_ 1.35, 1.51; δ_C_ 23.7) instead of a hydroxymethine. This was confirmed by the HMBC correlations of H_2_-6/C-5, C-7, C-8, C-10 and H_2_-7/C-6. *J* values of H-5 (δ_H_ 1.77, dd, *J* = 12.0, 2.3 Hz) and NOESY data confirmed the same relative configuration as for the methyl ester of salvileucolide.^52,53^ The NOESY correlations between H-5 and H-9 and between H_3_-22, H_3_-24, and H_3_-25 were consistent with a β-orientation of Me-22, Me-24, and Me-25 and with a trans-ring junction of the decalin system. Thus, compound **2** and its relative configuration were identified as (13*E*)-8α-hydroxy-23-carboxymethyllabd-13(14),17(18)-dien-16,19-olide.

A molecular formula of C_26_H_40_O_6_ for compound **3** was established from the HRESIMS data [*m*/*z* 471.2702 [M + Na]^+^ (calcd for C_26_H_40_O_6_Na^+^, 471.2717)], indicating seven indices of hydrogen deficiency. The ^13^C and ^1^H NMR spectroscopic data (Table 1) indicated a molecular structure similar to (13*E*)-6α,8α,(15*S*)-trihydroxy-23-carboxymethyllabd-13(14),17(18)-dien-(16*S*),19-olide,^47^ except for the presence of a methylene group at C-6 (δ_H_ 1.37, 1.58; δ_C_ 23.1). This was corroborated by the connectivity of H_2_-6 in the COSY experiment with H-5 (δ_H_ 1.79) and H_2_-7 (δ_H_ 1.48 and 1.82) and in the HMBC with C-5, C-7, C-8, and C-10, as well as by HMBC correlations of H-5/C-6 and H-7/C-6. The NOESY experiment showed correlations of H_3_-22 with H_3_-24 and H_3_-25 and of H-5 with H-9. The chemical shift of C-21 confirmed the *E* configuration of the Δ^13(14)^ double bond as reported for similar compounds.^47,48^ Thus, **3** and its relative configuration were identified as (13*E*)-8α,15-dihydroxy-23-carboxymethyllabd-13(14),17(18)-dien-16,19-olide.

Compound **4** was obtained as a colorless, amorphous powder. A molecular formula of C_26_H_38_O_4_ was established from the sodium adduct ion at *m*/*z* 415.2833 [M + Na]^+^ (calcd for C_26_H_38_O_4_^+^, 415.2843), indicative of seven indices of hydrogen deficiency. The IR data showed absorption bands for carbonyl (1726 cm^–1^), olefinic (1661 cm^–1^), and conjugated ether groups (1246 cm^–1^). The ^13^C NMR data (Table 2) exhibited 26 carbon resonances corresponding to six methyl, seven methylene, four methine (two of them were sp^2^ carbons), two quaternary, and two oxygenated tertiary carbons (δ_C_ 73.2 and 76.1), and, in addition, one carboxylic carbon and resonances for a furan ring. The ^1^H NMR data (Table 2) showed resonances of four olefinic protons (δ_H_ 6.16, H-15; δ_H_ 6.22, H-18; δ_H_ 6.26, H-14; δ_H_ 7.25, H-19) and a methoxy group (δ_H_ 3.66, −OMe). As four indices of hydrogen deficiency accounted for the carboxylic group and the three double bonds, compound **4** was tetracyclic. The NMR data suggested the presence of a manoyloxide scaffold.^54^ The location of the carboxymethyl group (δ_C_ 179.0) at C-23 was indicated by the chemical shifts of C-4, C-23, and C-24 and by HMBC correlations of H_2_-3, H-5, and H_3_-24 with C-23. The presence of a furan moiety (δ_C_ 116.8, C-16; δ_C_ 148.6, C-17; δ_C_ 114.7, C-18 and δ_H_ 6.22, d, *J* = 1.0 Hz, H-18; δ_C_ 140.9, C-19 and δ_H_ 7.25, d, *J* = 1.8 Hz, H-19) at C-15 was confirmed by HMBC correlations of H-14/C-16, C-17, and H-15/C-16. The NOESY experiment showed cross-peaks between H_3_-22, H_3_-24, and H_3_-25 and between H-5 and H-9, thereby confirming the relative configuration of the manoyloxide scaffold.^54^ The relative configuration at C-13 could not be established due to overlapping signals of H_3_-21 and H_3_-22. Thus, compound **4** was identified as (14*E*)-methylmanoyloxide-14,16,18-trien-19,16-oxide-23-carboxylate.

**Table 2:** ^1^H and ^13^C NMR Data of Compounds **4**, **5**, and **6**^a^

	**4**			**5**			**6**		
position	δ_C_, type	δ_H_	HMBC	δ_C_, type	δ_H_	HMBC	δ_C_, type	δ_H_	HMBC
1	38.7, CH_2_	1.66^b^	2, 3, 5, 9, 10, 25	40.9, CH_2_	1.64^b^	2, 3, 5, 9, 10, 25	42.0, CH	1.62^b^	2, 3, 5, 9, 10, 25
		1.00, ddd (13.0, 12.0, 2.7)			0.97, ddd (13.0, 12.8, 5.6)			0.90^b^	
2	17.8, CH_2_	1.63^b^	1, 3, 4	19.1, CH_2_	1.63^b^	1, 3, 4, 10	19.9, CH_2_	1.58^b^	1, 3, 4, 10
		1.54^b^			1.59^b^			1.53^b^	
3	37.1, CH_2_	1.74^b^	1, 2, 4, 5, 23, 24	31.4, CH_2_	1.57^b^	1, 2, 4, 5, 23, 24	32.4, CH_2_	1.51^b^	2, 4, 5
		1.54^b^			1.45^b^			1.38^b^	
4	47.7, C			43.5, C			44.5, C		
5	51.0, CH	1.80, dd (13.2, 1.9)	1, 3, 4, 6, 7, 9, 10, 23, 24, 25	56.5, CH	1.49, d (11.4)	4, 6, 7, 9, 10, 23, 24, 25	57.9, CH	1.44, d (11.4)	1, 3, 4, 6, 7, 9, 10, 24, 25
6	22.8, CH_2_	1.38, dddd (13.3, 13.2, 13.2, 3.1)	4, 5, 7, 8, 10	73.6, CH	3.72, ddd (11.4, 11.3, 4.4)	4, 5, 10, 23	74.8, CH	3.65, ddd (11.4, 11.3, 4.4)	4, 5, 10
		1.10^b^							
7	42.7, CH_2_	1.71^b^	5, 6, 8, 9, 22	51.1, CH_2_	2.36^b^	5, 6, 8, 9, 22	52.3, CH_2_	2.28, dd (11.2, 4.4)	5, 6, 8, 9, 22
		1.45^b^			1.50^b^			1.50^b^	
8	76.1, C			75.4, C			76.4, C		
9	58.6, CH	1.33, dd (11.5, 2.4)	1, 5, 8, 10, 11, 12, 25	60.0, CH	1.09, t (5.2, 5.2)	1, 7, 8, 10, 11, 22, 25	61.1, CH	1.06^b^	7, 8, 10, 11, 12, 25
10	36.4, C			36.4, C			36.4, C		
11	16.1, CH_2_	1.58^b^	8, 9, 10, 12, 13	22.9, CH_2_	1.58^b^	8, 9, 10, 12, 13	24.2, CH_2_	1.62^b^	8, 9, 10, 12, 13
		1.52^b^			1.37^b^			1.39^b^	
12	36.4, CH_2_	2.24, ddd (12.7, 2.9, 2.7)	9, 11, 13, 14	41.5, CH_2_	2.17, ddd (14.0, 8.4, 8.0)	9, 11, 13, 14, 21	42.9, CH_2_	2.18, ddd (14.2, 8.2, 8.2)	9, 11, 13, 14, 21
		1.55^b^			2.12, ddd (14.0, 8.8, 5.6)			2.06^b^	
13	73.2, C			141.4, C			145.1, C		
14	136.7, CH	6.26, d (16.5)	12, 13, 15, 16, 21	116.2, CH	5.02, t (7.1, 7.1)	12, 15, 16, 21	123.2, CH	5.16, d (8.6)	12, 21
15	112.6, CH	6.16, d (16.5)	13, 14, 16, 17	30.3, CH_2_	2.72, ddd (15.0, 7.1, 4.7)	13, 14, 16, 17	69.2, CH	4.66, dd (8.6, 3.5)	
					2.32^b^				
16	116.8, C			84.5, CH	4.91, t (4.7, 4.7)	14, 15, 17, 18, 19	88.0, CH	4.80, d (3.5)	14, 15, 17, 18
17	148.6, CH_3_			168.3, C			168.0, C		
18	114.7, CH	6.22, d (1.8)	16, 17, 19, 20	117.9, CH	5.88, s	16, 17, 19, 20	119.3, CH	5.84, s	16, 17, 19, 20
19	140.9, CH	7.25, d (1.8)	16, 17, 18	173.5, CO			174.4, CO		
20	10.2, CH_3_	2.06, s	17, 18	14.1, CH_3_	2.06, s	16, 17, 18	15.9, CH_3_	2.10, s	16, 17, 18
21	33.1, CH_3_	1.23, s	12, 13, 14	16.4, CH_3_	1.65, s	12, 13, 14	18.1, CH_3_	1.66, s	12, 13, 14
22	24.5, CH_3_	1.22, s	7, 8, 9	24.7, CH_3_	1.12, s	7, 8, 9	25.8, CH_3_	1.05, s	7, 8, 9
23	179.0, C			112.6, CH	4.43, s	4, 5, 6, 24	113.6, CH_3_	4.35, s	4, 5, 6, 24, 1′
24	16.4, CH_3_	1.12, s	3, 4, 5, 23	19.6, CH_3_	1.01, s	3, 4, 5, 23	20.1, CH_3_	0.94, s	3, 4, 5, 23
25	16.3, CH_3_	0.75, s	1, 5, 9, 10	15.4, CH_3_	0.83, s	1, 5, 9, 10	16.3, CH_3_	0.84, s	1, 5, 9, 10
OMe	52.0, CH_3_	3.66, s		54.9, CH_3_	3.34, s	23	55.8, CH_3_	3.27, s	23

a Spectra were recorded in CDCl_3_, at 600 MHz (^1^H) and 150 MHz (^13^C). *J* values are in parentheses and reported in Hz; chemical shifts are given in ppm; assignments were confirmed by DQF-COSY, 1D-TOCSY, and HSQC experiments.

b Overlapped signal.

The molecular formula of compound **5**, a colorless, amorphous powder, was established as C_26_H_40_O_5_ on the basis of the HRESIMS data [*m*/*z* 455.2798 [M + Na]^+^ (calcd for C_26_H_40_O_5_Na^+^, 455.2768)]. The ^1^H and ^13^C NMR data (Table 2) showed similarities with **2**, except for the presence of a 6,23-epoxide moiety, supported by HMBC correlations of H-6 (δ_C_ 73.6, C-6 and δ_H_ 3.72, ddd, *J* = 11.4, 11.3, 4.4 Hz) with C-4 and C-23 and HMBC correlations of H-23 (δ_C_ 112.6, C-23 and δ_H_ 4.43, s) with C-6. The relative configuration at C-4, C-5, C-6, C-9, and C-10 was established considering the coupling constants of H-5 (δ_H_ 1.49, d, *J* = 11.4 Hz), H-6 (δ_H_ 3.72, ddd, *J* = 11.4, 11.3, 4.4 Hz), and H-9 (δ_H_ 1.09, t, *J* = 5.2, 5.2 Hz) and the NOESY correlations of H-6 with H_3_-22, H_3_-24, and H_3_-25 and of H-5 with H-9 and H_3_-OMe/C-23. The *E* geometry of the Δ^13^ double bond was inferred from the ^13^C chemical shift of C-21. Thus, compound **5** was identified as (13*E*)-8α-hydroxy-23α-*O*-methyl-23,6α-epoxylabd-13(14),17(18)-dien-16,19-olide.

Compound **6** was isolated as a colorless, amorphous powder. The HRESIMS data showed a sodium adduct ion at *m*/*z* 471.2698 [M + Na]^+^ (calcd for C_26_H_40_O_6_Na^+^, 471.2717), which was indicative of a molecular formula of C_26_H_40_O_6_ and seven indices of hydrogen deficiency. The ^1^H and ^13^C NMR data (Table 2) indicated a close structural similarity with **5**. The only difference was the presence of a hydroxy group at C-15 (δ_H_ 4.66, dd, *J* = 8.6, 3.5 Hz, H-6; δ_C_ 69.2), which was corroborated by an HMBC correlation of H-16 to C-15 (Table 2). As for compound **5**, the relative configuration of **6** was established based on the NOESY interactions of H-6 with H_3_-22, H-23, H_3_-24, and H_3_-25 and on the ^13^C chemical shift of C-21. Compound **6** and its relative configuration were thus identified as (13*E*)-8α,15-dihydroxy-23α-*O*-methyl-23,6α-epoxylabd-13(14),17(18)-dien-16,19-olide.

Compound **7** had a molecular formula of C_25_H_38_O_5_ [*m*/*z* 441.2611 [M + Na]^+^ (calcd for C_25_H_38_O_5_Na^+^, 441.2612)] and seven indices of hydrogen deficiency. The NMR data (Table 3) closely resembled those of **5**, with the exception of the presence of a hydroxy group at C-23 (δ_H_ 4.43, s, H-23; δ_C_ 111.1) instead of a methoxy group in **5**. The location was corroborated by an HMBC correlation of H_3_-24/C-23. ROESY correlations of H-6 with H_3_-22, H_3_-24, and H_3_-25 and of H-23 with H_3_-24 and the ^13^C chemical shift of C-21 confirmed the same relative configuration as for compound **5**. Thus, the structure of **7** and its relative configuration were defined as (13*E*)-8α,23α-dihydroxy-23,6α-epoxylabd-13(14),17(18)-dien-16,19-olide.

**Table 3:** ^1^H and ^13^C NMR Data of Compounds **7**, **8**, and **9**^a^

	**7**			**8**			**9**		
position	δ_C_, type	δ_H_	HMBC	δ_C_, type	δ_H_	HMBC	δ_C_, type	δ_H_	HMBC
1	39.2, CH_2_	1.67^b^		39.6, CH_2_	1.60^b^	2, 3, 9, 10, 25	40.3, CH_2_	1.63^b^	2, 3, 5, 10, 25
		0.96^b^			0.93, ddd (13.3, 13.0. 3.3)			0.94^b^	
2	18.1, CH_2_	1.70^b^		17.9, CH_2_	1.62^b^	1, 3, 4	19.1, CH_2_	1.56^b^	1, 3, 4
		1.62^b^			1.49^b^			1.42^b^	
3	30.6, CH_2_	1.59^b^		35.3, CH_2_	1.46^b^	1, 2, 4, 5, 24	43.0, CH_2_	1.36^b^	1, 2, 3, 4
		1.47^b^			1.22^b^			1.14^b^	
4	42.2, C			37.8, C			39.0, C		
5	55.5, CH	1.51^b^		48.8, CH	1.24^b^	1, 3, 4, 6, 7, 9, 10, 23, 24, 25	56.5, CH	0.91^b^	1, 6, 7, 10, 23, 24
6	72.3, CH	3.70, dd (11.3, 11.1, 4.2)		20.4, CH_2_	1.58^b^	5, 7, 8, 10	21.8, CH_2_	1.63^b^	5, 7, 8, 10
					1.26^b^			1.24^b^	
7	49.8, CH_2_	2.38^b^		44.3, CH_2_	1.48^b^	5, 6, 8, 9, 22	44.6, CH_2_	1.83, dt (12.2, 3.1, 3.1)	5, 6, 8, 9
		1.57^b^			1.84, ddd (13.9, 3.0, 3.0)			1.36^b^	
8	74.1, C			74.0, C			74.3, C		
9	59.4, CH	1.13^b^	7, 8	60.6, CH	1.05, t (4.2, 4.2)	8, 10, 11, 12, 22, 25	61.6, CH	1.03, t (4.0, 4.0)	8, 10, 11, 12
10	35.2, C			39.2, C			38.9, C		
11	22.1, CH_2_	1.64^b^		23.4, CH_2_	1.53^b^	8, 9, 10, 12	23.4, CH_2_	1.46^b^	8, 9, 10, 12
		1.42^b^			1.32, dddd (14.1, 9.0, 5.4, 5.3)			1.37^b^	
12	41.2, CH_2_	2.14^b^		42.9, CH_2_	2.10^b^	9, 11, 13, 14, 21	43.2, CH_2_	2.09^b^	9, 11, 13, 14, 21
		2.12^b^			2.07^b^			2.05^b^	
13	139.8, C			141.2, C			140.4, C		
14	115.4, CH	5.03, t (7.2, 7.2)		116.4, CH	5.02, t (6.8, 6.8)	12, 15, 16, 21	119.8, CH	5.13, t (7.4, 7.4)	12, 15, 16, 21
15	29.2, CH	2.72, ddd (14.9, 7.2, 5.5)		30.2, CH_2_	2.70, ddd (14.9, 6.8, 4.9)	13, 14, 16, 17	34.5, CH	2.34, ddd (14.0, 7.4, 6.9)	14, 16, 17
		2.32^b^			2.31, ddd (14.9, 6.8, 4.9)			2.19, ddd (14.0, 7.4, 6.9)	
16	83.2, CH	4.91, t (5.5, 5.5)		84.5, CH	4.90, t (4.9, 4.9)	14, 15, 17, 18	70.5, CH	4.49, t (6.9, 6.9)	14, 15, 17, 18, 20
17	167.2, C			168.4, C			141.0, C		
18	116.5, CH	5.86, s		117.7, CH	5.87, s	16, 17, 19, 20	126.3, CH	5.50, dd (6.9, 6.8)	16, 19, 20
19	172.3, CO			173.5, CO			58.4, CH_2_	4.18, dd (12.6, 6.8)	17, 18
								4.04, dd (12.6, 6.9)	
20	13.2, CH_3_	2.07, s	16, 17, 18	14.1, CH_3_	2.06, s	16, 17, 18	18.4, CH_3_	1.75, s	16, 17, 18
21	16,6, CH_3_	1.66, s	12, 13, 14	16.0, CH_3_	1.66, s	12, 13, 14	16.7, CH_3_	1.64, s	12, 13, 14
22	24.0, CH_3_	1.15, s	7, 8, 9	23.9, CH_3_	1.12, s	7, 8, 9	24.1, CH_3_	1.11, s	7, 8, 9
23	111.1, C17	4.43, s		72.0, CH_2_	3.47, d (11.0)	3, 4, 5, 24	33.8, CH_3_	0.85, s	3, 4, 24
					3.06, d (11.0)				
24	17.3, CH_3_	1.00, s	3, 4, 5, 23	17.6, CH_3_	0.72, s	3, 4, 5, 23	15.8, CH_3_	0.78, s	3, 4, 5, 23
25	15.7, CH_3_	0.87, s	1, 5, 9, 10	16.5, CH_3_	0.83, s	1, 5, 9, 10	15.9, CH_3_	0.79, s	1, 5, 9, 10
OMe									

a Spectra were recorded in CDCl_3_, at 600 MHz (^1^H) and 150 MHz (^13^C). *J* values are in parentheses and reported in Hz; chemical shifts are given in ppm; assignments were confirmed by DQF-COSY, 1D-TOCSY, and HSQC experiments.

b Overlapped signal.

Compound **8** was obtained as a colorless, amorphous powder. A molecular formula of C_25_H_40_O_4_ was derived from the [M + Na]^+^ ion at *m*/*z* 427.2823 (calcd for C_25_H_40_O_4_Na^+^, 427.2819) in the HRESIMS, indicating seven indices of hydrogen deficiency. The NMR data (Table 3) were highly similar to those of **2**. The presence of a hydroxymethyl group (δ_H_ 3.47, d, *J* = 11.0 Hz and 3.06, d, *J* = 11.0 Hz, H_2_-23; δ_C_ 72.0) instead of the methoxycarbonyl in **2** was confirmed by HMBC correlations of H-5 with C-23 and of H_2_-23 with C-3, C-4, C-5, and C-24. The relative configuration of **8** was identical to that of **2**. Thus, **8** was identified as (13*E*)-8α,23-dihydroxylabd-13(14),17(18)-dien-16,19-olide.

The HRESIMS data of compound **9**, a colorless, amorphous powder, exhibited an [M + Na]^+^ ion at *m*/*z* 415.3170 (calcd for C_25_H_44_O_3_Na^+^, 415.3183), which was indicative of a molecular formula of C_25_H_44_O_3_ and four indices of hydrogen deficiency. The IR data of **9** showed absorption bands of hydroxy (3369 and 1022 cm^–1^) and olefinic groups (1663 and 1647 cm^–1^). The ^13^C NMR data (Table 3) exhibited 25 carbon resonances corresponding to six methyl, eight methylene, one hydroxymethylene (δ_C_ 58.4), four methine, one oxymethine (δ_C_ 70.5), four quaternary carbons, and one oxygenated tertiary carbon (δ_C_ 74.3). Two of the four degrees of hydrogen deficiency were accounted for by two double bonds (Table 3). Thus, the compound contained only two rings. This was corroborated by the absence of a carbonyl signal at C-19 as in **1** (δ_H_ 4.18, dd, *J* = 12.6, 6.8 Hz and 4.04, dd, *J* = 12.6, 6.9 Hz, H_2_-19; δ_C_ 58.4, C-19) and by the chemical shifts of H-16 (δ_H_ 4.49, t, *J* = 6.9, 6.9 Hz) and C-16 (δ_C_ 70.5), indicating the absence of the α,β-unsaturated butenolide moiety. COSY and 1D TOCSY experiments provided evidence of the spin systems H_2_-1–H_2_-3, H-5–H_2_-7, H_2_-11–H_2_-12, H-14–H-16, and H-18–H_2_-19. According to the HMBC correlations (Table 3), the 2D structure of **9** could be constructed. Determination of the relative configuration of **9** hinged upon the NOESY correlations involving H-5 with H-9 and H_3_-22 with H_3_-24 and H_3_-25 and upon H-14 (δ_H_ 5.13, t, *J* = 7.4, 7.4 Hz) and H-18 (δ_H_ 5.50, dd, *J* = 6.9, 6.8 Hz) coupling constants. Thus, compound **9** was established as a (13*E*)-labd-13(14),17(18)-dien-8α,16,19-triol.

In cases where enough material was available, the absolute configurations were studied by vibrational circular dichroism (VCD) and complemented by a Mosher ester analysis to determine the configuration at C-15 for compounds **3** and **6**. After derivatization of the C-15 carbinol moiety with methoxytrifluoromethylphenylacetic acid (MTPA, Mosher’s reagent), the ^1^H NMR chemical shifts of the resulting diastereomeric esters were compared.^55,56^ Owing to the anisotropic effect of the benzene ring, negative values (Δδ = δ_*S*_ – δ_*R*_) were obtained for H-14 (−0.16 for compounds **3** and **6**) and H_3_-21 (−0.01 for compounds **3** and **6**) of (*S*)-MTPA and (*R*)-MTPA esters of compound **3** and **6**, while positive values (Δδ = δ_*S*_ – δ_*R*_) were obtained for H-16 (+0.07 for compound **3** and +0.08 for **6**), H-18 (+0.14 for **3** and +0.13 for **6**), and H_3_-20 (+0.09 for **3** and +0.11 for **6**), indicating a (15*S*) absolute configuration (Figures S24 and S25 and S48 and S49, Supporting Information).

VCD spectra of compounds **2**, **3**, **6**, and **7** were recorded and compared to their calculated spectra at the B3LYP/6-31+G(d,p) level of theory (Figures S15, S23, S47, and S56, Supporting Information). Similarity indices *Sim*VA (vibrational absorption) and *Sim*VCD were calculated with VCD SpecTech^57^ using scaling factors between 0.8 and 1.2 (Figures S15, S23, S47, and S56, Supporting Information). The scaling factor corresponding to the maximal value of *Sim*VA calculated for all configurations was used to plot the calculated spectra. For compound **2**, the maximal *Sim*VCD value of the calculated spectrum for the (4*R*,5*R*,8*R*,9*R*,10*S*,16*R*) absolute configuration was 0.379, compared to 0.281 for (4*R*,5*R*,8*R*,9*R*,10*S*,16*S*). This suggested a better fit of (4*R*,5*R*,8*R*,9*R*,10*S*,16*R*). Visual evaluation of the experimental and calculated VCD spectra showed a significantly better fit of the bands at 1644, 1106, 1065, and 1039 cm^–1^. For compound **3**, the VCD similarity analysis gave no clear preference to any of the four calculated spectra (Figure S23, Supporting Information). As for compound **6**, it showed a clear preference for (4*R*,5*R*,6*S*,8*R*,9*R*,10*S*,15*R*,16*R*,23*S*) with a maximal *Sim*VCD value of 0.483. For compound **7**, both calculated VCD spectra showed an excellent fit, with maximal *Sim*VCD values of 0.468 for (4*R*,5*R*,6*S*,8*R*,9*R*,10*S*,16*S*,23*S*) and 0.369 for (4*R*,5*R*,6*S*,8*R*,9*R*,10*S*,16*R*,23*S*). Therefore, no assignment was possible. Since the results from the VCD data of compound **6** conflicted with those for the Mosher esters, VCD data were thoroughly reviewed. It was found that most of the vibrational bands originated from the decalin system. The stereocenters at C-15 and C-16 did not impact the VCD spectra to an extent enabling the determination of the absolute configuration. Therefore, VCD was not considered suitable for this type of compounds. Owing to a lack of material, a hydrolysis of the lactone rings followed by a Mosher ester analysis was not possible. For compounds **3** and **6**, comparison of the NMR data with those from reported congeners^46^ indicated the (16*S*) configuration. For compounds **2** and **7**, the configuration could be 16*R*, as previously described.^47,58^ Hence, the absolute configurations are proposed as (4*R*,5*R*,8*R*,9*R*,10*S*,16*R*) for compound **2**, (4*R*,5*R*,6*S*,8*R*,9*R*,10*S*,15*S*,16*S*) for **3**, (4*R*,5*R*,6*S*,8*R*,9*R*,10*S,*15*S*,16*S*,23*S*) for **6**, and (4*R*,5*R*,6*S*,8*R*,9*R*,10*S*,16*R*,23*S*) for **7**.

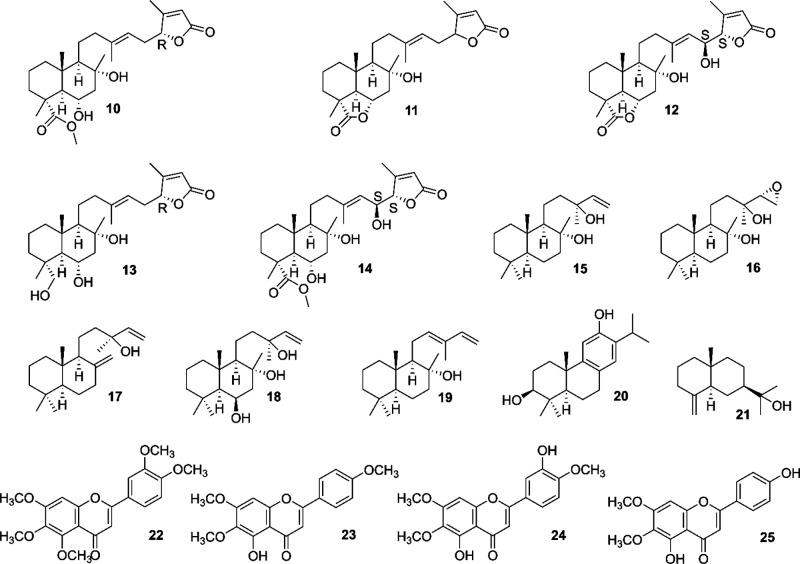



The known compounds were identified as the methyl ester of salvileucolide (**10**),^52^ salvileucolide-6,23-lactone (**11**),^52,59^ (15*S*,16*S,*13*E*)-8α,15-dihydroxylabd-13(14),17(18)-dien-23,6α-16,19-diolide (**12**),^47^ (16*R,*13*E*)-6α,8α,23-trihydroxylabd-13(14),17(18)-dien-16,19-olide (**13**),^47^ (15*S*,16*S,*13*E*)-6α,8α,15-trihydroxy-23-carboxymethyllabd-13(14),17(18)-dien-16,19-olide (**14**),^47^ sclareol (**15**),^60^ 14α-epoxysclareol (**16**),^61^ manool (**17**),^62^ 6β-hydroxysclareol (**18**),^61^ (12*Z*)-8α-12,14-labdadien-8-ol (**19**),^63^ hinokiol (**20**),^64^ β-eudesmol (**21**),^65^ 3′,4′,5,6,7-pentamethoxyflavone (**22**),^66^ salvigenin (**23**),^67^ eupatorin (**24**),^68^ and cirsimaritin (**25**).^69^

The extract, the *n*-hexane-insoluble and -soluble portions, and 13 semipurified fractions (I_a_–VI_a_ and I_b_–VII_b_) were tested against 12 representative clinical strains (Table S1, Supporting Information). The total extract showed MIC values of 128 μg/mL on *S. aureus*, *S. epidermidis*, *E. faecium*, and *E. faecalis* strains, while MICs > 128 μg/mL were found against the two other Gram-positive species (*Streptococcus agalactiae* and *Streptococcus pyogenes*), the four Gram-negative bacterial strains (*Escherichia coli*, *Proteus mirabilis*, *Moraxella catarrhalis*, and *K. pneumoniae*), and the two fungi (*Candida albicans* and *Candida glabrata*). Likewise, the *n*-hexane-insoluble and -soluble portions and the 13 semipurified fractions showed some activity against the Gram-positive strains but were inactive against the three Gram-negative bacteria and the two *Candida* strains (Table S1, Supporting Information). Nineteen compounds out of the 25, isolated in suitable quantities for biological assays, were analyzed for antibacterial activity by determining MIC values on a panel of 30 microbial clinical strains, mainly Gram-positive pathogens, belonging to several clinically relevant species of *Staphylococcus* and the *Enterococcus* genera. As depicted in Table 4, interesting results were obtained especially for *Staphylococci* and *Enterococci*, while MIC values above 128 μg/mL were obtained for *S. agalactiae*, *S. pyogenes*, the four Gram-negative species, and the two mycetes (data not shown). Interestingly, the antimicrobial activities observed were often uniform among the bacterial species and independent from the resistance patterns of the several isolates to classic antibiotics. Sclareol (**15**) and manool (**17**) displayed the lowest MIC values among the other pure compounds. Sclareol was active against several species of *Staphylococcus* and *Enterococcus* of clinical interest with very uniform MIC values, ranging from 32 to 64 μg/mL (Table 4). Manool, on the contrary, was particularly powerful against *Enterococcus*, reaching MIC values of 4 μg/mL on several species isolates (Table 4). Although we could not demonstrate any significant effects of these two labdane diterpenoids against the selected aerobic Gram-negative species, Souza and colleagues^70^ reported antimicrobial activity of the same compounds also against a few Gram-negative periodontal bacteria, probably because these organisms were endowed with an anaerobic metabolism. Moreover, we could not confirm any significant antimicrobial activity of manool against *S. aureus* as reported by Ulubelen,^71^ probably because of the clinical origin and the multidrug-resistant characteristics of the strains of *Staphylococcus* we employed.

**Table 4:** MIC Values for Compounds **2**–**8**, **10**–**17**, and **22**–**25**^a^

bacterial strains	**2**		**3**		**4**		**5**		**6**		**7**	**8**		**10**		**11**		**12**		**13**
*S. aureus* MB 18^d^	>128		128	(286)	128	(309)	128	(296)	128	(286)	>128	128	(317)	128	(286)	128	(308)	128	(296)	>128
*S. aureus* MB 188^d^	128	(296)	>128		>128		128	(296)	>128		>128	>128		>128		>128		>128		>128
*S. epidermidis* MB 165^d^	128	(296)	>128		128	(309)	64	(148)	>128		>128	128	(317)	128	(286)	128	(308)	128	(296)	>128
*S. epidermidis* MB 169^d^	>128		128	(286)	64	(155)	64	(148)	128	(286)	>128	>128		>128		>128		>128		>128
*S. saprophyticus* MB 41	128	(296)	128	(286)	128	(309)	128	(296)	128	(286)	>128	128	(317)	>128		>128		>128		>128
*S. capitis* MB 71^d^	64	(148)	128	(286)	64	(155)	32	(74.1)	128	(286)	>128	128	(317)	128	(286)	64	(154)	128	(296)	>128
*S. warneri* MB 74^d^	64	(148)	64	(143)	128	(309)	64	(148)	>128		>128	128	(317)	128	(286)	128	(308)	128	(296)	>128
*S. simulans* MB 94	>128		>128		>128		128	(296)	>128		>128	>128		>128		>128		>128		>128
*S. lugdunensis* MB 96	128	(296)	128	(286)	>128		128	(296)	>128		>128	>128		>128		128	(308)	>128		>128
*S. hemolyticus* MB 115^d^	128	(296)	>128		>128		128	(296)	>128		>128	>128		>128		>128		128	(296)	>128
*S. hominis* MB 124^d^	128	(296)	128	(286)	128	(309)	64	(148)	128	(286)	>128	128	(317)	128	(286)	>128		>128		>128
*E. faecalis* MB 1°	32	(74.1)	64	(143)	>128		128	(296)	64	(143)	>128	128	(317)	64	(143)	64	(154)	64	(148)	>128
*E. faecalis* MB 19°^e^	64.0	(148)	64	(143)	128	(309)	64	(148)	128	(286)	>128	32	(79.2)	128	(286)	128	(308)	128	(296)	>128
*E. faecalis* MB 51°^e^	128	(296)	128	(286)	128	(309)	128	(296)	64	(143)	>128	64	(158)	64	(143)	64	(154)	128	(296)	>128
*E. faecalis* MB 76	64	(148)	64	(143)	128	(309)	128	(296)	64	(143)	>128	64	(158)	128	(286)	128	(308)	128	(296)	>128
*E. faecium* MB 2	128	296	128	(286)	128	(309)	128	(296)	128	(286)	>128	128	(317)	128	(286)	>128		128	(296)	>128
*E. faecium* MB 3°^e^	64	(148)	64	(143)	128	(309)	128	(296)	64	(143)	>128	128	(317)	128	(286)	128	(308)	128	(296)	>128
*E. faecium* MB 152°	128	(296)	128	(286)	>128		64	(148)	128	(286)	>128	64	(158)	64	(143)	128	(308)	64	(148)	>128
*E. avium* MB 119	128	(296)	128	(286)	>128		128	(296)	64	(143)	>128	64	(158)	64	(143)	64	(154)	64	(148)	>128
*E. casseliflavus* MB 159°	128	(296)	64	(143)	128	(309)	64	(148)	128	(286)	>128	64	(158)	64	(143)	128	(308)	128	(296)	>128
*E. durans* MB 113	128	(296)	128	(286)	>128		128	(296)	>128		>128	>128		128	(286)	>128		128	(286)	>128
*E. gallinarum* MB 111°	64	(148)	64	(143)	128	(309)	128	(296)	64	(143)	>128	64	(158)	128	(286)	64	(154)	64	(148)	>128

bacterial strains	**14**		**15**		**16**		**17**		**22**		**23**		**24**		**25**		**O**^b^		**V**^c^	
*S. aureus* MB 18^d^	128	(276)	32	(104)	128	(395)	>128		>128		128	(390)	>128		>128		256	(638)	n.t.	
*S. aureus* MB 188^d^	>128		32	(104)	128	(395)	>128		128	(344)	>128		>128		>128		512	(1275)	n.t.	
*S. epidermidis* MB 165^d^	128	(276)	64	(207)	128	(395)	>128		>128		>128		>128		>128		256	(638)	n.t.	
*S. epidermidis* MB 169^d^	>128		64	(207)	128	(395)	>128		128	(344)	128	(390)	>128		>128		256	(638)	n.t.	
*S. saprophyticus* MB 41	>128		32	(104)	128	(395)	>128		>128		>128		>128		>128		1	(2.49)	n.t.	
*S. capitis* MB 71^d^	128	(276)	32	(104)	128	(395)	>128		128	(344)	128	(390)	>128		128	(407)	256	(638)	n.t.	
*S. warneri* MB 74^d^	64	(138)	32	(104)	64	(198)	>128		128	(344)	128	(390)	>128		>128		128	(319)	n.t.	
*S. simulans* MB 94	>128		64	(207)	128	(395)	>128		>128		>128		>128		>128		0.25	(0.62)	n.t.	
*S. lugdunensis* MB 96	>128		32	(104)	64	(198)	>128		>128		>128		>128		128	(407)	0.5	(1.25)	n.t.	
*S. hemolyticus* MB 115^d^	128	(276)	64	(207)	128	(395)	>128		>128		>128		>128		>128		16	(39.9)	n.t.	
*S. hominis* MB 124^d^	64	(138)	32	(104)	128	(395)	>128		128	(344)	128	(390)	>128		>128		16	(39.9)	n.t.	
*E. faecalis* MB 1°	64	(138)	32	(104)	32	(99)	16	(55)	32	(86)	64	(195)	64	(186)	64	(204)	n.t.		32	(22.1)
*E. faecalis* MB 19°^e^	128	(276)	32	(104)	64	(198)	32	(110)	128	(344)	64	(195)	64	(186)	128	(407)	n.t		32	(22.1)
*E. faecalis* MB 51°^e^	64	(138)	64	(207)	32	(99)	16	(55)	64	(172)	128	(390)	128	(372)	128	(407)	n.t		64	(44.2)
*E. faecalis* MB 76	128	(276)	64	(207)	64	(198)	32	(110)	128	(344)	128	(390)	64	(186)	128	(407)	n.t		128	(88.3)
*E. faecium* MB 2	128	(276)	32	(104)	64	(198)	4	(14)	128	(344)	128	(390)	128	(372)	128	(407)	n.t		2	(1.38)
*E. faecium* MB 3°^e^	128	(276)	32	(104)	32	(99)	8	(28)	128	(344)	64	(195)	>128		64	(204)	n.t		128	(88.3)
*E. faecium* MB 152°	64	(138)	64	(207)	64	(198)	8	(28)	64	(172)	64	(195)	128	(372)	127	(407)	n.t		256	(177)
*E. avium* MB 119	64	(138)	64	(207)	32	(99)	8	(28)	128	(344)	128	(390)	64	(186)	64	(204)	n.t		1	(0.69)
*E. casseliflavus* MB 159°	128	(276)	64	(207)	64	(198)	16	(55)	64	(172)	128	(390)	128	(372)	128	(407)	n.t		4	(2.76)
*E. durans* MB 113	64	(138)	32	(104)	64	(198)	16	(55)	128	(344)	64	(195)	>128		128	(407)	n.t		1	(0.69)
*E. gallinarum* MB 111°	128	(276)	64	(207)	32	(99)	4	(14)	64	(172)	64	(195)	64	(186)	32	(102)	n.t		16	(11)

a MIC values, expressed in μg/mL and micromolar concentration (μM), of the pure compounds on the selected bacterial strains.

b Oxacillin.

c Vancomycin.

d Methicillin-resistant *Staphylococcus* strain; vancomycin-resistant *Enterococcus* strain.

e Strain isolated from seawater of the Ligurian west coast (Italy); n.t. not tested.

**Figure 2:**
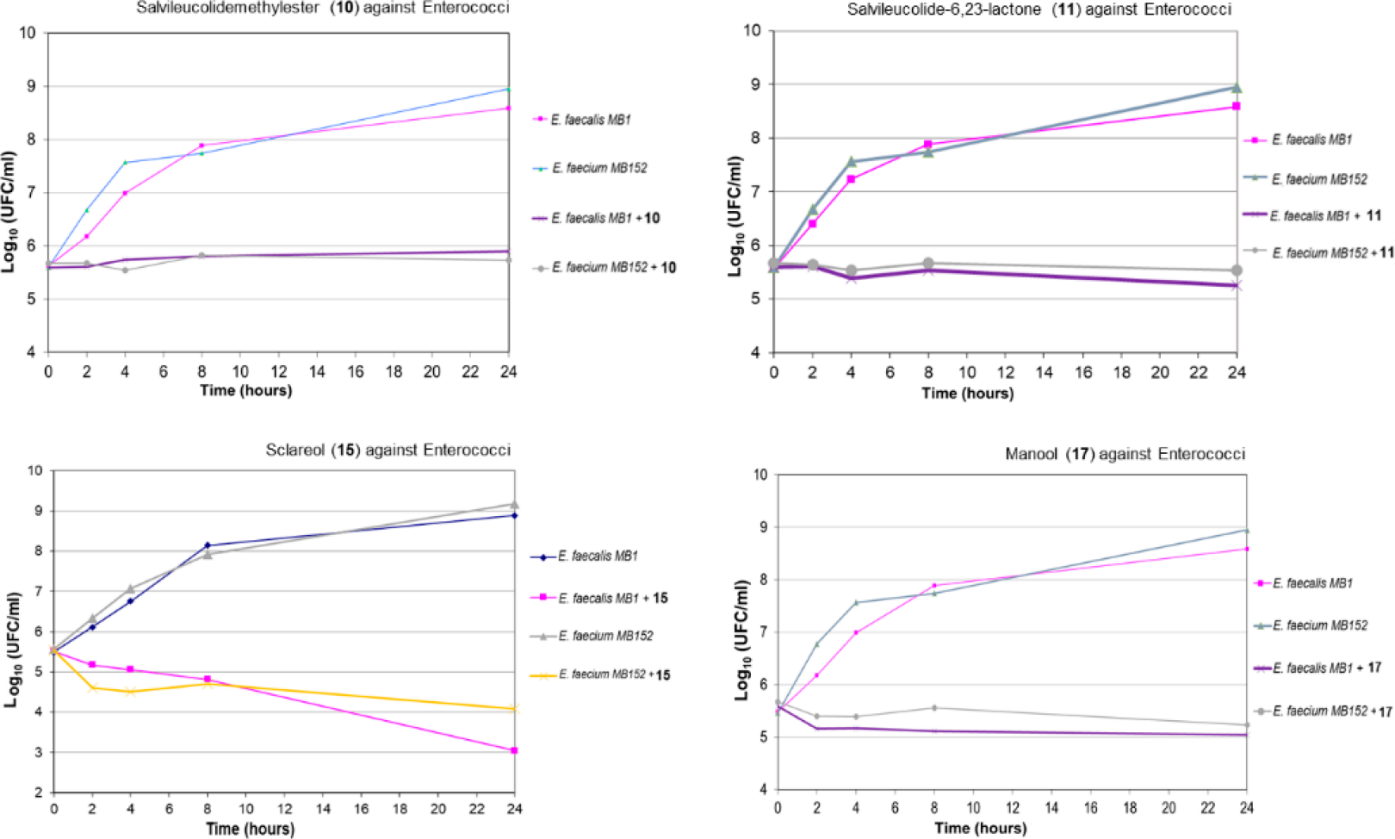
Effect of the methyl ester of salvileucolide (**10**), salvileucolide-6,23-lactone (**11**), sclareol (**15**), and manool (**17**) on viable cell number of selected susceptible Enterococcal strains. Time-kill curves were recorded in the absence or presence of the selected compounds at a concentration of 4 × MIC.

For sclareol (**15**), manool (**17**), the methyl ester of salvileucolide (**10**), and salvileucolide-6,23-lactone (**11**), the mechanism of action on the most clinically relevant and susceptible bacteria (*S. aureus*, *S. epidermidis*, *E. faecium*, and *E. faecalis*) was investigated. Time killing curves for representative resistant and multiresistant isolates are shown in Figure 2 and Figure S74 (Supporting Information). The four compounds were found to be bacteriostatic, as they prevented the growth of the starting inoculum or produced a decrease of bacterial count by 1 to 2 orders of magnitude within 24 h.

The *n*-hexane-insoluble and -soluble fractions, the 13 semipurified fractions (I_a_–VI_a_ and I_b_–VII_b_), and the 19 pure compounds were investigated for the modulation of ATP synthase activity. Resveratrol, a known inhibitor of rod OS ATP synthase, was used as a positive control.^40^ The data were verified by one-way ANOVA (performed in MATLAB 2019a), and differences among groups evaluated with the Bonferroni test (*p* < 0.05) (Figures S75 and S76, Supporting Information). The extract and most semipurified fractions showed activity (Figure S75, Supporting Information). ANOVA singled out five groups among the 19 compounds. The group with the most interesting activity comprised compounds **10**, **11**, **15**, and **17**, which showed an inhibition of ATP production of 60%, 79%, 70%, and 60%, respectively. The differences within this group were not statistically significant. The obtained data, showing inhibition of ATP production in the OS (Figure S76, Supporting Information) by the four pure compounds, could suggest an inhibitory action on the oxidative phosphorylation.^23,40,72^

The inhibition of ATP hydrolysis activity was also evaluated, as some plant metabolites were shown to be able to hinder also the clockwise rotation, which causes the reversal activity of the enzyme.^39,73^ The effects of extract, fractions, semipurified fractions, and pure compounds on this activity are shown in Figures S77 and S78 (Supporting Information). At a concentration of 80 μg/mL, manool (**17**) inhibited ATP hydrolysis by 92%. ANOVA analysis showed that this compound had a mode of action that was distinctly different from the others (Figure S78). The ability to inhibit also ATP hydrolysis activity could indicate that the modulating effect on ATP synthesis is not merely due to membrane uncoupling.^38,39^

**Figure 3:**
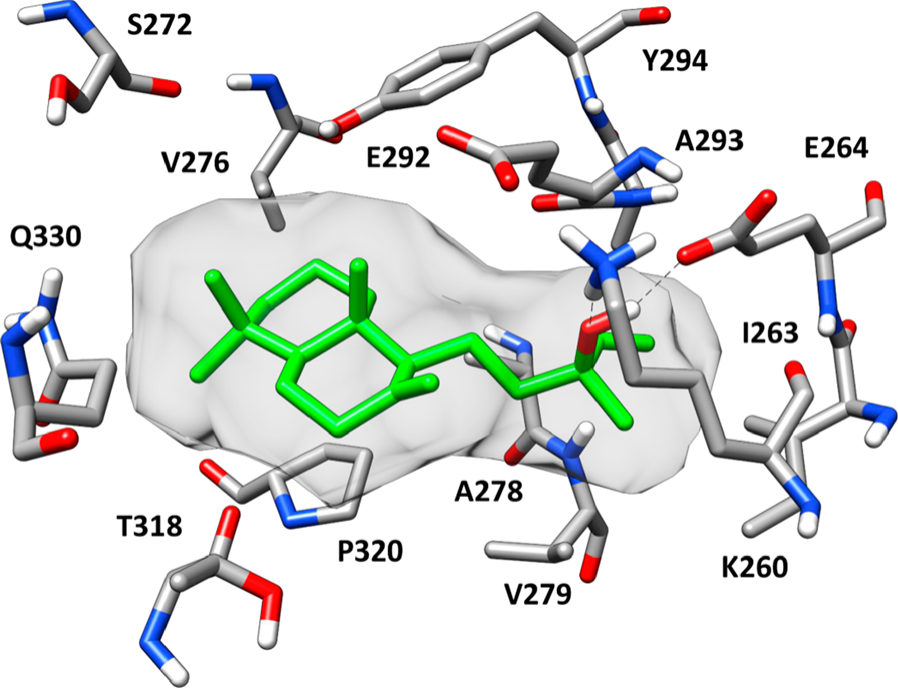
Minimized average structure of manool (**17**) complexed with F1-ATPase in binding mode 3. Hydrogen bonds are represented as black dashed lines. The ligand molecular surface is shown in gray.

To evaluate how **17** could interact with ATP synthase, docking, MD simulation studies, and ligand–protein binding energy evaluations were carried out. The analysis focused on the F_1_ catalytic domain of the protein (F_1_-ATPase), since several plant compounds such as resveratrol, piceatannol, and quercetin are known to interact with F_1_-ATPase, inhibiting ATP synthesis and hydrolysis. Indeed, X-ray structures of bovine F_1_-ATPase in complex with these compounds revealed a common binding site located among the α and β subunits of the protein, constituting the crown domain, and the C-terminal tip of the γ-subunit that is known to rotate inside the crown domain in association with ATP synthesis and hydrolysis. These ligands are thus supposed to inhibit ATP synthase activity by impeding this rotation, thus disrupting the catalytic machinery of the protein.^39^ For this reason, manool was docked into the X-ray structure of bovine F_1_-ATPase in complex with quercetin (PDB code 2JJ2)^39^ using a thorough AUTODOCK^74^ procedure that produced good results in both virtual screening and pose prediction studies.^75,76^ The docking protocol generated 200 different docking solutions, which were clustered based on their reciprocal root-mean square (RMSD) deviation using a threshold of 2.0 Å (see Experimental Procedures for details), thus producing a total of three different clusters of poses. The three corresponding ATPase-manool complexes were studied through a 30 ns MD simulation protocol in order to evaluate the stability of the binding modes predicted by docking. The results were analyzed in terms of RMSD of the ligand disposition during the simulation with respect to its coordinates in the starting complex. The analysis highlighted a high stability for pose 3, in which the ligand maintained an average RMSD of about 1.9 Å during the whole simulation (Figure S79, Supporting Information). On the contrary, the other two binding poses predicted by docking did not show enough stability. In both cases the ligand moved considerably from its initial binding disposition, as demonstrated by the high RMSD of its coordinates with respect to the starting pose that reached values around 9 - 10 Å. On the basis of these results, we could already consider both pose 1 and 2 as unreliable binding dispositions with respect to pose 3. However, in order to evaluate the different binding modes from an energetic point of view, relative binding free energy evaluations were performed on all three ATPase-manool complexes with the aim of identifying the most energetically reliable binding mode.^77^ Ligand–protein binding energies were calculated using the molecular mechanics Poisson–Boltzmann surface area (MM-PBSA) method^78^ on the MD trajectories relative to the last 15 ns of simulation (Table S2, Supporting Information). The analysis clearly confirmed the reliability of pose 3, whose estimated ligand–protein binding affinity (−21.4 kcal/mol) exceeded by about 9–12 kcal/mol those evaluated for pose 1 and pose 2 (Table S2, Supporting Information). Figure 3 shows the minimized average structure of F_1_-ATPase complexed with the manool in binding mode 3, as obtained from the last 15 ns of MD simulation. Owing to its lipophilic nature, the ligand predominantly forms hydrophobic interactions with the binding site residues. The bicyclic core of the ligand strongly interacts with P320, as well as with V276, T318, and Q330, while its lateral chain shows lipophilic interactions with A278, V279, A293, and I263, constituting a small subpocket together with K260, E264, and E292. Interestingly the terminal vinyl group of manool shows an NH−π interaction with the backbone nitrogen of A278.^79^ Despite the fact that the polar portion of the ligand is limited to its hydroxy group, this moiety is able to establish strong H-bonds with both K260 and E264 that account for a non-negligible contribution to the total ligand–protein binding energy (Table S2, Supporting Information) and probably promotes the stability of the binding pose by providing the ligand with a good anchoring point. The whole docking/MD simulation and ligand–protein binding energy evaluation protocol was also validated using the reference X-ray structure of bovine F_1_-ATPase in complex with quercetin (PDB code 2JJ2). The bound ligand was first subjected to a self-docking study using the same docking protocol employed for manool; in this case, two different clusters of poses were generated. The two corresponding ATPase-quercetin complexes were evaluated through the MD protocol and analyzed in terms of RMSD of the ligand disposition during the simulation with respect to its coordinates in the starting complex. As shown in Figure S80, pose 2 showed strong stability, with an average RMSD of about 1.5 Å, while pose 1 diverged from the initial docking solution of about 6 Å. The binding free energy evaluation performed on the two complexes confirmed pose 2 as the most reliable binding mode from both the qualitative and quantitative point of view (Table S3). The binding mode predicted for quercetin by our computational protocol was very similar to the experimental disposition of the ligand (Figure S81), with an RMSD between the two binding modes of around 2 Å. These results confirmed the reliability of the whole computational workflow applied for predicting the binding mode of manool into F_1_-ATPase.

**Figure 4:**
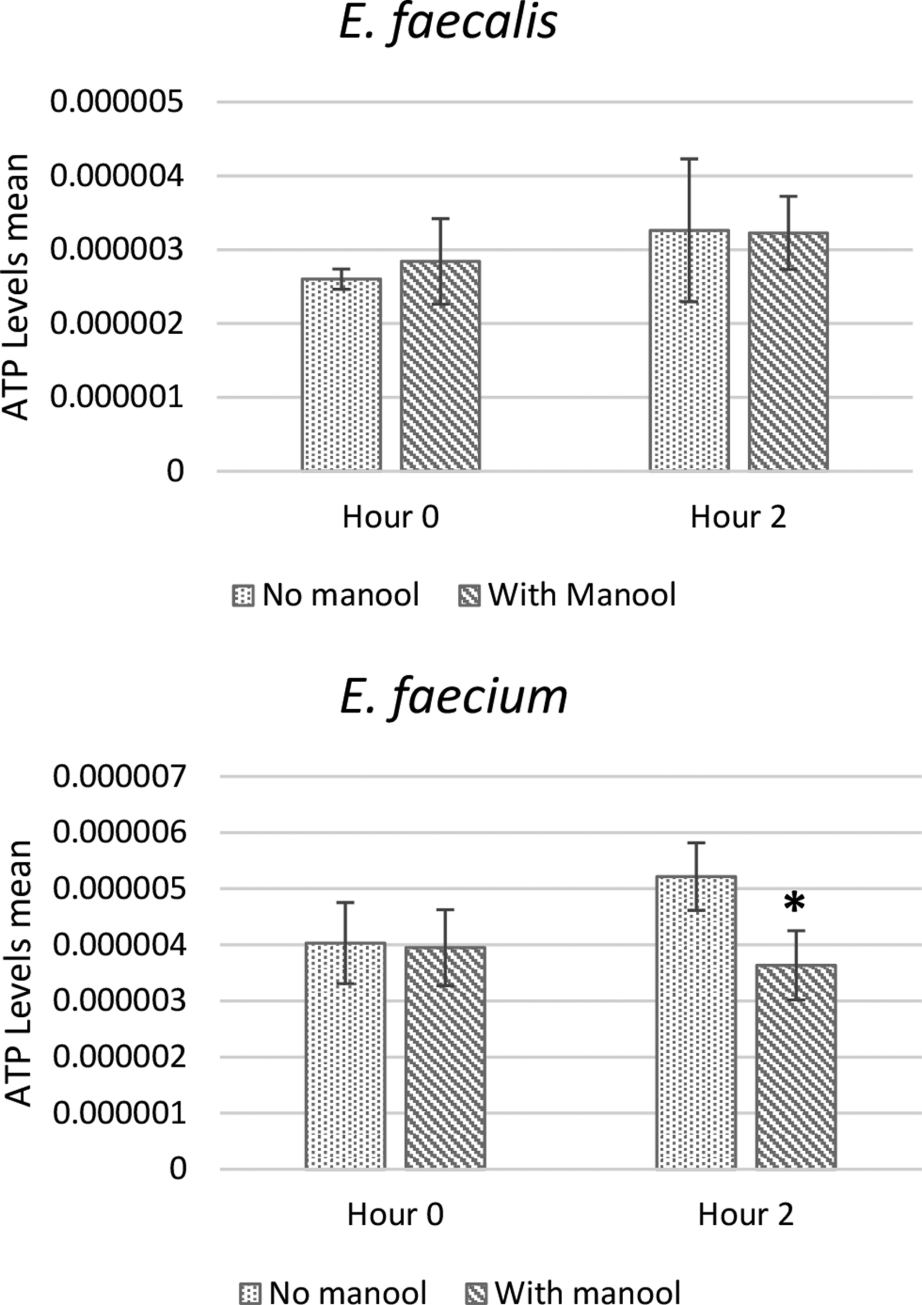
ATP levels determined by measuring luminescence levels and comparing with an ATP standard curve. Amount of ATP (pmol/cfu) produced by *E. faecalis* MB 1 (VRE) and *E. faecium* MB 152 (VRE) in the absence and in the presence of manool (**17**) (5 × MIC) at time of inoculum and after 2 h of incubation at *T* = 37 °C. Results are expressed as mean ± SD of three separate experiments, with three replicates per experiment. Statistically significant differences between treatment and control groups were determined using Student’s *t* test (*p* < 0.05).

Finally, the ATP production in the presence or absence of manool (**17**) by *E. faecalis* MB 1 (VRE) and *E. faecium* MB 152 (VRE) was assessed in a whole-cell assay, where the bacteria were supplied with nutrients, after an incubation of 2 h. This timing was chosen, as the duplication time of *Enterococcus* spp. is around 30 min.^80^ A significant reduction of the ATP amount of bacterial cells was observed in *E. faecium* (inhibited by 30%) (Figure 4). The data on ability to regenerate ATP of bacteria are to be considered with caution, as the pool of steady state ATP is dependent on many processes (glycolysis, substrate-level phosphorylation, oxidative phosphorylation, nutrient uptake systems) affecting its consumption and production. Our results show that a correlation between the in vivo antibacterial effect and the modulation of ATPase activity could be hypothesized for manool (**17**), and this could deserve further study.

## Experimental Section

### General Experimental Procedures

Optical rotations were measured with a PerkinElmer 241 polarimeter (PerkinElmer, Inc. Waltham, MA, USA) equipped with a sodium lamp (589 nm) and a 10 cm microcell. UV spectra were measured in CH_3_OH on a Chirascan CD spectrometer (Applied Photophysics, Leatherhead, UK), using 110 QS 1 mm path precision cells (Hellma Analytics, Müllheim, Germany). Data analysis was done with Pro-Data V2.4 software. IR and VCD spectra were recorded in CDCl_3_ on a Bruker PMA 50 accessory coupled to a Tensor 27 Fourier transform infrared spectrometer (Billerica, USA). A photoelastic modulator (Hinds PEM 90, Hinds Instruments, Hillsboro, USA) set at l/4 retardation was used to modulate the handedness of the circular polarized light. Demodulation was performed by a lock-in amplifier (SR830 DSP, Stanford Research System, Sunnyvale, CA, USA). An optical low-pass filter (<1800 cm^–1^) in front of the photoelastic modulator was used to enhance the signal/noise ratio. Solutions of 5–9 mg in 150 μL of CDCl_3_ were prepared and measured in a transmission cell equipped with CaF_2_ windows and a 200 μm spacer. Artifacts were eliminated by subtracting the VCD spectrum of the pure solvent (reference) from the VCD spectrum of the compound. For both the sample and the reference, ca. 24 000 scans at 4 cm^–1^ resolution were averaged. FTIR spectra were recorded as films or KBr pellets on a PerkinElmer System 2000 instrument (PerkinElmer). NMR experiments were performed on a Bruker DRX-600 spectrometer (Bruker BioSpin GmBH, Rheinstetten, Germany) equipped with a Bruker 5 mm TCI CryoProbe at 300 K and a Bruker DRX-400 spectrometer. All 2D NMR spectra were acquired in CDCl_3_, and standard pulse sequences and phase cycling were used for TOCSY, COSY, ROESY, NOESY, HSQC, and HMBC spectra. The NMR data were processed using UXNMR software. The ROESY spectra were acquired with *t*_mix_ = 400 ms. HRESIMS data were acquired in the positive ion mode by an LTQ Orbitrap XL mass spectrometer (Thermo Fisher Scientific, San Jose, CA, USA). The Orbitrap mass analyzer was calibrated according to the manufacturer’s directions by using a mixture of caffeine, methionine-arginine-phenylalanine-alanine-acetate (MRFA), sodium dodecyl sulfate, sodium taurocholate, and Ultramark 1621. Data were collected and analyzed using the software provided by the manufacturer. MPLC chromatography was performed on a spot liquid chromatography system (Armen Instrument, Saint Ave, France) with normal phase Si60 Cartridges Supervarioflash and LiChroprep RP-18 (40–63 μm) (Merck, Darmstadt, Germany). Silica gel 60 F_254_ coated aluminum sheets (Merck, 20 × 20 cm, 0.2 mm layer thickness) were used for TLC. CHCl_3_–CH_3_OH–HCOOH (10:0.5:0.1) was used as mobile phase, and spots were detected by spraying with 50% H_2_SO_4_, followed by heating. Semipreparative HPLC was carried out using a Waters W600 pump equipped with a Rheodyne Delta 600 injector, a 2414 refractive index detector, and a 2998 photodiode array detector (all Waters Corporation, Milford, MA, USA). A C_18_ column, SymmetryPrep C_18_, 7.8 × 300 mm i.d., 7 μm particle size (Waters) was used, at room temperature, flow rate 2.0 mL/min, sample loop 100 μL, eluents A: H_2_O, B: CH_3_OH, gradient: 5% to100% B in 61 min, 100% B to 75 min.

### Plant Material

The fresh aerial parts of a commercial specimen of *S. tingitana*(81) were obtained from CREA FSO San Remo, Italy, in June 2015. The plant material was identified by Prof. Ammar Bader, and a voucher specimen (UQU-IT-2019/1) was deposited in the Laboratory of Pharmacognosy at Umm Al-Qura University, Saudi Arabia.

### Extraction and Isolation

Fresh aerial parts (10.3 kg) of *S. tingitana* were immersed in CH_2_Cl_2_ for 20 s as previously described,^82^ to afford 103.0 g of exudate. The exudate was partitioned with *n*-hexane to afford an *n*-hexane-soluble (85.8 g) and an *n*-hexane-insoluble portion (17.7 g) (see Supporting Information for details). The *n*-hexane-insoluble portion was chromatographed in aliquots of 1.0 g on Sephadex LH-20 to afford six main fractions (FI_a_–FVI_a_): FI_a_ (0.2 g) with waxy compounds, FII_a_ (1.4 g), FIII_a_ (8.6 g), FIV_a_ (3.9 g), FV_a_ (1.6 g), and FVI_a_ (0.6 g). The *n*-hexane-soluble portion was chromatographed in aliquots of 1.0 g on Sephadex LH-20 to afford seven main fractions (FI_b_–FVII_b_): FI_b_ (3.1 g) with waxy compounds, FII_b_ (5.6 g), FIII_b_ (14.7 g), FIV_b_ (31.4 g), FV_b_ (16.1 g), FVI_b_ (3.2 g), and FVII_b_ (0.8 g). The main fractions were separated by repeated CC on silica gel (MPLC; monitoring by TLC) with a mixture of *n*-hexane–CHCl_3_ and mixtures of CHCl_3_–CH_3_OH. The compounds were purified by semipreparative HPLC. Particularly, the separation of the main fractions originating from the *n*-hexane-insoluble portion afforded the following. FII_a_: **3** (1.6 mg; *t*_R_ = 66.5 min) and **10** (4.7 mg; *t*_R_ = 68.0 min); FIII_a_: **1** (2.1 mg; *t*_R_ = 64.0 min), **2** (24.6 mg; *t*_R_ = 71.0 min), **3** (7.2 mg; *t*_R_ = 66.5 min), **5** (43.7 mg; *t*_R_ = 69.0 min), **6** (35.2 mg; *t*_R_ = 66.0 min), **7** (16.4 mg; *t*_R_ = 65.0 min), **9** (4.6 mg; *t*_R_ = 78.0 min), **10** (25.7 mg; *t*_R_ = 68.0 min), **11** (47.7 mg; *t*_R_ = 61.0 min), **12** (7.5 mg; *t*_R_ = 60.0 min), **13** (4.3 mg; *t*_R_ = 67.0 min), **14** (84.8 mg; *t*_R_ = 61.5 min), **22** (4.5 mg; *t*_R_ = 67.5 min), **23** (5.4 mg; *t*_R_ = 70.5 min), and **24** (9.8 mg; *t*_R_ = 64.5 min); FIV_a_: **3** (2.6 mg; *t*_R_ = 66.5 min), **6** (2.0 mg; *t*_R_ = 66.0 min), **8** (2.1 mg; *t*_R_ = 70.0 min), **14** (8.1 mg; *t*_R_ = 61.5 min), **15** (21.0 mg; *t*_R_ = 75.0 min), **16** (12.3 mg; *t*_R_ = 74.0 min), and **25** (4.6 mg; *t*_R_ = 60.5 min); FV_a_: **23** (13.3 mg; *t*_R_ = 70.5 min). The separation of the main fractions originating from the *n*-hexane-soluble portion afforded the following. FII_b_: **2** (6.6 mg; *t*_R_ = 71.0 min), **3** (5.4 mg; *t*_R_ = 66.5 min), **8** (2.8 mg; *t*_R_ = 70.0 min), **10** (15.5 mg; *t*_R_ = 68.0 min), and **11** (24.1 mg; *t*_R_ = 61.0 min); FIII_b_: **2** (27.4 mg; *t*_R_ = 71.0 min), **4** (13.7 mg; *t*_R_ = 84.0 min), **15** (201.3 mg; *t*_R_ = 75.0 min), **22** (1.6 mg; *t*_R_ = 67.5 min) and **23** (7.4 mg; *t*_R_ = 70.5 min); FIV_b_: **1** (1.7 mg; *t*_R_ = 64.0 min), **8** (8.6 mg; *t*_R_ = 70.0 min), **10** (5.9 mg; *t*_R_ = 68.0 min), **11** (3.9 mg), **12** (3.2 mg; *t*_R_ = 60.0 min), **13** (6.0 mg; *t*_R_ = 67.0 min), and **14** (29.1 mg; *t*_R_ = 61.5 min), **15** (1.25 g; *t*_R_ = 75.0 min), **17** (5.6 mg; *t*_R_ = 82.0 min) and **23** (12.6 mg; *t*_R_ = 70.5 min); FV_b_: **13** (2.8 mg; *t*_R_ = 67.0 min), **15** (20.1 mg; *t*_R_ = 75.0 min), **16** (2.1 mg), **17** (34.6 mg; *t*_R_ = 82.0 min), **18** (4.9 mg; *t*_R_ = 62.0 min), **19** (1.6 mg; *t*_R_ = 81.0 min) and **21** (3.0 mg; *t*_R_ = 76.0 min); FVI_b_: **13** (1.1 mg; *t*_R_ = 67.0 min) and **20** (2.7 mg; *t*_R_ = 74.5 min).

#### (13*E*)-4a,6α,8α-Trihydroxylabd-13(14),17(18)-dien-16,19-olide (**1**):

colorless gum; [α]^25^_D_ −8 (*c* 0.1, CH_3_OH); UV (CH_3_OH) λ_max_ (log ε) 201 (4.52) nm; ECD (CH_3_OH, *c* 0.4 mM, 0.1 cm); Δε −0.48 (209 nm), −0.03 (246 nm); IR (KBr) ν_max_ 3369, 2928, 2858, 1756 (sh), 1738, 1667, 1643, 1458, 1442, 1389, 1300, 1262, 1173 (sh), 1154, 1124, 1095, 1069, 1040, 1023, 983, 933, 888, 847, 736, 702 cm^–1^; ^1^H NMR (CDCl_3_, 600 MHz) and ^13^C NMR (CDCl_3_, 150 MHz), see Table 1; HRESIMS (positive-ion mode) *m*/*z* 429.2605 [M + Na]^+^ (calcd for C_24_H_38_O_5_Na^+^, 429.2611) (error: 1.40 ppm).

#### (4*R*,5*R*,8*R*,9*R*,10*S*,16*R*,13*E*)-8-Hydroxy-23-carboxymethyllabd-13(14),17(18)-dien-16,19-olide (**2**):

colorless, amorphous powder; [α]^25^_D_ −4 (*c* 0.2, CH_3_OH); UV (CH_3_OH) λ_max_ (log ε) 205 (4.37) nm; ECD (CH_3_OH, *c* 0.4 mM, 0.1 cm); Δε −4.97 (209 nm), −0.76 (246 nm); IR (KBr) ν_max_ 3444, 2929, 2868, 1754, 1726, 1645, 1452, 1446, 1388, 1299, 1248, 1170, 1151, 1136, 1081, 1065, 983, 935, 849, 805, 736, 701 cm^–1^; ^1^H NMR (CDCl_3_, 600 MHz) and ^13^C NMR (CDCl_3_, 150 MHz), see Table 1; HRESIMS (positive-ion mode) *m*/*z* 433.2937 [M + H]^+^ (calcd for C_26_H_41_O_5_^+^, 433.2949) (error: 2.77 ppm).

#### (4*R*,5*R*,6*S*,8*R*,9*R*,10*S*,15*S*,16*S*,13*E*)-8,15-Dihydroxy-23-carboxymethyllabd-13(14),17(18)-dien-16,19-olide (**3**):

colorless, amorphous powder; [α]^25^_D_ +2 (*c* 0.1, CH_3_OH); UV (CH_3_OH) λ_max_ (log ε) 208 (3.94) nm; ECD (CH_3_OH, *c* 0.4 mM, 0.1 cm); Δε −0.94 (207 nm), +0.69 (223 nm), −0.58 (244 nm); IR (KBr) ν_max_ 3401, 2923, 2852, 1759 (sh), 1728, 1645, 1456, 1388, 1297, 1249, 1170, 1151, 1115, 1064, 1037, 985, 850, 803, 735, 666 cm^–1^; ^1^H NMR (CDCl_3_, 600 MHz) and ^13^C NMR (CDCl_3_, 125 MHz), see Table 1; HRESIMS (positive-ion mode) *m*/*z* 471.2702 [M + Na]^+^ (calcd for C_26_H_40_O_6_Na^+^, 471.2717) (error: 3.18 ppm).

#### (14*E*)-Methylmanoyloxide-14,16,18-trien-19,16-oxide-23-carboxylate (**4**):

colorless, amorphous powder; [α]^25^_D_ +27 (*c* 0.1, CH_3_OH); UV (CH_3_OH) λ_max_ (log ε) 210 (3.37), 269 (3.48) nm; ECD (CH_3_OH, *c* 0.4 mM, 0.1 cm); Δε +1.09 (221 nm), +1.46 (274 nm); IR (KBr) ν _max_ 3400 (w), 2929, 2867, 2333, 1727, 1661, 1456, 1387, 1246, 1170, 1142, 1093, 1077, 1061, 968, 957, 890, 840, 734, 709 cm^–1^; ^1^H NMR (CDCl_3_, 600 MHz) and ^13^C NMR (CDCl_3_, 150 MHz), see Table 2; HRESIMS (positive-ion mode) *m*/*z* 415.2833 [M + Na]^+^ (calcd for C_26_H_38_O_4_Na^+^, 415.2843) (error: 2.41 ppm).

#### (13*E*)-8α-Hydroxy-23α-*O*-methyl-23,6α-epoxylabd-13(14),17(18)-dien-16,19-olide (**5**):

colorless, amorphous powder; [α]^25^_D_ −4 (*c* 0.2, CH_3_OH); UV (CH_3_OH) λ_max_ (log ε) 205 (4.65) nm; ECD (CH_3_OH, *c* 0.4 mM, 0.1 cm); Δε −10.86 (209 nm), −2.17 (242 nm); IR (KBr) ν_max_ 3436, 2925, 2869, 2310, 1756, 1741, 1668, 1644, 1457, 1443, 1387, 1298, 1266, 1173, 1148, 1101, 1048, 983, 961, 929, 890, 845, 739, 707 cm^–1^; ^1^H NMR (CDCl_3_, 600 MHz) and ^13^C NMR (CDCl_3_, 150 MHz), see Table 2; HRESIMS (positive-ion mode) *m*/*z* 455.2798 [M + Na]^+^ (calcd for C_26_H_40_O_5_Na^+^, 455.2768) (error: −6.59 ppm).

#### (4*R*,5*R*,6*S*,8*R*,9*R*,10*S*,15*S*,16*S*,23*S*,13*E*)-8,15-Dihydroxy-23-*O*-methyl-23,6-epoxylabd-13(14),17(18)-dien-16,19-olide (**6**):

colorless, amorphous powder; [α]^25^_D_ +46 (*c* 0.04, CH_3_OH); UV (CH_3_OH) λ_max_ (log ε) 212 (4.06) nm; ECD (CH_3_OH, *c* 0.4 mM, 0.1 cm); Δε −3.46 (209 nm), +1.00 (225 nm), −1.82 (244 nm); IR (KBr) ν_max_ 3401, 2925, 2862, 2312, 1759, 1738, 1666, 1641, 1458, 1440, 1387, 1303, 1268, 1174, 1152, 1101, 1036, 988, 961, 928, 912, 890, 845, 736, 701, 669 cm^–1^; ^1^H NMR (CDCl_3_, 600 MHz) and ^13^C NMR (CDCl_3_, 150 MHz), see Table 2; HRESIMS (positive-ion mode) *m*/*z* 471.2698 [M + Na]^+^ (calcd for C_26_H_40_O_6_Na^+^, 471.2717) (error: 4.03 ppm).

#### (4*R*,5*R*,6*S*,8*R*,9*R*,10*S*,16*R*,23*S*,13*E*)-8,23-Dihydroxy-23,6-epoxylabd-13(14),17(18)-dien-16,19-olide (**7**):

colorless, amorphous powder; [α]^25^_D_ +14 (*c* 0.4, CH_3_OH); UV (CH_3_OH) λ_max_ (log ε) 209 (4.04) nm; ECD (CH_3_OH, *c* 0.4 mM, 0.1 cm); Δε −8.06 (210 nm), −1.39 (244 nm); IR (KBr) ν _max_ 3430, 3058, 2930, 2869, 2715, 1756 (sh), 1732, 1644, 1455, 1445, 1387, 1301, 1262, 1171, 1151, 1072, 1053, 982, 962, 937, 892, 867, 847, 736, 701 cm^–1^; ^1^H NMR (CDCl_3_, 600 MHz) and ^13^C NMR (CDCl_3_, 150 MHz), see Table 3; HRESIMS (positive-ion mode) *m*/*z* 441.2611 [M + Na]^+^ (calcd for C_25_H_38_O_5_Na^+^, 441.2611) (error: 0.00 ppm).

#### (13*E*)-8α,23-Dihydroxylabd-13(14),17(18)-dien-16,19-olide (**8**):

colorless, amorphous powder; [α]^25^_D_ −10 (*c* 0.2, CH_3_OH); UV (CH_3_OH) λ_max_ (log ε) 201 (4.45) nm; ECD (CH_3_OH, *c* 0.4 mM, 0.1 cm); Δε −7.63 (209 nm), −1.20 (244 nm); IR (KBr) ν_max_ 3411, 2927, 2970, 1756 sh, 1739, 1666, 1644, 1510, 1456, 1442, 1386, 1300, 1264, 1173, 1152, 1122, 1100, 1066, 1047, 983, 938, 885, 848, 737, 703 cm^–1^; ^1^H NMR (CDCl_3_, 600 MHz) and ^13^C NMR (CDCl_3_, 150 MHz), see Table 3; HRESIMS (positive-ion mode) *m*/*z* 427.2823 [M + Na]^+^ (calcd for C_25_H_40_O_4_Na^+^, 427.2819) (error: −0.94 ppm).

#### (13*E*)-Labd-13(14),17(18)-dien-8α,16,19-triol (**9**):

colorless, amorphous powder; [α]^25^_D_ +10 (*c* 0.06, CH_3_OH); UV(CH_3_OH) λ_max_ (log ε) 201 (4.47) nm; ECD (CH_3_OH, *c* 0.4 mM, 0.1 cm); Δε +7.75 (201 nm); IR (CH_2_Cl_2_) ν_max_ 3369, 2925, 2854, 1723 (w), 1663, 1647, 1455, 1387, 1263, 1158, 1125, 1083, 1065, 1046, 1022, 937, 907, 846, 738, 607 cm^–1^; ^1^H NMR (CDCl_3_, 600 MHz) and ^13^C NMR (CDCl_3_, 150 MHz), see Table 3; ESIMS^2^*m*/*z* (rel int) 397 [(M – H_2_O) + Na]^+^ (100), 385 [(M – CH_2_O) + Na]^+^ (18), 369 [(M – HCOOH) + Na]^+^ (12); HRESIMS (positive-ion mode) *m*/*z* 415.3170 [M + Na]^+^ (calcd for C_25_H_44_O_3_Na^+^, 415.3183) (error: 3.13 ppm).

### Preparation of MTPA Esters

To a solution of **3** (5.0 mg, 11 μmol) in dry CH_2_Cl_2_ (400 μL), in a reactive vial, were subsequently added pyridine (4.5 μL, 56 μmol) and (*R*)-(−)-MTPA-Cl (8.3 μL, 45 μmol). The progress of the reaction was monitored by TLC analysis, by eluting with a solvent composed of hexanes and EtOAc in a 1:1 ratio. The mixture was left overnight (no trace of the starting material was present), and the reaction was quenched by addition of 400 μL of distilled water. The water layer was extracted three times with 2.0 mL of Et_2_O. The organic layer was dried with anhydrous MgSO_4_ and concentrated in vacuo. The crude reaction mixture contained the (*S*)-MTPA ester of **3**. The same procedure was repeated in the presence of (*S*)-(+)-MTPA-Cl.

To a solution of **6** (5.0 mg, 11 μmol) in dry CH_2_Cl_2_ (400 μL), in a reactive-vial, were subsequently added pyridine (4.5 μL, 56 μmol) and (*R*)-(−)-MTPA-Cl (8.3 μL, 45 μmol). The progress of the reaction was monitored by TLC analysis, by eluting with a solvent composed of hexanes and EtOAc in a 1:1 ratio. The mixture was left overnight (no trace of the starting material was present), and the reaction was quenched by addition of 400 μL of distilled water. The water layer was extracted three times with 2.0 mL of Et_2_O. The organic layer was dried with anhydrous solid MgSO_4_ and concentrated in vacuo. The crude reaction mixture contained the (*S*)-MTPA ester of **6**. The same procedure was repeated in the presence of (*S*)-(+)-MTPA-Cl.

### Computational Methods

Conformational analysis was performed with Schrödinger MacroModel 9.8 (Schrödinger, LLC, NY, USA) employing the OPLS2005 (optimized potential for liquid simulations) force field in CHCl_3_ for VCD calculations. The five conformers with the lowest energy were selected for geometrical optimization and energy calculation applying DFT with the Becke’s nonlocal three-parameter exchange and correlation functional and the Lee–Yang–Parr correlation functional level (B3LYP), using the 6-31G+(d,p) basis set and the SCRF method with the CPMC model for solvation with the Gaussian 09 program package. Vibrational frequencies (given as wavenumbers in cm^–1^), rotator strength (Rstr), IR intensity (IRinten), and dipole strength (Rstr) were calculated in CHCl_3_ with B3LYP/6-31+G(d,p). VCD curves were obtained on the basis of rotator strengths with a bandwidth of 7 cm^–1^ using CDspecTech v22.0.^83,84^ VCD spectra were calculated from the spectra of individual conformers according to their contribution calculated by Boltzmann weighting. Comparison was done visually as well as by calculation of similarity indices (*Sim*VA, *Sim*VCD), which were generated by VCDspecTech v22.0.^57^ The *Sim*VCD values were plotted against the scaling factors of the *x* axis, and graphs compared between the different stereoisomers.

### Statistical Analysis

All determinations were done in triplicate, and the results reported as mean ± standard deviation (SD). Data were considered statistically significant at *p* ≤ 0.05. The null hypothesis of equality in action for all compounds was tested with one-way ANOVA.^85^ In all cases the null hypothesis was rejected, and the possible differences among formed groups were tested with the Bonferroni method.

### Antimicrobial Activity

A total of 30 strains (27 clinical strains and three isolates of marine origin) previously isolated from different specimens and identified according to standard procedures^86^ and by MALDI TOF Vitek MS Biomérieux were used. All strains were deposited in the collection of the Microbiology Central Laboratory of the San Martino Hospital (Laboratorio di Analisi Chimico-Cliniche e Microbiologia, IRCCS Azienda Ospedaliera Universitaria San Martino IST, Istituto Nazionale per la Ricerca sul Cancro, Largo R. Benzi 10-16132 Genova, Italy) (code of strains indicating the location of the collection: MB). Twenty-four strains belonged to 17 Gram-positive species [*Staphylococcus aureus*, *S. epidermidis*, *S. saprophyticus*, *S. capitis*, *S. warneri*, *S. simulans*, *S. lugdunensis*, *S. hemolyticus*, *S. hominis*, *Streptococcus agalactiae* (MB 149), *S. pneumoniae* (MB 35), *Enterococcus faecium*, *E. faecalis*, *E. avium*, *E. casseliflavus*, *E. durans*, and *E. gallinarum*], four were clinical strains of Gram-negative species [*Escherichia coli* (MB 123), *Proteus mirabilis* (MB 14), *Moraxella catarrhalis* (MB 15), and *Klebsiella pneumoniae* (MB 11)], and two were clinical strains of fungi [*Candida albicans* (MB 31) and *C. glabrata* (MB 8)]. Among the Gram-positive organisms, two *S. aureus* strains were methicillin- and multidrug-resistant (MRSA)^87,88^ (MB 18, MB 188). Two *S. epidermidis* were methicillin- and multidrug-resistant (MRSE) (MB 165, MB 169). *S. saprophyticus* MB41, *S. simulans* MB 94, and *S. lugdunensis* MB 96 were methicillin-susceptible, while *S. capitis* MB 71, *S. warneri* MB 74, *S. hemolyticus* MB 115, and *S. hominis* MB 124 were all methicillin-resistant isolates. One *E. faecalis* was vancomycin-susceptible (MB 76), and three were vancomycin-resistant (VRE) (MB 1, MB 19, MB 51). One *E. faecium* was vancomycin-susceptible (MB 2), and two were VRE (MB 3, MB 152). *E. faecalis* MB 19 and MB 51 and *E. faecium* MB 3 were of marine origin, being isolated from seawater of the Ligurian west coast. *E. avium* MB 119 and *E. durans* MB 113 were vancomycin susceptible, while *E. casseliflavus* and *E. gallinarum* were vancomycin resistant. The preparation of solutions of test compounds and control antibiotics as well as susceptibility testing was performed as previously described.^89^ Minimum inhibitory concentrations (MICs) were determined following the microdilution procedure as reported.^89^

### Purified Bovine Rod OS Preparations

Purified bovine rod outer segments were prepared under dim red light at 4 °C from 14 retinas, by sucrose/Ficoll continuous gradient centrifugation^90^ in the presence of protease inhibitor cocktail (Sigma-Aldrich) and ampicillin (100 μg/mL). OS preparations were characterized for integrity of plasma membrane as reported.^90^

### ATP Synthesis Assay in Rod OS

Rod OS (5 μg)^40^ were incubated for 5 min at 37 °C in 50 mM Tris/HCl (pH 7.4), 5 mM KCl, 1 mM EGTA, 5 mM MgCl_2_, 0.6 mM ouabain, 0.25 mM di(adenosine)-5-pentaphosphate (Ap5A, adenylate kinase inhibitor), 5 mM KH_2_PO_4_, 20 mM succinate, 0.35 mM NADH, and 25 μg/mL ampicillin. ATP synthesis was induced by adding 0.1 mM ADP. Reaction was stopped with 7% perchloric acid. ATP concentration was measured by the luciferin/luciferase chemiluminescent method (Roche Diagnostics Corporation, Indianapolis, IN, USA) in a luminometer (Lumi-Scint, Bioscan Inc., Washington, DC, USA). Where necessary, the incubation medium contained 30 μM resveratrol or 80 μg/mL of different purified *S. tingitana* extracts, semipurified fractions, or pure compounds.

### ATP Hydrolysis Assay in Rod OS

The ATPase activity of rod OS was assayed by the pyruvate kinase/lactate dehydrogenase system, in which hydrolysis of ATP is coupled to the oxidation of NADH followed at 340 nm (ε340 for NADH = 6.22 mM^–1^·cm^–1^), as previously described.^40^ Rod OS (40 μg) were added to a reaction mixture containing 50 mM HEPES, pH 7.4, 100 mM KCl, 150 mM NaCl, 1 mM EGTA, 2.5 mM MgCl_2_, 0.8 mM ouabain, 0.15 mM NADH, 0.4 mM Ap5A (adenylate kinase inhibitor), 1.5 mM phosphoenolpyruvate, pyruvate kinase, and lactate dehydrogenase, and 25 μg/mL ampicillin. ATP hydrolysis was induced by adding 1 mM ATP. Where necessary, the incubation medium contained 80 μg of different purified *S. tingitana* extracts, semipurified fractions, or pure compounds.

### Determination of ATP Concentration in Bacterial Culture in the Presence of Manool (**17**)

Strains of *E. faecalis* MB 1 (VRE) and *E. faecium* MB 152 (VRE) were grown in Mueller Hinton (MH) broth (BD) at 37 °C overnight. The overnight culture was diluted 1:10^6^ in 50 mL of fresh MH broth and incubated at 37 °C for 2 h. Cultures were diluted up to OD_600_ and, when necessary, manool was added at concentrations corresponding to 5 × MICs. Aliquots of samples were collected at two time points (0 and 2 h) to determine ATP concentration using BacTiter-Glo microbial cell viability assay reagent (Promega, Madison, WI, USA). One hundred microliters of culture was mixed with an equal volume of BacTiter-Glo microbial cell viability assay reagent in Eppendorf tubes and incubated at room temperature for 5 min. After incubation, luminescence was read in a luminometer (Lumi-Scint, Bioscan Inc., Washington, DC, USA). ATP standard solutions were prepared using adenosine 5-triphosphate disodium salt hydrate (A2383, Sigma-Aldrich, St. Louis, MO, USA), and a standard curve using ATP standard at concentrations between 1 and 0.001 pmol was recorded. ATP concentrations in bacterial samples were determined by comparison with the ATP standard curve for each assay. MH was included in all assays as the negative control.

### Docking Studies

Manool (**17**) and quercetin were built using Maestro^91^ and subjected to minimization with Macromodel,^92^ employing the generalized Born/surface area model to simulate a water environment. The conjugate gradient algorithm, the MMFFs force field, and a distance-dependent dielectric constant of 1.0 were used for the minimization, performed until a convergence value of 0.05 kcal/(Å·mol) was reached. The ligands were docked into the X-ray structure of bovine F_1_-ATPase in complex with inhibitor quercetin (PDB code 2JJ2)^39^ using AUTODOCK4.2.^93^ AUTODOCK TOOLS^94^ were used to define the torsion angles in the ligand, to add the solvent model, and to assign partial atomic charges (Kollman for the protein and Gasteiger for the ligand). A grid box of 56, 50, and 50 points in the *x*, *y*, and *z* directions, respectively, centered on the cocrystallized inhibitor was used to define the docking site for AUTODOCK calculations. The energetic maps required for docking were generated with a grid spacing of 0.375 Å and a distance-dependent function of the dielectric constant. The ligands were docked using 200 Lamarckian genetic algorithm runs of the AUTODOCK search. During each docking run, 10 000 000 steps of energy evaluation were performed and a maximum of 10 000 000 generations were simulated starting from an initial population of 500 individuals. The final docking solutions were clustered together using an rms cutoff of 2.0 Å and leaving all other settings as their defaults. The clusters of solutions with a population higher than 5%, i.e., including more than 5% of all the generated docking poses, were considered.

### Molecular Dynamic Simulations

All simulations were carried out using AMBER 14^95^ using the X-ray structure of bovine F_1_-ATPase in complex with quercetin (PDB code 2JJ2) already employed for docking. The initial and terminal segments of all protein monomers whose residues were placed more than 30 Å away from the bound ligand were not considered in the simulations. All ligand–protein complexes obtained by docking were solvated with a 15 Å water cap within a parallelepiped water box; chloride ions were then added as counterions for neutralizing the system. General amber force field (GAFF) parameters were assigned to the ligands, while partial charges were calculated using the AM1-BCC method. Initially, the complexes were subjected to energy minimization through 5000 steps of steepest descent followed by conjugate gradient, until a convergence of 0.05 kcal/(mol·Å^2^) was reached. The minimized systems were used as a starting point for an MD simulation protocol composed of three steps. In the first one, 0.5 ns of constant-volume simulation was performed, raising the temperature of the system from 0 to 300 K. In the second step, the system was equilibrated through a 3 ns constant-pressure simulation where the temperature was kept constant at 300 K by using the Langevin thermostat. In the third and last MD step, additional 26.5 ns of constant-pressure simulation was performed, thus reaching a total simulation time of 30 ns. In both the minimization and the three MD steps, a harmonic potential of 10 kcal/(mol·Å^2^) was applied to the protein α carbons. All MD steps were run using particle mesh Ewald electrostatics and periodic boundary conditions,^96^ while a cutoff of 10 Å was employed for the nonbonded interactions and the SHAKE algorithm was used to keep rigid all bonds involving hydrogens.

### Binding Energy Evaluation

Relative binding free energy evaluations were performed using AMBER 14. The trajectories extracted from the last 15 ns of each simulation were used for the calculation, for a total of 150 snapshots (at time intervals of 100 ps). van der Waals, electrostatic, and internal interactions were calculated with the SANDER module of AMBER 14, whereas the Poisson–Boltzman method was employed to estimate polar energies through the MM-PBSA module of AMBER 14 as previously reported.^75,77^ Gas and water phases were represented using dielectric constants of 1 and 80, respectively, while nonpolar energies were calculated with the MOLSURF program. The entropic term was considered as approximately constant in the comparison of the ligand–protein energetic interactions.

## Supporting Information Available

Extraction and isolation of compounds of *S. tingitana*; NMR, UV, ECD, and MS spectra of compounds **1**–**9**; IR spectra of compounds **2**, **3**, **6**, and **7**; VCD spectra of compounds **2**, **3**, and **7**; MIC values of total extract and fractions; effect of total extract, *n*-hexane-insoluble and -soluble fractions, and relative semipurified fractions (I_a_–VI_a_, I_b_–VI_b_) on ATP synthesis and ATPase activity in rod OS; MM-PBSA results for the three different ligand protein complexes of **17** bound to F_1_-ATPase; analysis of the MD simulation of the reference complex (PDB code 2JJ2) (PDF)
