# Supplementary material for: Antibacterial and ATP Synthesis Modulating Compounds from Salvia tingitana
Source: J Nat Prod. 2020 Mar 17;83(4):1027–42. doi: 10.1021/acs.jnatprod.9b01024 (PMC7997632; doi:10.1021/acs.jnatprod.9b01024)
Supplement: Supplementary file 1 — np9b01024_si_001.pdf [file np9b01024_si_001.pdf]

Supporting Information for:

Antibacterial and ATP synthesis modulating compounds from *Salvia tingitana*

Angela Bisio,[*,†] Anna M. Schito,[‡] Francesca Pedrelli,[†] Ombeline Danton,[§] Jakob K. Reinhardt,[§] Giulio Poli,[⊥] Tiziano Tuccinardi,[⊥] Thomas Bürgi,[∥] Francesco De Riccardis, [∇]Mauro Giacomini,[O] Daniela Calzia,[†] Isabella Panfoli,[†] Gian Carlo Schito,[†] Matthias Hamburger,[§] and Nunziatina De Tommasi[♯]

[†]Department of Pharmacy, University of Genova, Viale Cembrano 4, 16148 Genova, Italy
[‡]Department of Integrated Surgical and Diagnostical Sciences, University of Genova, Largo Rosanna Benzi 8, 16145 Genova, Italy
[§]Department of Pharmaceutical Sciences, University of Basel, Klingelbergstrasse 50, 4056 Basel, Switzerland
[⊥]Department of Pharmacy, University of Pisa, via Bonanno 6, 56126 Pisa, Italy
[∥]Department of Chemical Physics, University of Geneva, 30 Quai Ernest-Ansermet, 1211 Genève 4, Switzerland
[∇]Department of Chemistry and Biology, University of Salerno, Via Giovanni Paolo II 132, 84084 Salerno, Italy
[O]Department of Informatics Bioengineering Robotics and System Engineering, University of Genova, Via all'Opera Pia, 13, 16145 Genova, Italy
[♯]Department of Pharmacy, University of Salerno, Via Giovanni Paolo II 132, 84084 Salerno, Italy

CONTENT

Extraction and Isolation of compounds of *S. tingitana*.
Figure S1. ^{1}H NMR (600 MHz, $CDCl_3$) spectrum of compound **1**.
Figure S2. HSQC (600 MHz, $CDCl_3$) spectrum of compound **1**.
Figure S3. HMBC (600 MHz, $CDCl_3$) spectrum of compound **1**.
Figure S4. COSY (600 MHz, $CDCl_3$) spectrum of compound **1**.
Figure S5. 1D TOCSY (600 MHz, $CDCl_3$) spectrum of compound **1**.
Figure S6. NOESY (600 MHz, $CDCl_3$) spectrum of compound **1**.
Figure S7. HRESIMS spectrum of compound **1**.
Figure S8. ^{1}H NMR (600 MHz, $CDCl_3$) spectrum of compound **2**.
Figure S9. ^{13}C NMR (125 MHz, $CDCl_3$) spectrum of compound **2**.
Figure S10. HSQC (600 MHz, $CDCl_3$) spectrum of compound **2**.
Figure S11. HMBC (600 MHz, $CDCl_3$) spectrum of compound **2**.
Figure S12. COSY (600 MHz, $CDCl_3$) spectrum of compound **2**.
Figure S13. NOESY (600 MHz, $CDCl_3$) spectrum of compound **2**.
Figure S14. Comparison of experimental and computed IR spectra ($CDCl_3$) for compound **2**.
Figure S15. Comparison of experimental and computed VCD spectra in $CDCl_3$ for compound **2**.
Figure S16. HRESIMS spectrum of compound **2**.
Figure S17. ^{1}H NMR (600 MHz, $CDCl_3$) spectrum of compound **3**.
Figure S18. HSQC (600 MHz, $CDCl_3$) spectrum of compound **3**.
Figure S19. HMBC (600 MHz, $CDCl_3$) spectrum of compound **3**.
Figure S20. COSY (600 MHz, $CDCl_3$) spectrum of compound **3**.
Figure S21. NOESY (500 MHz, $CDCl_3$) spectrum of compound **3**.
Figure S22. Comparison of experimental and computed IR spectra ($CDCl_3$) for compound **3**.
Figure S23. Comparison of experimental and computed VCD spectra in $CDCl_3$ for compound **3**.
Figure S24. ^{1}H NMR (400 MHz, $CDCl_3$) spectrum of S-MTPA ester of compound **3** (by esterification of **3** with (R)-(-)-MTPA-Cl).
Figure S25. ^{1}H NMR (400 MHz, $CDCl_3$) spectrum of R-MTPA ester of compound **3** (by esterification of **3** with (S)-(+)-MTPA-Cl).
Figure S26. HRESIMS spectrum of compound **3**.

Figure S27. ^{1}H NMR (600 MHz, $CDCl_3$) spectrum of compound **4**.
Figure S28. ^{13}C NMR (125 MHz, $CDCl_3$) spectrum of compound **4**.
Figure S29. HSQC (600 MHz, $CDCl_3$) spectrum of compound **4**.
Figure S30. HMBC (600 MHz, $CDCl_3$) spectrum of compound **4**.
Figure S31. COSY (600 MHz, $CDCl_3$) spectrum of compound **4**.
Figure S32. NOESY (500 MHz, $CDCl_3$) spectrum of compound **4**.
Figure S33. HRESIMS spectrum of compound **4**.
Figure S34. ^{1}H NMR (600 MHz, $CDCl_3$) spectrum of compound **5**.
Figure S35. ^{13}C NMR (125 MHz, $CDCl_3$) spectrum of compound **5**.
Figure S36. HSQC (600 MHz, $CDCl_3$) spectrum of compound **5**.
Figure S37. HMBC (600 MHz, $CDCl_3$) spectrum of compound **5**.
Figure S38. COSY (600 MHz, $CDCl_3$) spectrum of compound **5**.
Figure S39. NOESY (500 MHz, $CDCl_3$) spectrum of compound **5**.
Figure S40. HRESIMS spectrum of compound **5**.
Figure S41. ^{1}H NMR (600 MHz, $CDCl_3$) spectrum of compound **6**.
Figure S42. HSQC (600 MHz, $CDCl_3$) spectrum of compound **6**.
Figure S43. HMBC (600 MHz, $CDCl_3$) spectrum of compound **6**.
Figure S44. COSY (600 MHz, $CDCl_3$) spectrum of compound **6**.
Figure S45. NOESY (500 MHz, $CDCl_3$) spectrum of compound **6**.
Figure S46. Comparison of experimental and computed IR spectra ($CDCl_3$) for compound **6.**
Figure S47. Comparison of experimental and computed VCD spectra in $CDCl_3$ for compound **6**
Figure S48. ^{1}H NMR (400 MHz, $CDCl_3$) spectrum of S-MTPA ester of compound **6** (by esterification of **6** with (R)-(-)-MTPA-Cl).
Figure S49. ^{1}H NMR (400 MHz, $CDCl_3$) spectrum of R-MTPA ester of compound **6** (by esterification of **6** with (S)-(+)-MTPA-Cl).
Figure S50. HRESIMS spectrum of compound **6**.
Figure S51. ^{1}H NMR (600 MHz, $CDCl_3$) spectrum of compound **7**.
Figure S52. HSQC (600 MHz, $CDCl_3$) spectrum of compound **7**.
Figure S53. HMBC (600 MHz, $CDCl_3$) spectrum of compound **7**.
Figure S54. ROESY (600 MHz, $CDCl_3$) spectrum of compound **7**.
Figure S55. Comparison of experimental and computed IR spectra ($CDCl_3$) for compound **7**.
Figure S56. Comparison of experimental and computed VCD spectra in $CDCl_3$ for compound **7**.
Figure S57. HRESIMS spectrum of compound **7**.
Figure S58. ^{1}H NMR (600 MHz, $CDCl_3$) spectrum of compound **8**.
Figure S59. ^{13}C NMR (125 MHz, $CDCl_3$) spectrum of compound **8**.
Figure S60. HSQC (600 MHz, $CDCl_3$) spectrum of compound **8**.
Figure S61. HMBC (600 MHz, $CDCl_3$) spectrum of compound **8**.
Figure S62. COSY (600 MHz, $CDCl_3$) spectrum of compound **8**.
Figure S63. NOESY (500 MHz, $CDCl_3$) spectrum of compound **8**.
Figure S64. Comparison of experimental and computed UV (A) and ECD (B) (CH_3OH) spectra for compound **8**.
Figure S65. HRESIMS spectrum of compound **8**.
Figure S66. ^{1}H NMR (600 MHz, $CDCl_3$) spectrum of compound **9**.
Figure S67. HSQC (600 MHz, $CDCl_3$) spectrum of compound **9**.
Figure S68. HMBC (600 MHz, $CDCl_3$) spectrum of compound **9**.
Figure S69. COSY (600 MHz, $CDCl_3$) spectrum of compound **9**.
Figure S70. 1D TOCSY (600 MHz, $CDCl_3$) spectrum of compound **9**.
Figure S71. NOESY (500 MHz, $CDCl_3$) spectrum of compound **9**.
Figure S72. Comparison of experimental and computed UV (A) and ECD (B) spectra (CH_3OH) for compound **9**.
Figure S73. HRESIMS spectrum of compound **9**.
Figure S74. Effect of sclareol (**15**) on viable cell number of selected susceptible *Staphylococcus* strains.

Figure S75. Effect of total extract, *n*-hexane insoluble and soluble fractions and relative main fractions (I_a-VI_a, I_b-VI_b) on ATP synthesis in rod Outer Segments (OS).
Figure S76. Effect of compounds isolated from *S. tingitana* on ATP synthesis in rod Outer Segments (OS).
Figure S77. Effect of total extract, *n*-hexane insoluble and soluble fractions and relative main fractions (I_a-VI_a, I_b-VI_b) on ATP hydrolysis activity in rod Outer Segments (OS).
Figure S78. Effect of compounds on isolated from *S. tingitana* on ATP hydrolysis activity in rod Outer Segments (OS).
Figure S79. MD analysis of the three binding modes predicted by docking manool (**17**) into F1-ATPase.
Figure S80. MD analysis of the two binding modes predicted by self-docking quercetin into the co-crystal structure of F1-ATPase.
Figure S81. A) X-ray structure of F1-ATPase bound to quercetin (PDB code 2JJ2) and B) minimized average structure of F1-ATPase in complex with quercetin in binding mode 2.

Table S1. MIC values of total extract *n*-hexane insoluble and soluble fractions and relative main fractions against representative clinical strains.
Table S2. MM-PBSA results for the three different ligand protein complexes of manool (**17**) bound to F_1-ATPase.
Table S3. MM-PBSA results for the two different ligand protein complexes of quercetin bound to F_1-ATPase.

Extraction and Isolation of compounds of *S. tingitana*. Fresh aerial parts (10.3 kg) of *S. tingitana* were immersed in CH_2Cl_2 for 20 s to afford 103.0 g of exudate. The exudate was partitioned with *n*-hexane to afford a *n*-hexane-soluble (85.8 g) and a *n*-hexane-insoluble portion (17.7 g).
The *n*-hexane-insoluble portion was chromatographed in aliquots of 1.0 g on Sephadex LH-20 (53 x 2.5 cm; mobile phase $CHCl_3$-CH_3OH 7:3, 0.24 L; monitoring by TLC) to afford six main fractions (FI_a-FVI_a): FI_a (0.2 g; from 0.00 to 0.12 L) with waxy compounds, FII_a (1.4 g; from 0.12 to 0.14 L), $FIII_a$ (8.6 g; from 0.14 to 0.18 L), FIV_a (3.9 g; from 0.18 to 0.20 L), FV_a (1.6 g; from 0.20 to 0.22 L), and FVI_a (0.6 g; from 0.22 to 0.24 L).
FII_a was separated by CC on silica gel (MPLC; monitoring by TLC) with a mixture of *n*-hexane-$CHCl_3$ (4:1, 0.36 L) and mixtures of $CHCl_3$-CH_3OH (1:0, 0.96 L; 9:1, 0.30 L; 1:1, 0.36 L; 0:1, 0.42 L) into thirteen fractions ($F1_{ai}$-$F13_{ai}$). $F9_{ai}$ (139.0 mg; eluted with $CHCl_3$-CH_3OH 1:1, from 1.14 to 1.70 L) was purified by semi-preparative HPLC to obtain **3** (1.6 mg; t_R = 66.5 min) and **10** (4.7 mg; t_R = 68.0 min).
$FIII_a$ was separated by CC on silica gel (MPLC; monitoring by TLC) with a mixture of *n*-hexane-$CHCl_3$ (4:1, 0.42 L) and mixtures of $CHCl_3$-CH_3OH (1:0, 0.24 L; 1:1, 0.48 L; 0:1, 0.42 L) into twenty-five fractions ($F1_{aii}$-$F25_{aii}$). $F9_{aii}$ (306.2 mg; eluted with $CHCl_3$-CH_3OH 1:0, from 0.60 to 0.75 L) was purified by semi-preparative HPLC to obtain **2** (20.7 mg; t_R = 71.0 min), **22** (4.5 mg; t_R = 67.5 min), **23** (5.4 mg; t_R = 70.5 min), and **24** (4.0 mg; t_R = 64.5 min). $F10_{aii}$ (498.9 mg; eluted with $CHCl_3$-CH_3OH 1:0, from 0.75 to 0.84 L) was purified by semi-preparative HPLC to obtain **2** (3.9 mg; t_R = 71.0 min), **5** (4.7 mg; t_R = 69.0 min), **11** (14.1 mg; t_R = 61.0 min), and **24** (5.8 mg; t_R = 64.5 min). $F12_{aii}$ (263.0 mg; eluted with $CHCl_3$-CH_3OH 1:0, from 1.05 to 1.20 L) was purified by semi-preparative HPLC to obtain **5** (21.8 mg; t_R = 69.0 min) and **11** (25.3 mg; t_R = 61.0 min). $F13_{aii}$ (97.3 mg; eluted with $CHCl_3$-CH_3OH 1:0, from 1.20 to 1.32 L) was purified by semi-preparative HPLC to obtain **5** (10.4 mg; t_R = 69.0 min) and **11** (8.3 mg t_R = 61.0 min). Fraction 14_{aii} (81.1 mg; eluted with $CHCl_3$-CH_3OH 9:1, from 1.32 to 1.50 L) was purified by semi-preparative HPLC to obtain **5** (1.4 mg; t_R = 69.0 min). $F15_{aii}$ (87.7 mg; eluted with $CHCl_3$-CH_3OH 9:1, from 1.50 to 1.53 L) was purified by semi-preparative HPLC to obtain **5** (5.4 mg; t_R = 69.0 min). $F17_{aii}$ (892.3 mg; eluted with $CHCl_3$-CH_3OH 9:1, from 1.56 to 1.59 L) was purified by semi-preparative HPLC to obtain **3** (7.2 mg; t_R = 66.5 min), **6** (35.2 mg; t_R = 66.0 min), and **12** (3.5 mg; t_R = 60.0 min). $F18_{aii}$ (675.0 mg; eluted with $CHCl_3$-CH_3OH 9:1, from 1.59 to 1.62 L) was purified by semi-preparative HPLC to obtain **9** (4.6 mg; t_R = 78.0 min) and **10** (6.6 mg; t_R = 68.0 min). $F19_{aii}$ (1502.7 mg; eluted with $CHCl_3$-CH_3OH 9:1, from 1.62 to 1.68 L) was purified by semi-preparative HPLC to obtain **1** (2.1 mg; t_R = 64.0 min), **7** (16.4 mg; t_R = 65.0 min), **10** (19.1 mg t_R = 68.0 min), **12** (4.0 mg; t_R = 60.0 min), **13** (4.3 mg; t_R = 67.0 min), and **14** (23.0 mg; t_R = 61.5

min). Fraction 20_{aii} (146.2 mg; eluted with $CHCl_3$-CH_3OH 1:0, from 1.68 to 1.71 L) was purified by semi-preparative HPLC to obtain **14** (61.8 mg; t_R = 61.5 min).

FIV_a was separated by CC on silica gel (MPLC; monitoring by TLC) with a mixture of *n*-hexane-$CHCl_3$ (4:1, 0.39 L) and mixtures of $CHCl_3$-CH_3OH (1:0, 0.78 L; 95:5, 0.33 L; 0:1, 0.21 L) into fifteen fractions ($F1_{aiii}$-$F15_{aiii}$). $F7_{aiii}$ (33.3 mg; eluted with $CHCl_3$-CH_3OH 1:0, from 0.87 to 0.96 L) was purified by semi-preparative HPLC to obtain **15** (1.9 mg; t_R = 75.0 min). $F8_{aiii}$ (43.1 mg; eluted with $CHCl_3$-CH_3OH 1:0, from 0.96 to 1.17 L) was purified by semi-preparative HPLC to obtain **15** (4.2 mg; t_R = 75.0 min). $F9_{aiii}$ (333.1 mg; eluted with $CHCl_3$-CH_3OH 95:5, from 0.96 to 1.17 L) was purified by semi-preparative HPLC to obtain **15** (14.9 mg; t_R = 75.0 min) and **16** (4.2 mg; t_R = 74.0 min). $F10_{aiii}$ (141.4 mg; eluted with $CHCl_3$-CH_3OH 95:5, from 1.17 to 1.23 L) was purified by semi-preparative HPLC to obtain **3** (2.6 mg; t_R = 66.5 min), **16** (7.1 mg; t_R = 74.0 min), and **25** (4.6 mg; t_R = 60.5 min). $F11_{aiii}$ (55.6 mg; eluted with $CHCl_3$-CH_3OH 95:5, from 1.23 to 1.26 L) was purified by semi-preparative HPLC to obtain **6** (2.0 mg; t_R = 66.0 min) and **8** (2.1 mg; t_R = 70.0 min). $F13_{aiii}$ (92.1 mg; eluted with $CHCl_3$-CH_3OH 95:5, from 1.29 to 1.38 L) was purified by semi-preparative HPLC to obtain **14** (1.0 mg; t_R = 61.5 min) and **16** (1.0 mg; t_R = 74.0 min). $F14_{aiii}$ (48.0 mg; eluted with $CHCl_3$-CH_3OH 95:5, from 1.38 to 1.50 L) was purified by semi-preparative HPLC to obtain **14** (7.1 mg; t_R = 61.5 min).

FV_a was separated by CC on silica gel (MPLC; monitoring by TLC) with *n*-hexane (0.54 L) and mixtures of $CHCl_3$-CH_3OH (1:0, 0.51 L; 95:5, 0.42 L; 0:1, 0.39 L) into thirteen fractions ($F1_{aiv}$-$F13_{aiv}$). $F10_{aiv}$ (174.1 mg; eluted with $CHCl_3$-CH_3OH 95:5, from 1.35 to 1.41 L) was purified by semi-preparative HPLC to obtain **23** (13.3 mg; t_R = 70.5 min).

The *n*-hexane-soluble portion was chromatographed in aliquots of 1.0 g on Sephadex LH-20 (53 x 2.5 cm; $CHCl_3$-CH_3OH 7:3 as eluent, 0.26 L; monitoring by TLC) to afford seven main fractions (FI_b-$FVII_b$): FI_b (3.1 g; from 0.00 to 0.12 L) with waxy compounds, FII_b (5.6 g; from 0.12 to 0.14 L), $FIII_b$ (14.7 g; from 0.14 to 0.16 L), FIV_b (31.4 g; from 0.16 to 0.18 L), FV_b (16.1 g; from 0.18 to 0.20 L), FVI_b (3.2 g; from 0.20 to 0.22 L), and $FVII_b$ (0.8 g; from 0.22 to 0.24 L).

FII_b was separated by CC on silica gel (MPLC; monitoring by TLC) with mixtures of *n*-hexane-$CHCl_3$ (1:0, 1.23 L; 3:2, 0.27 L) and mixtures of $CHCl_3$-CH_3OH (1:0, 1.17 L; 95:5, 0.15 L; 0:1, 0.42 L) into twenty-three subfractions ($F1_{bi}$-$F23_{bi}$). $F18_{bi}$ (763.6 mg; eluted with $CHCl_3$-CH_3OH 1:0, from 2.52 to 2.55 L) was separated by CC on silica gel (MPLC; monitoring by TLC) with *n*-hexane (0.48 L) and mixtures of $CHCl_3$-CH_3OH (1:0, 0.68 L; 95:5, 0.33 L; 0:1, 0.15 L) into ten fractions ($F1_{bii}$-$F10_{bii}$). $F5_{bii}$ (73.0 mg; eluted with $CHCl_3$-CH_3OH 1:0, from 0.78 to 0.90 L) was purified by semi-preparative HPLC to obtain **2** (6.6 mg; t_R = 71.0 min) and **11** (2.1 mg; t_R = 61.0 min). $F8_{bii}$ (193.0 mg; eluted with $CHCl_3$-CH_3OH 95:5, from 1.32 to 1.44 L) was purified by semi-preparative HPLC to obtain **3** (3.1mg; t_R = 66.5 min), **8** (2.8 mg; t_R = 70.0 min), and **11** (9.3 mg; t_R = 61.0 min). $F19_{bi}$ (1136.4 mg; eluted with $CHCl_3$-CH_3OH 1:0, from 2.55 to 2.58 L) was separated by CC on silica gel (MPLC; monitoring by TLC) with *n*-hexane (0.42 L) and mixtures of $CHCl_3$-CH_3OH (1:0, 0.78 L; 95:5, 0.42 L; 0:1, 0.09 L) into ten fractions ($F1_{biii}$-$F10_{biii}$). $F8_{biii}$ (321.6 mg; eluted with $CHCl_3$-CH_3OH 1:0, from 1.23 to 1.50 L) was purified by semi-preparative HPLC to obtain **3** (2.3 mg; t_R = 66.5 min), **10** (15.5 mg; t_R = 68.0 min), and **11** (12.7 mg; t_R = 61.0 min).

$FIII_b$ was separated by CC on silica gel (MPLC; monitoring by TLC) with a mixture of *n*-hexane-$CHCl_3$ (3:2, 0.63 L) and mixtures of $CHCl_3$-CH_3OH (1:0, 1.14 L; 95:5, 0.15 L; 0:1, 0.66 L) into twenty-four fractions ($F1_{biv}$-$F24_{biv}$). $F11_{biv}$ (611.7 mg; eluted with $CHCl_3$-CH_3OH 1:0, from 0.78 to 0.84 L) was separated by CC on silica gel (MPLC; monitoring by TLC) with *n*-hexane (0.39 L) and mixtures of $CHCl_3$-CH_3OH (1:0, 0.63 L; 95:5, 0.60 L; 0:1, 0.09 L) into fourteen fractions ($F1_{bv}$-$F14_{bv}$). $F6_{bv}$ (137.7 mg; eluted with $CHCl_3$-CH_3OH 1:0, from 0.78 to 0.90 L) was purified by semi-preparative HPLC to obtain was purified by semi-preparative HPLC to obtain **4** (4.6 mg; t_R = 84.0 min). $F12_{biv}$ (eluted with $CHCl_3$-CH_3OH 1:0, from 0.84 to 0.87 L) was purified by semi-preparative HPLC to obtain **4** (9.1 mg; t_R = 84.0 min). $F14_{biv}$ (504.2 mg; eluted with $CHCl_3$-CH_3OH 1:0, from 0.99 to 1.05 L) was purified by semi-preparative HPLC to obtain **23** (3.6 mg; t_R = 70.5 min). $F16_{biv}$ (484.4 mg; eluted with $CHCl_3$-CH_3OH 1:0, from 0.99 to 1.05 L) was purified by semi-preparative HPLC to obtain **22** (1.6 mg; t_R = 67.5 min) and **23** (3.8 mg; t_R = 70.5 min). $F17_{biv}$ (1502.6 mg; eluted with $CHCl_3$-CH_3OH 1:0, from 1.17 to 1.32 L) was purified by semi-preparative HPLC to obtain **2** (27.4 mg; t_R = 71.0 min). $F20_{biv}$ (742.5 mg; eluted with $CHCl_3$-CH_3OH 1:0, from 1.56 to 1.77 L) was purified by semi-preparative HPLC to obtain **15** (168.5 mg; t_R = 75.0 min). $F21_{biv}$ (135.3 mg; eluted with $CHCl_3$-CH_3OH 95:5, from 1.77 to 1.83 L) was purified by semi-preparative HPLC to obtain **15** (32.8 mg; t_R = 75.0 min).

FIV$_b$ was separated by CC on silica gel (MPLC; monitoring by TLC) with a mixture of *n*-hexane-$CHCl_3$ (3:2, 0.75 L) and mixtures of $CHCl_3$-CH_3OH (1:0, 1.08 L; 95:5, 0.30 L; 0:1, 0.42 L) into eighteen fractions (F1$_{bvi}$-F18$_{bvi}$). F3$_{bvi}$ (1.8 g; eluted with *n*-hexane-$CHCl_3$ 6:4, from 0.15 to 0.18 L) was purified by semi-preparative HPLC to obtain **17** (5.6 mg; t_R = 82.0 min). F5$_{bvi}$ (10.53 g; eluted with *n*-hexane-$CHCl_3$ 6:4, from 0.21 to 0.51 L) was separated by CC on silica gel (MPLC; monitoring by TLC) with *n*-hexane (0.45 L) and mixtures of $CHCl_3$-CH_3OH (1:0, 0.84 L; 95:5, 0.36 L; 0:1, 0.06 L) into six fractions (F1$_{bvii}$-F6$_{bvii}$). F3 $_{bvii}$ (780.5 mg; eluted with $CHCl_3$-CH_3OH 1:0, from 0.51 to 0.63 L) was purified by semi-preparative HPLC to obtain **15** (254.6 mg; t_R = 75.0 min) and **23** (7.6 mg; t_R = 70.5 min). F4 $_{bvii}$ (5.11 g; eluted with $CHCl_3$-CH_3OH 1:0, from 0.63 to 0.78 L) was separated by CC on silica gel (MPLC; monitoring by TLC) with *n*-hexane (0.45 L) and mixtures of $CHCl_3$-CH_3OH (1:0, 0.84 L; 95:5, 0.36 L; 0:1, 0.06 L) into twenty-three fractions (1$_{bviii}$-23$_{bviii}$). F13$_{bviii}$ (318.4 mg; eluted with $CHCl_3$-CH_3OH 1:0, from 0.96 to 1.05 L) was purified by semi-preparative HPLC to obtain **23** (5.0 mg; t_R = 70.5 min). F14$_{bviii}$ (770.9 mg; eluted with $CHCl_3$-CH_3OH 1:0, from 0.96 to 1.05 L) was purified by semi-preparative HPLC to obtain **15** (258.1 mg; t_R = 75.0 min). F15$_{bviii}$ (741.4 mg; eluted with $CHCl_3$-CH_3OH 95:5, from 1.05 to 1.17 L) was purified by semi-preparative HPLC to obtain **15** (292.3 mg; t_R = 75.0 min). F17$_{bviii}$ (635.2 mg; eluted with $CHCl_3$-CH_3OH 95:5, from 1.29 to 1.32 L) was purified by semi-preparative HPLC to obtain **15** (67.0 mg; t_R = 75.0 min). F6$_{bvi}$ (2.24 g; eluted with *n*-hexane-$CHCl_3$ 6:4, from 0.51 to 0.75 L) was separated by CC on silica gel (MPLC; monitoring by TLC) with *n*-hexane (1.00 L) and mixtures of $CHCl_3$-CH_3OH (1:0, 1.05 L; 95:5, 0.60 L; 0:1, 0.06 L) into ten fractions (F1$_{bix}$-F10$_{bix}$). F5$_{bix}$ (270.4 mg; eluted with $CHCl_3$-CH_3OH 95:5, from 1.26 to 1.29 L) was purified by semi-preparative HPLC to obtain **15** (233.5 mg; t_R = 75.0 min). F7$_{bvi}$ (3.17 g; eluted with $CHCl_3$-CH_3OH 1:0, from 0.75 to 0.84 L) was separated by CC on silica gel (MPLC; monitoring by TLC) with *n*-hexane (0.42 L) and mixtures of $CHCl_3$-CH_3OH (1:0, 0.54 L; 95:5, 0.75 L) into eighteen fractions (F1$_{bx}$-F18$_{bx}$). F14 $_{bx}$ (188.7 mg) eluted with $CHCl_3$-CH_3OH 95:5, from 1.26 to 1.29 L) was purified by semi-preparative HPLC to obtain **15** (148.0 mg; t_R = 75.0 min). F12$_{bvi}$ (1.31 g; eluted with $CHCl_3$-CH_3OH 95:5, from 1.83 to 1.86 L) was separated by CC on silica gel (MPLC; monitoring by TLC) with *n*-hexane (0.45 L) and mixtures of $CHCl_3$-CH_3OH (1:0, 0.54 L; 95:5, 0.72 L) into fourteen fractions (F1$_{bxi}$-F14$_{bxi}$). F11$_{bxi}$ (1.31 g; eluted with $CHCl_3$-CH_3OH 95:5, from 1.35 to 1.47 L) was purified by semi-preparative HPLC to obtain **8** (7.1 mg; t_R = 70.0 min), and **10** (3.3 mg; t_R = 68.0 min). F13$_{bvi}$ (342.6 mg; eluted with $CHCl_3$-CH_3OH 95:5, from 1.86 to 1.89 L) was purified by semi-preparative HPLC to obtain **1** (1.7 mg; t_R = 64.0 min), **8** (1.5 mg; t_R = 70.0 min), **10** (2.6 mg; t_R = 68.0 min), **12** (1.7 mg; t_R = 60.0 min), **13** (6.0 mg; t_R = 67.0 min), and **14** (10.8 mg; t_R = 61.5 min). F14$_{bvi}$ (296.7 mg; eluted with $CHCl_3$-CH_3OH 95:5, from 1.89 to 1.92 L) was purified by semi-preparative HPLC to obtain **11** (2.7 mg; t_R = 61.0 min), **12** (1.5 mg; t_R = 60.0 min) and **14** (18.3 mg; t_R = 61.5 min). F17$_{bvi}$ (54.5 mg; eluted with $CHCl_3$-CH_3OH 95:5, from 2.13 to 2.19 L) was purified by semi-preparative HPLC to obtain **11** (1.2 mg; t_R = 61.0 min).

FV$_b$ was separated by CC on silica gel (MPLC; monitoring by TLC) with a *n*-hexane (0.39 L) and mixtures of $CHCl_3$-CH_3OH (1:0, 0.84 L; 95:5, 0.39 L; 0:1, 0.06 L) into sixteen fractions (F1$_{bxii}$-F16$_{bxii}$). F3$_{bxii}$ (2.5 g; eluted with $CHCl_3$-CH_3OH 1:0, from 0.39 to 0.54 L) was separated by CC on silica gel (MPLC; monitoring by TLC) with a *n*-hexane (0.39 L) and mixtures of $CHCl_3$-CH_3OH (1:0, 0.54 L; 95:5, 0.72 L; 0:1, 0.06 L) into fourteen fractions (F1$_{bxiii}$-F14$_{bxiii}$). F5$_{bxiii}$ (116.3 mg; eluted with $CHCl_3$-CH_3OH 1:0, from 0.66 to 0.75 L) was purified by semi-preparative HPLC to obtain **15** (10.1 mg; t_R = 75.0 min), **17** (17.3 mg; t_R = 82.0 min) and **18** (4.9 mg; t_R = 62.0 min). F6$_{bxiii}$ (177.9 mg; eluted with $CHCl_3$-CH_3OH 1:0, from 0.75 to 0.89 L) was purified by semi-preparative HPLC to obtain **17** (17.3 mg; t_R = 82.0 min) and **19** (1.6 mg; t_R = 81.0 min). F9$_{bxiii}$ (249.9 mg; eluted with $CHCl_3$-CH_3OH 95:5, from 0.93 to 0.99 L) was purified by semi-preparative HPLC to obtain **21** (3.0 mg; t_R = 76.0 min). F11$_{bxii}$ (355.1 mg; eluted with $CHCl_3$-CH_3OH 95:5, from 1.32 to 1.35 L) was purified by semi-preparative HPLC to obtain **15** (10.0 mg; t_R = 75.0 min). F13$_{bxii}$ (237.7 mg; eluted with $CHCl_3$-CH_3OH 95:5, from 1.41 to 1.47 L) was purified by semi-preparative HPLC to obtain **13** (2.8 mg; t_R = 67.0 min) and **16** (2.1 mg; t_R = 74.0 min).

FVI$_b$ was separated by CC on silica gel (MPLC; monitoring by TLC) with a *n*-hexane (0.24 L) and mixtures of $CHCl_3$-CH_3OH (1:0, 0.93 L; 95:5, 0.45 L; 0:1, 0.07 L) into twenty fractions (F1$_{bxiv}$-F20$_{bxiv}$). F13$_{bxiv}$ (74.5 mg; eluted with $CHCl_3$-CH_3OH 1:0, from 1.02 to 1.17 L) was purified by semi-preparative HPLC to obtain **20** (2.7 mg; t_R = 74.5 min). F17$_{bxiv}$ (87.1 mg; eluted with $CHCl_3$-CH_3OH 1:0, from 1.32 to 1.41 L) was purified by semi-preparative HPLC to obtain **13** (1.1 mg; t_R = 67.0 min).

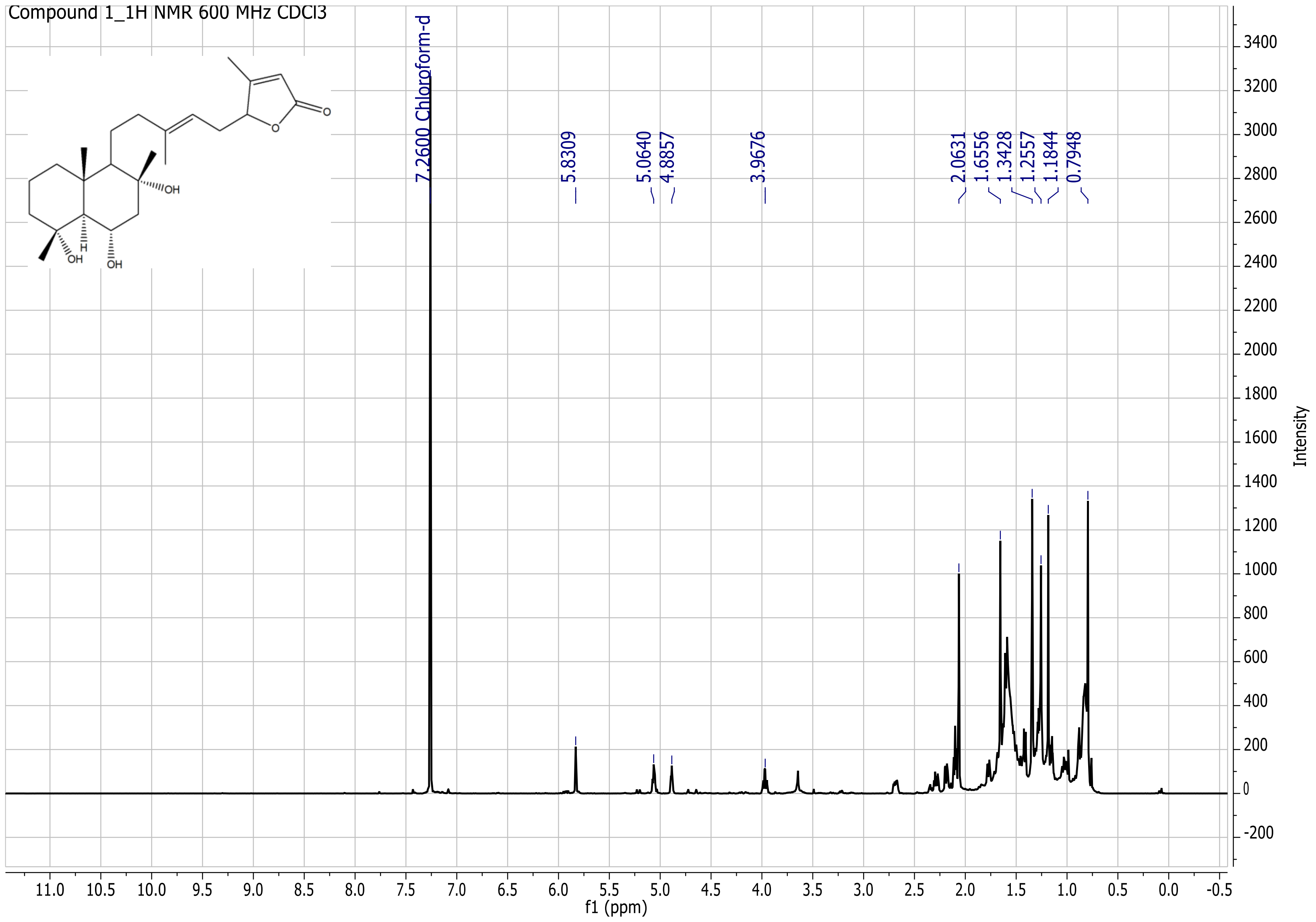

Figure S1. ^{1}H NMR (600 MHz, $CDCl_3$) spectrum of compound **1**.

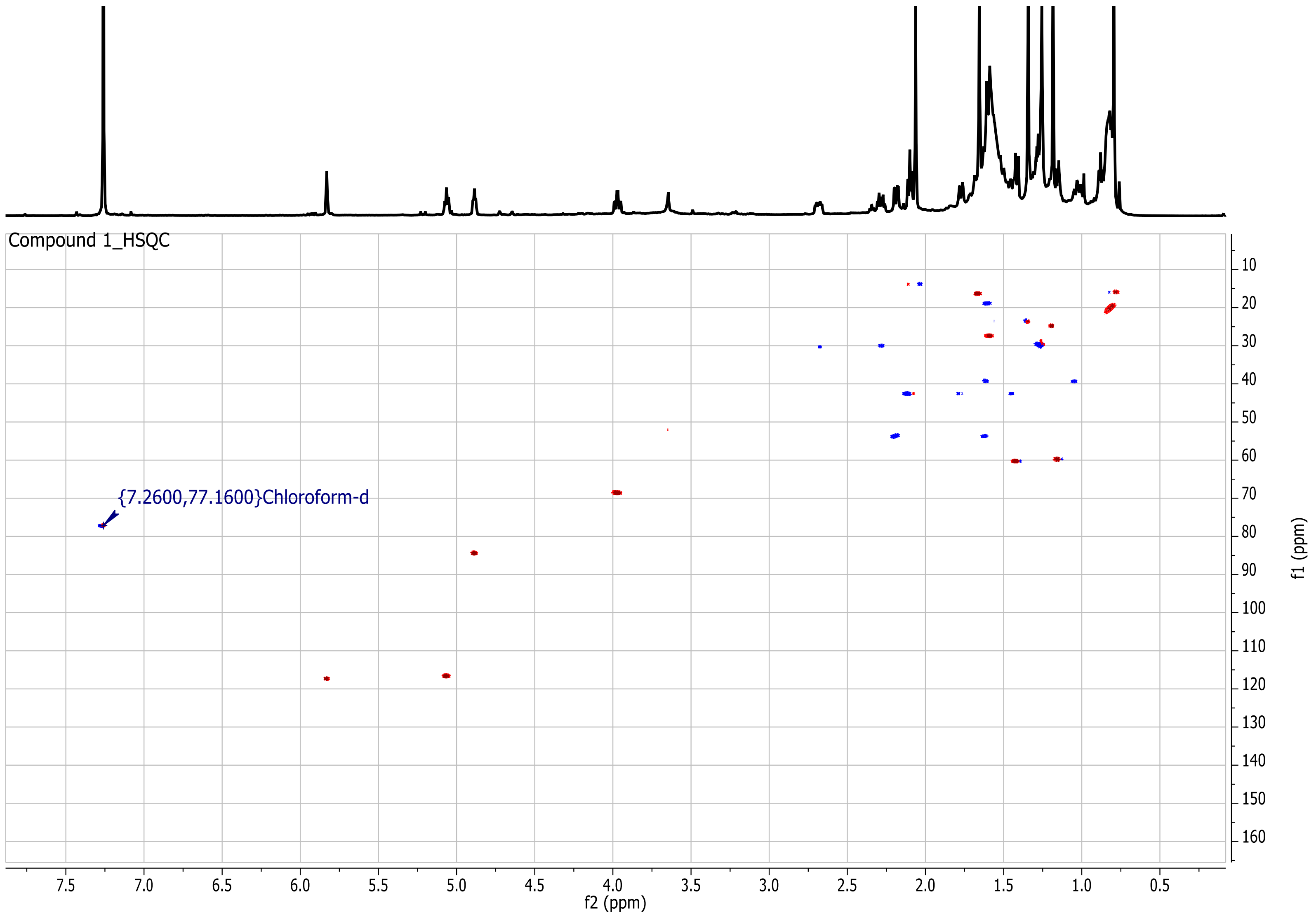

Figure S2. HSQC (600 MHz, $CDCl_3$) spectrum of compound **1**.

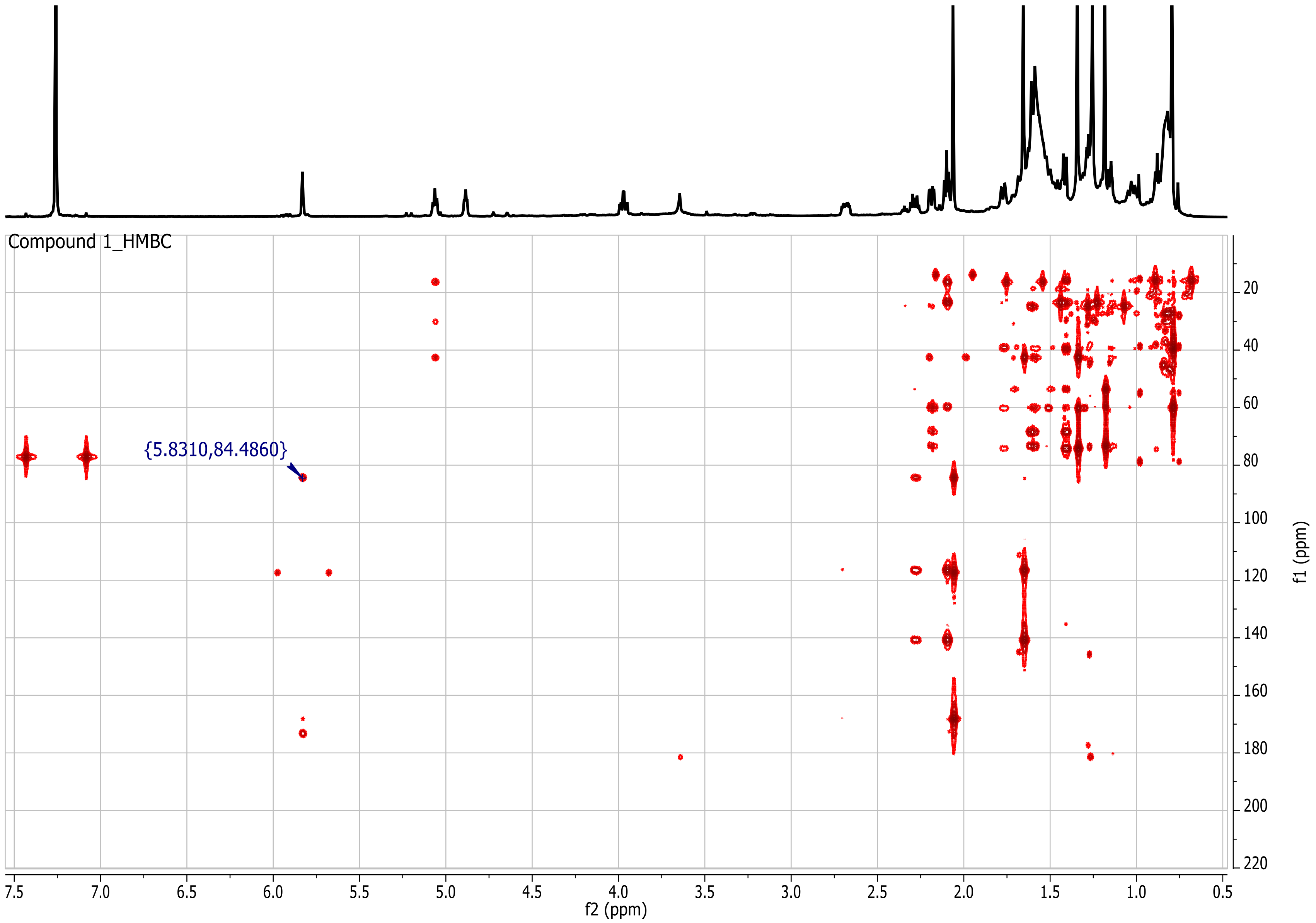

Figure S3. HMBC (600 MHz, $CDCl_3$) spectrum of compound **1**.

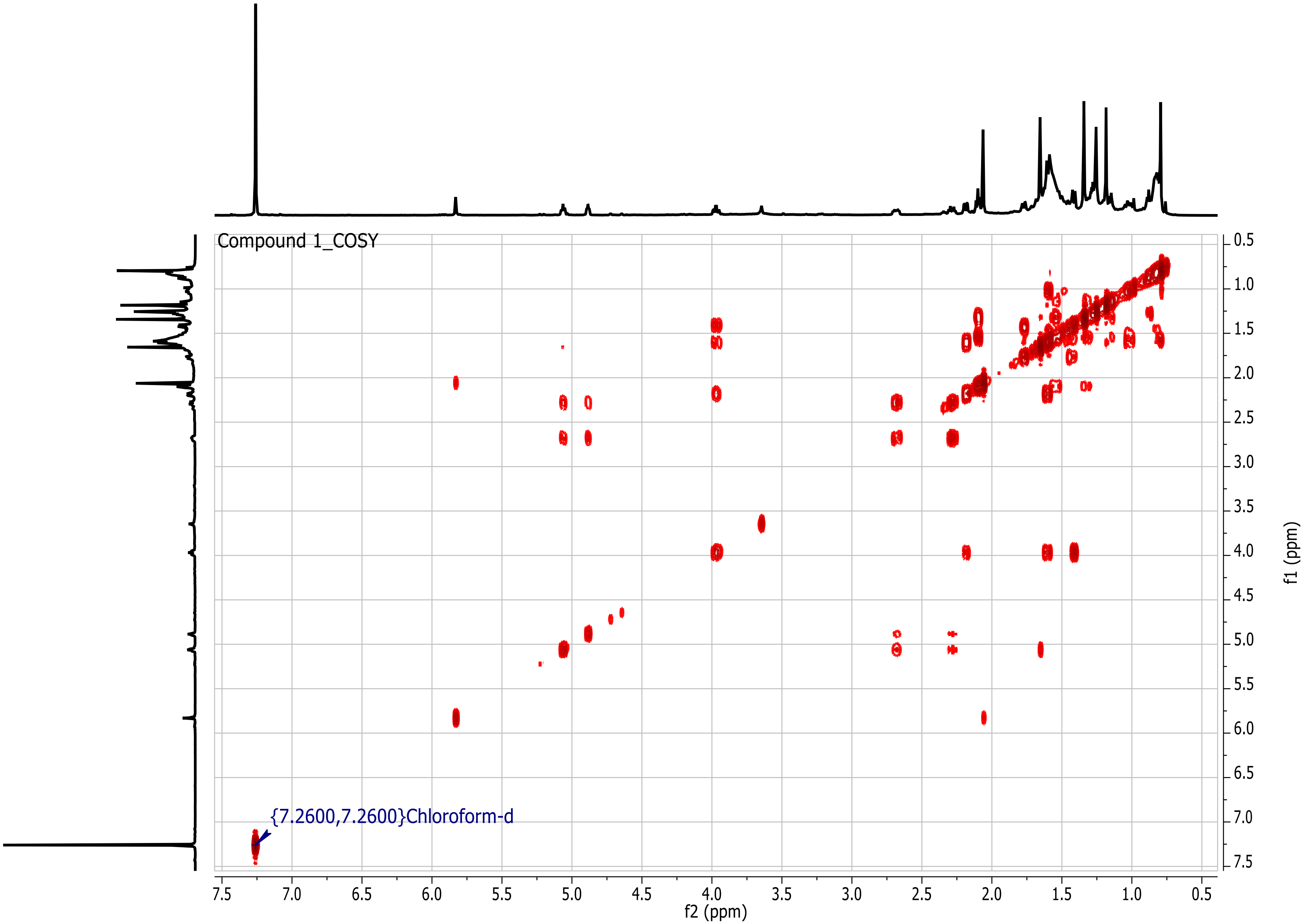

Figure S4. COSY (600 MHz, $CDCl_3$) spectrum of compound **1**.

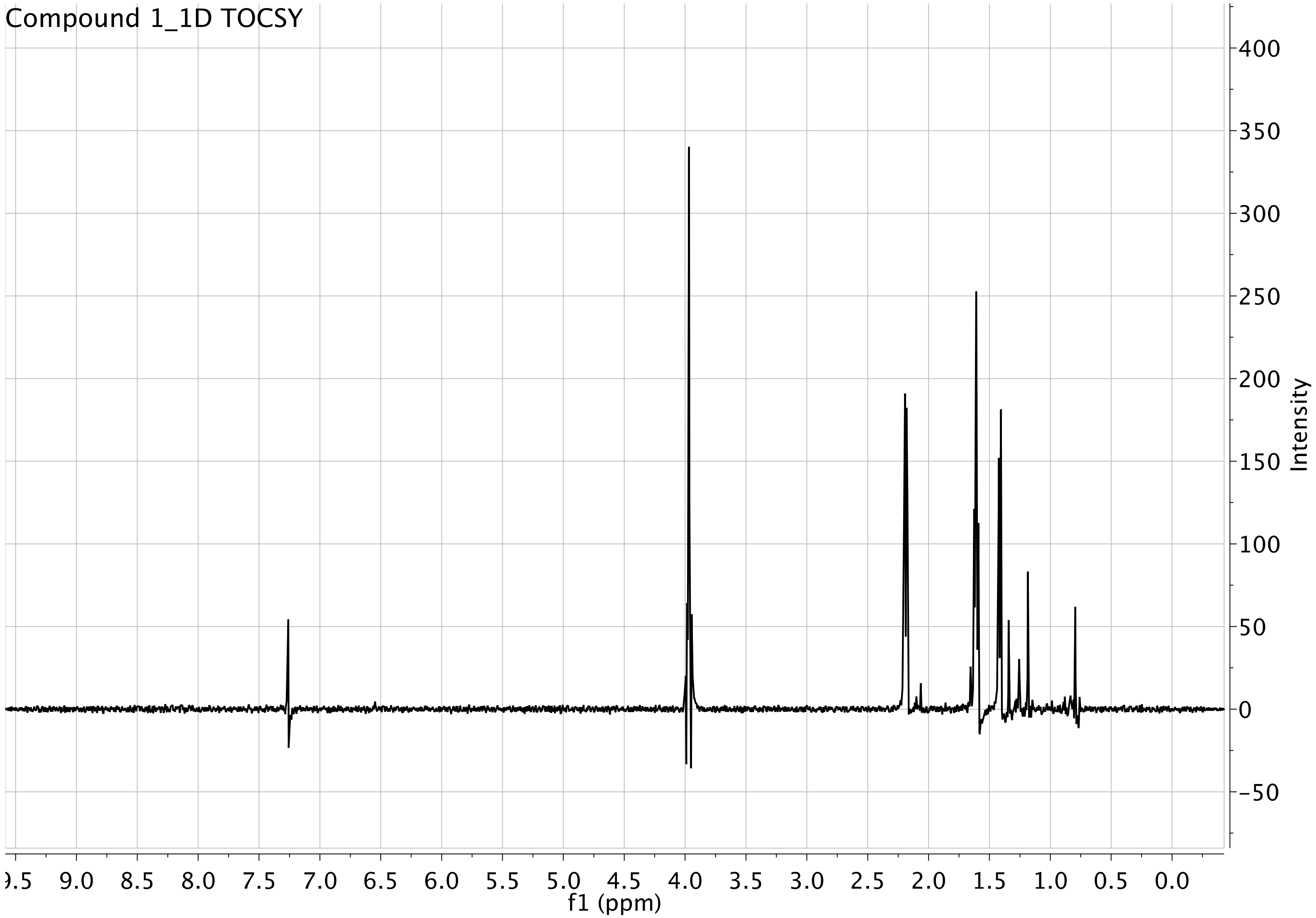

Figure S5. 1D TOCSY (600 MHz, $CDCl_3$) spectrum of compound **1**.

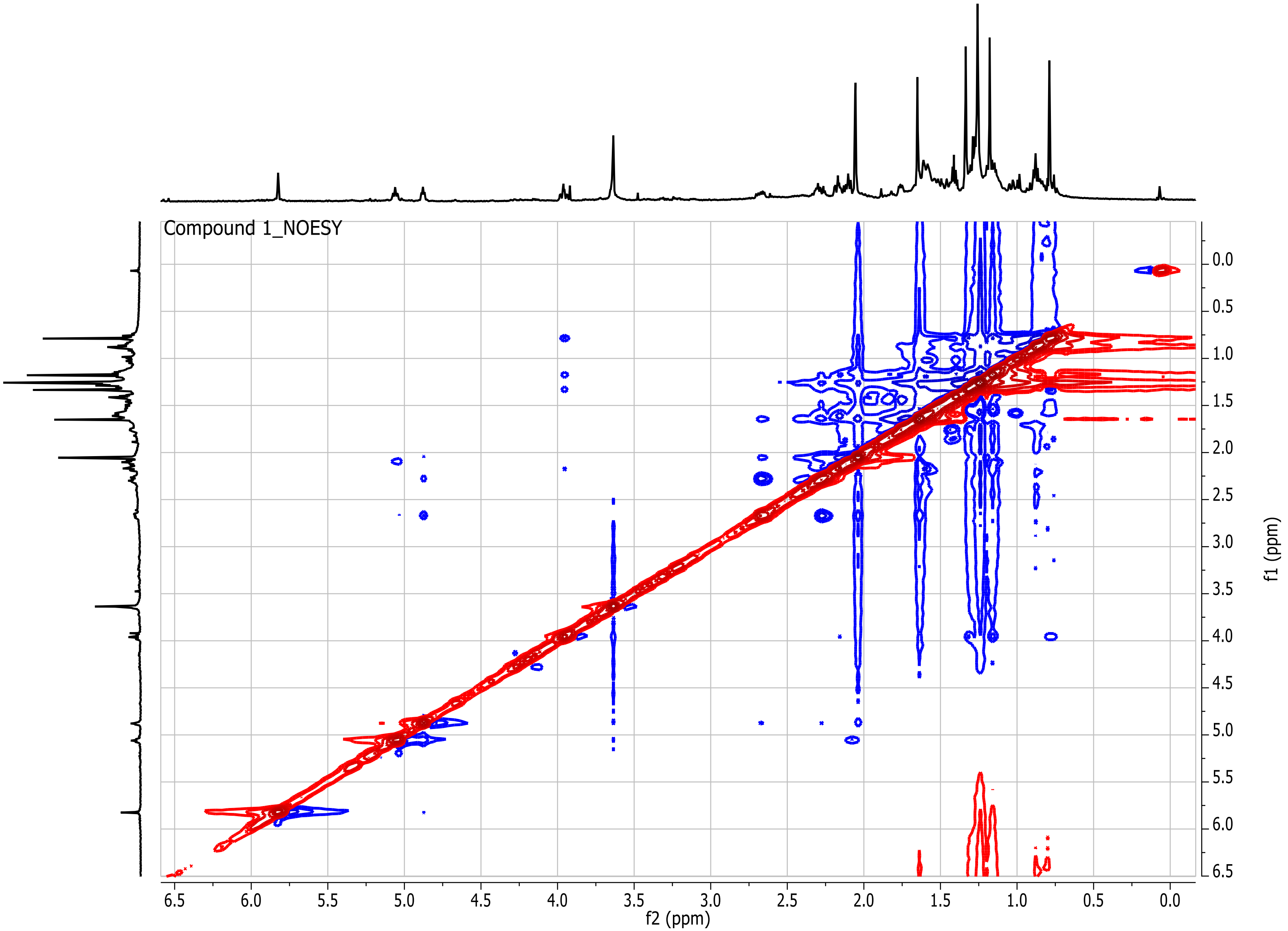

Figure S6. NOESY (600 MHz, $CDCl_3$) spectrum of compound **1**.

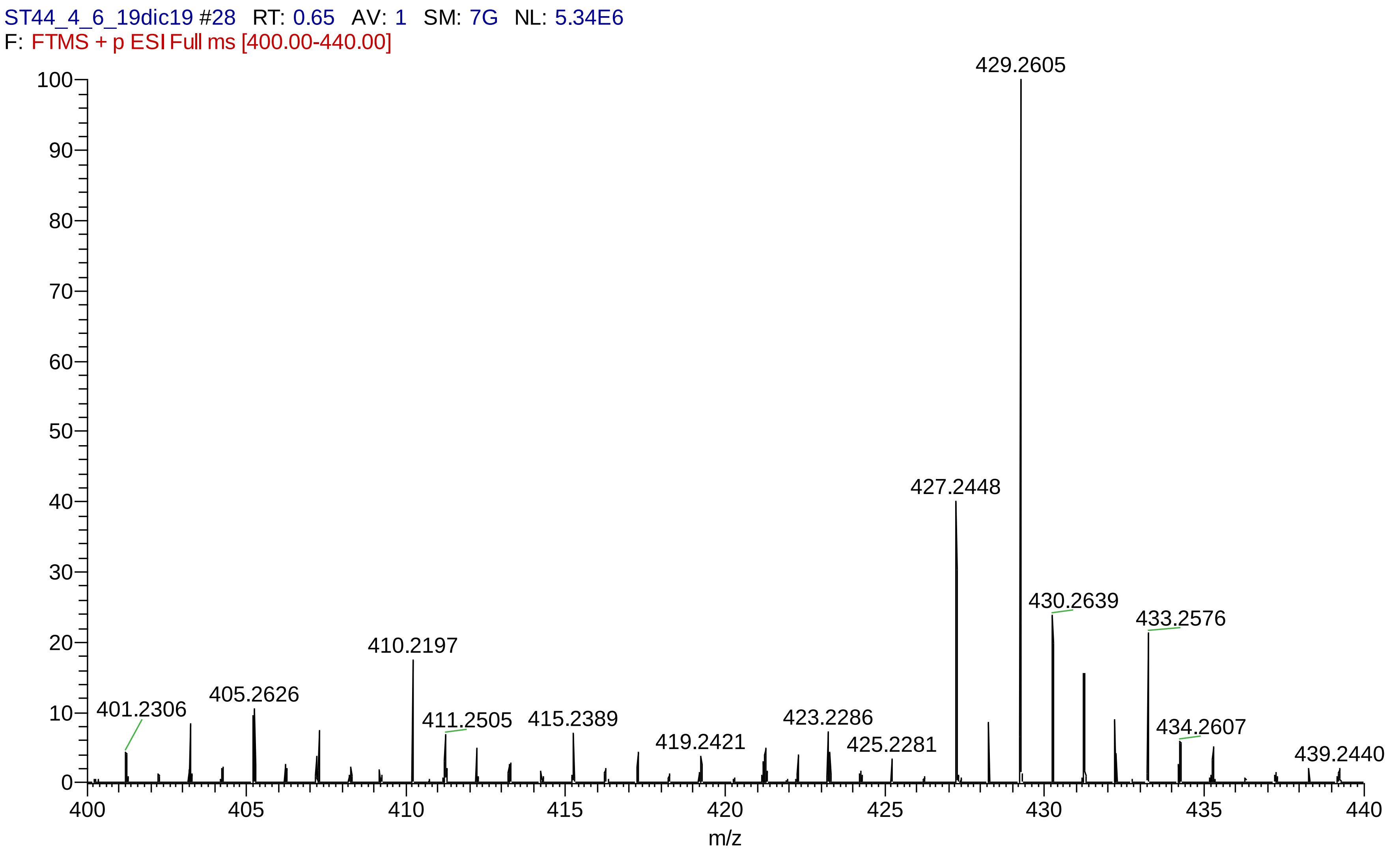

Figure S7. HRESIMS spectrum of compound **1**.

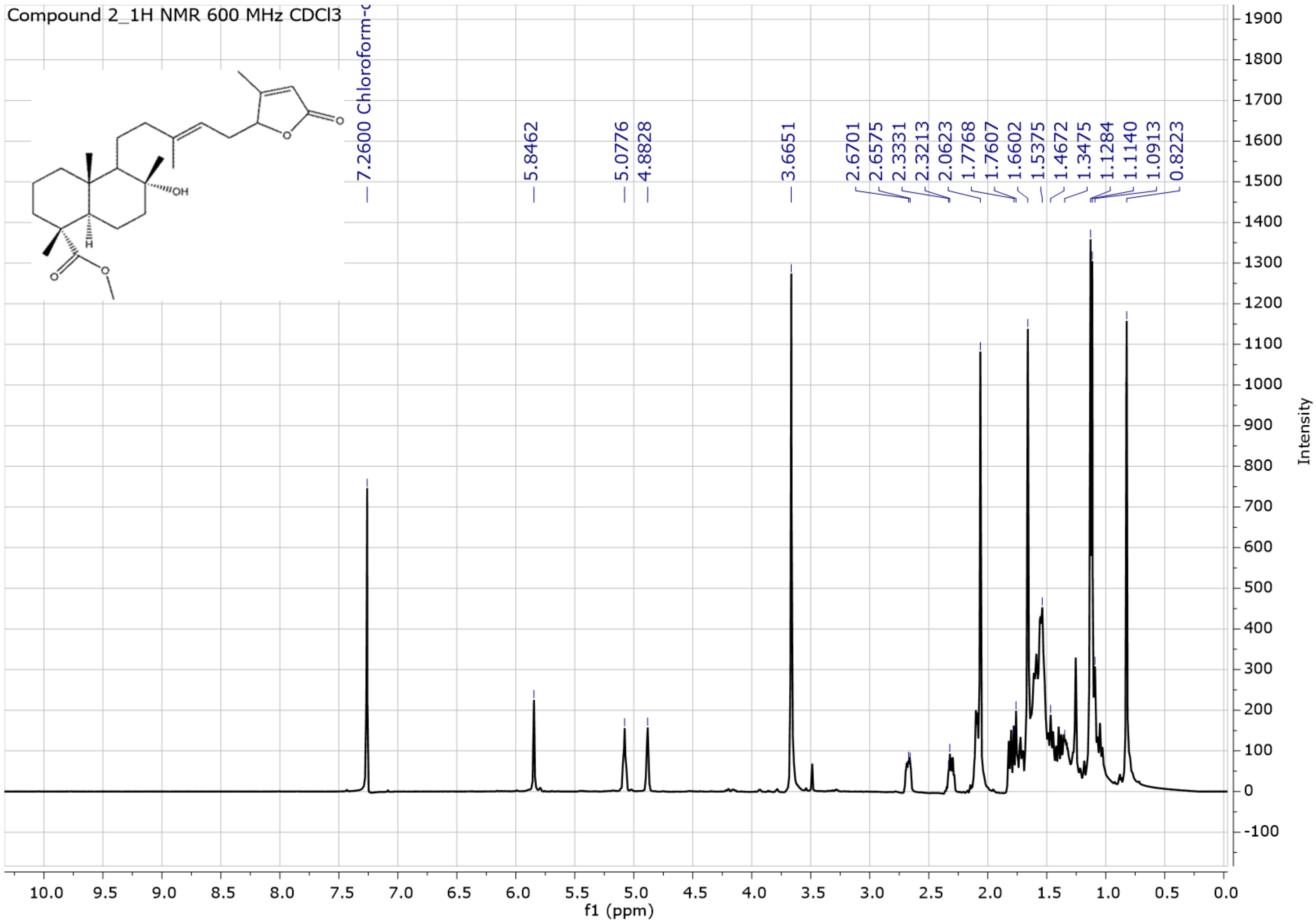

Figure S8. ^{1}H NMR (600 MHz, $CDCl_3$) spectrum of compound **2**.

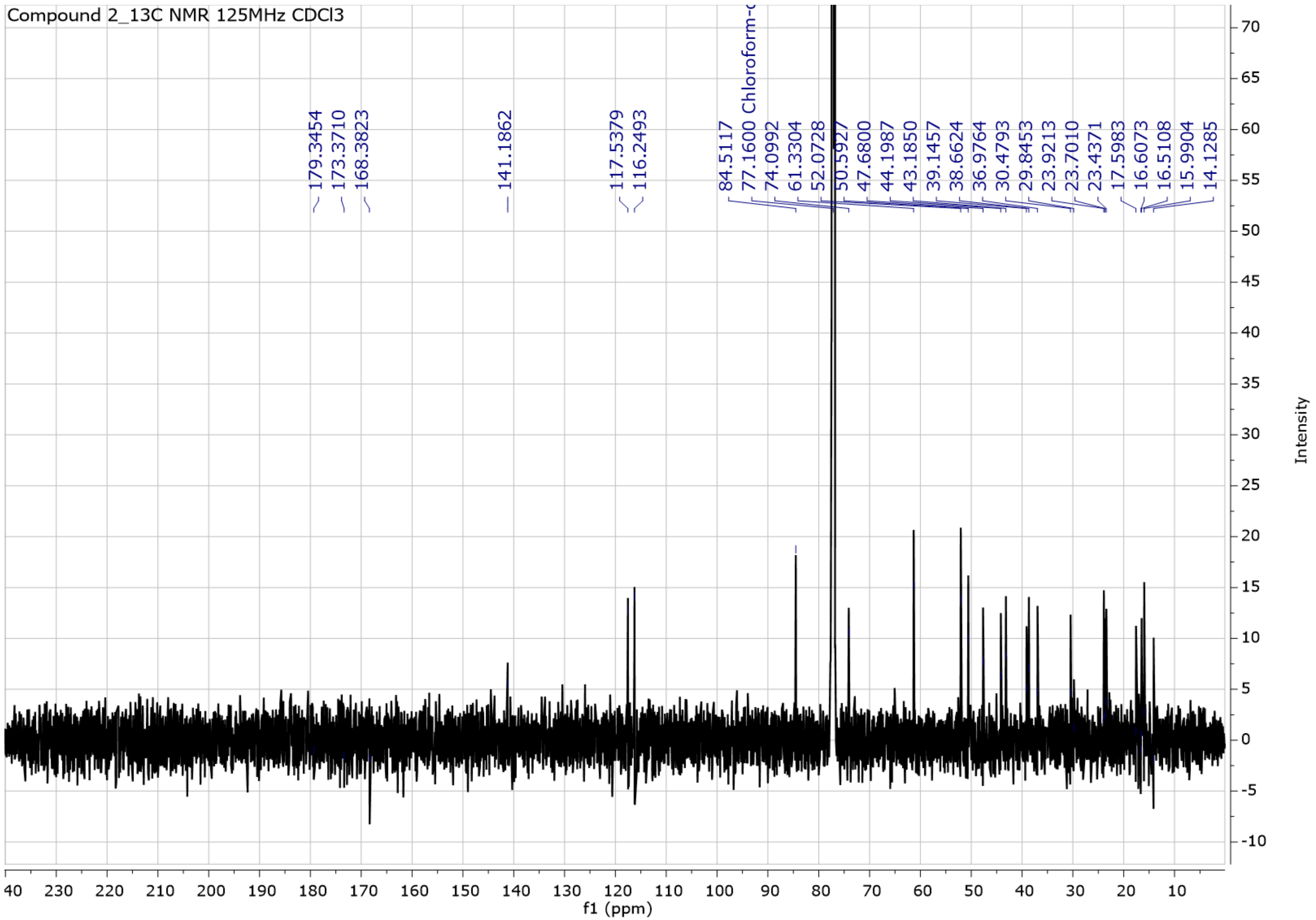

Figure S9. ^{13}C NMR (125 MHz, $CDCl_3$) spectrum of compound **2**.

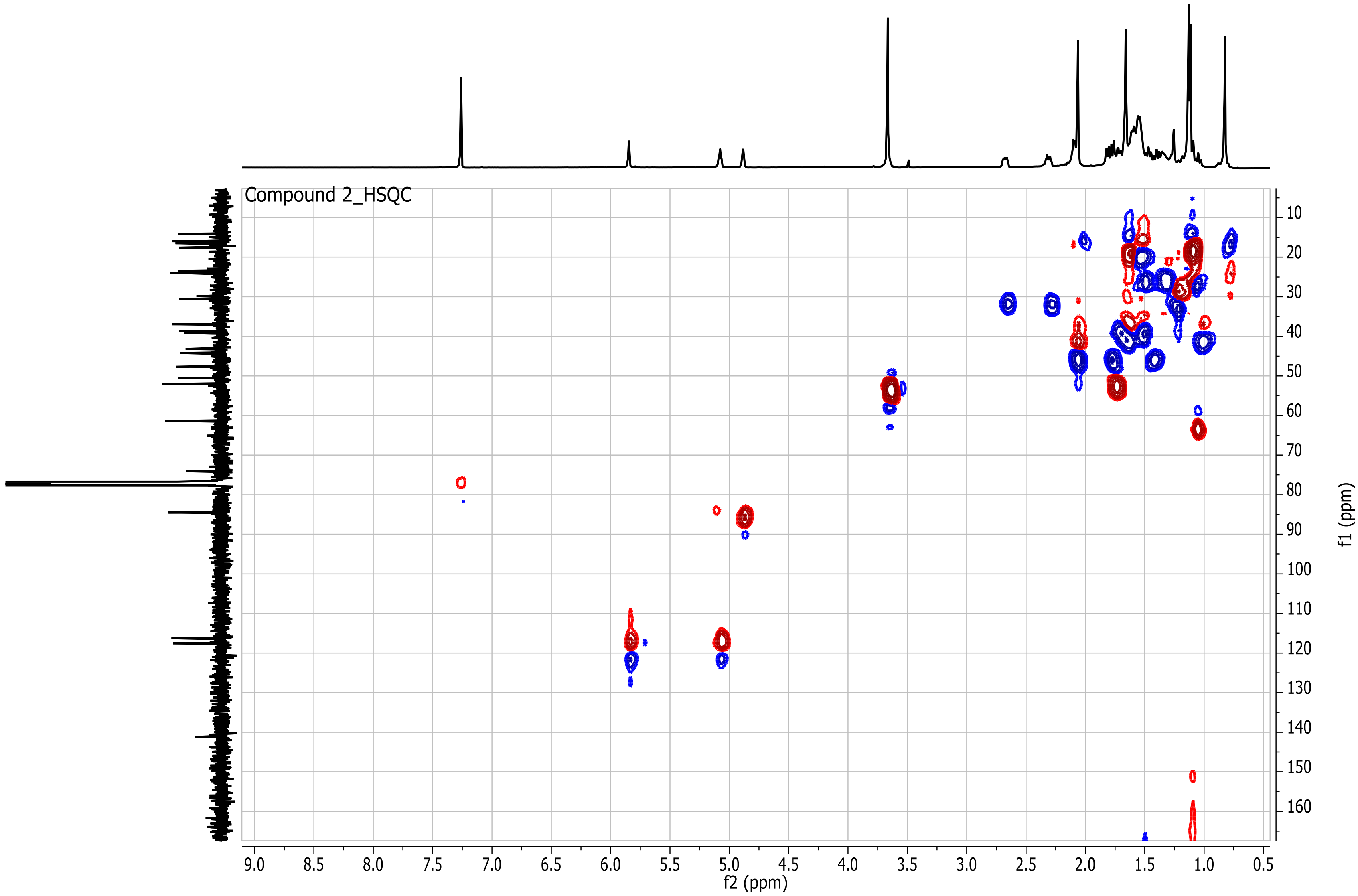

Figure S10. HSQC (600 MHz, $CDCl_3$) spectrum of compound **2**.

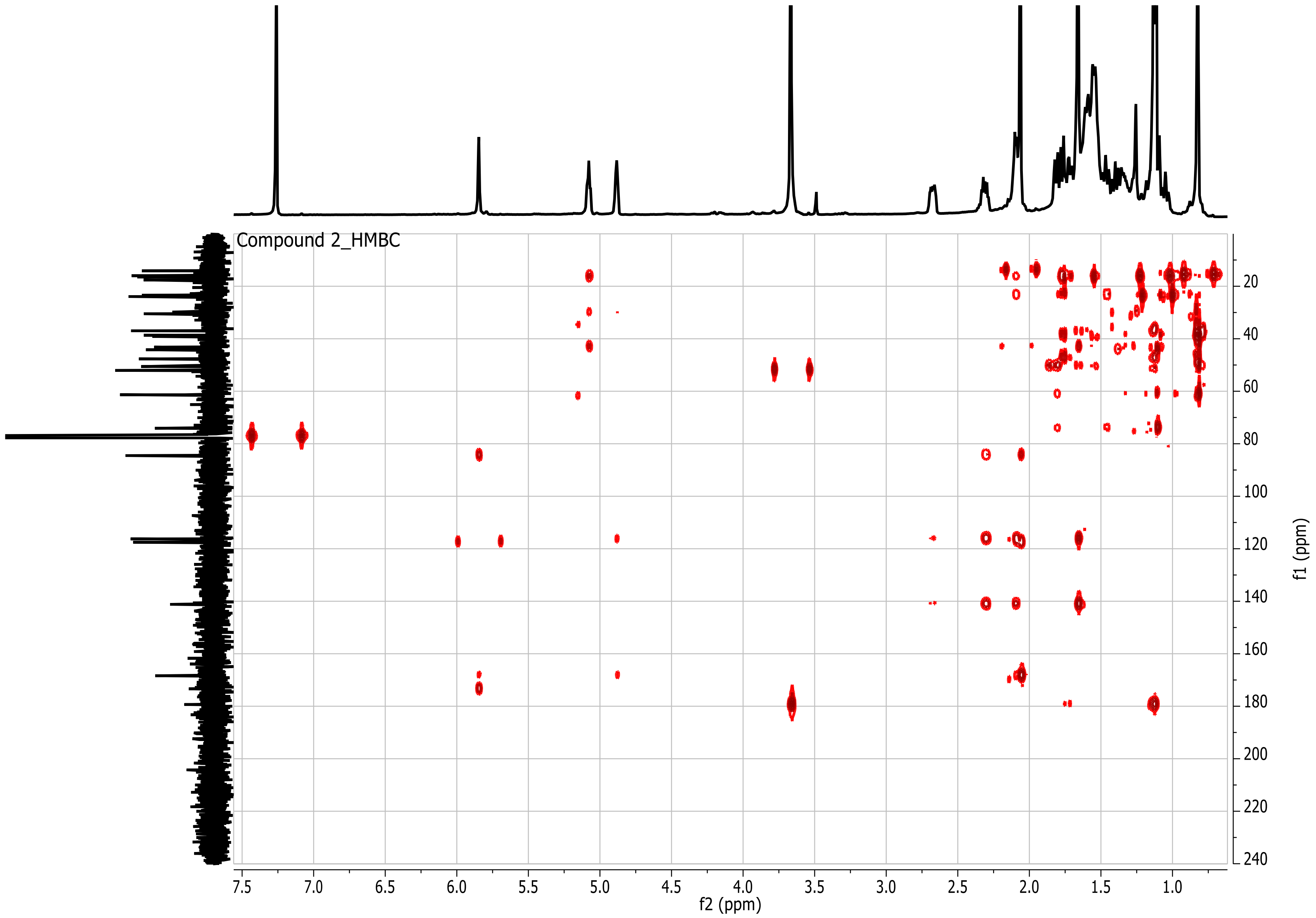

Figure S11. HMBC (600 MHz, $CDCl_3$) spectrum of compound **2**.

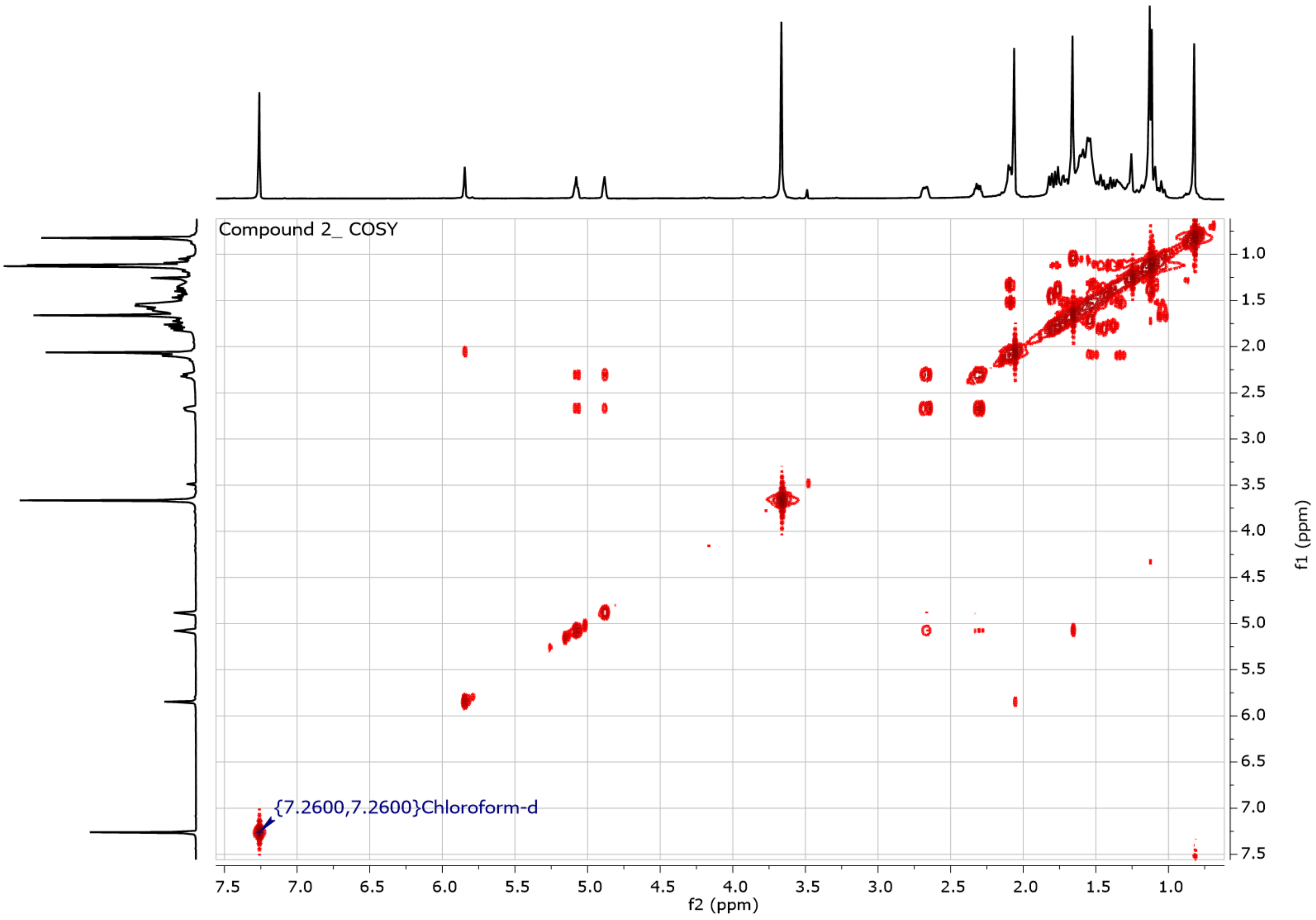

Figure S12. COSY (600 MHz, $CDCl_3$) spectrum of compound **2**.

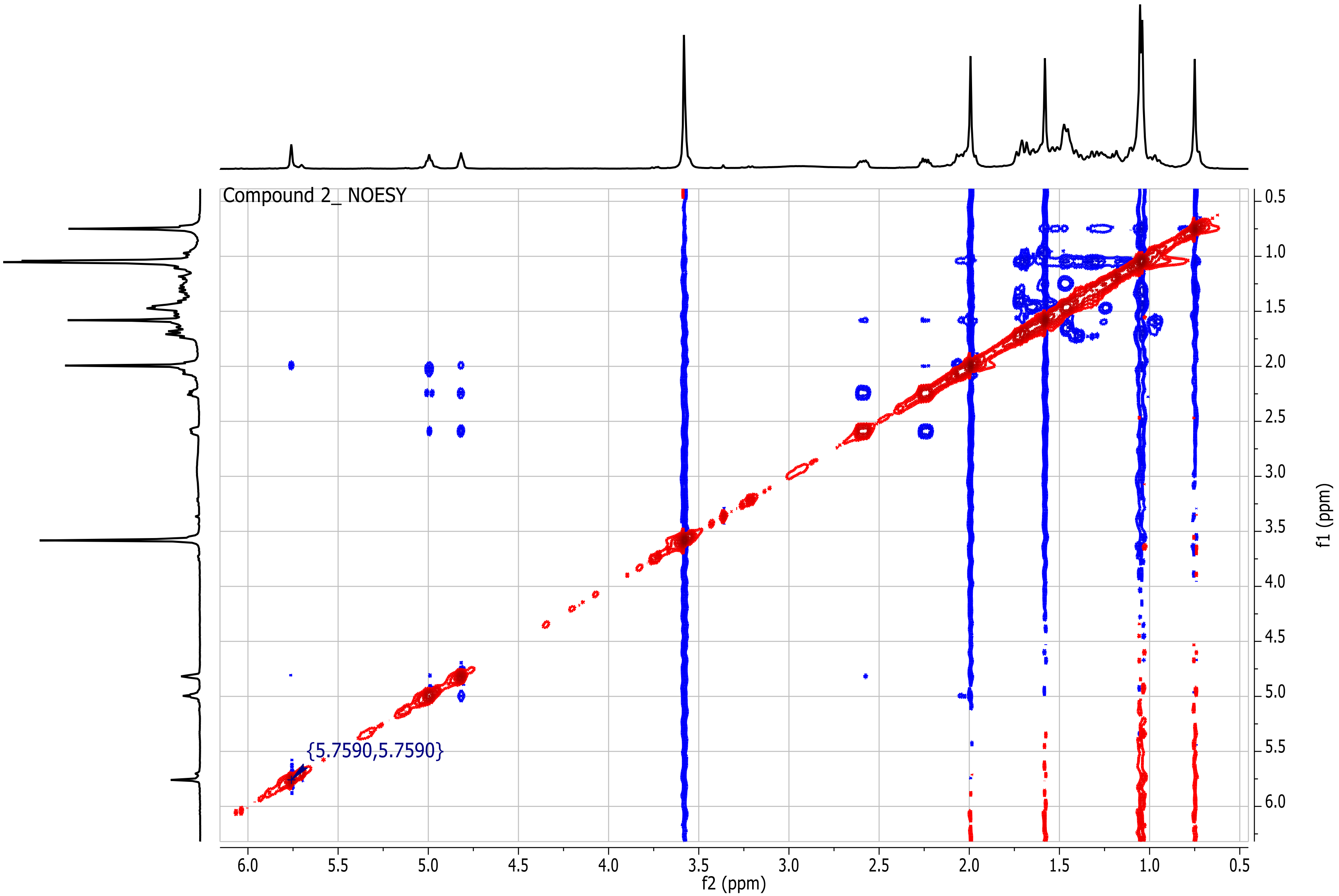

Figure S13. NOESY (600 MHz, $CDCl_3$) spectrum of compound **2**.

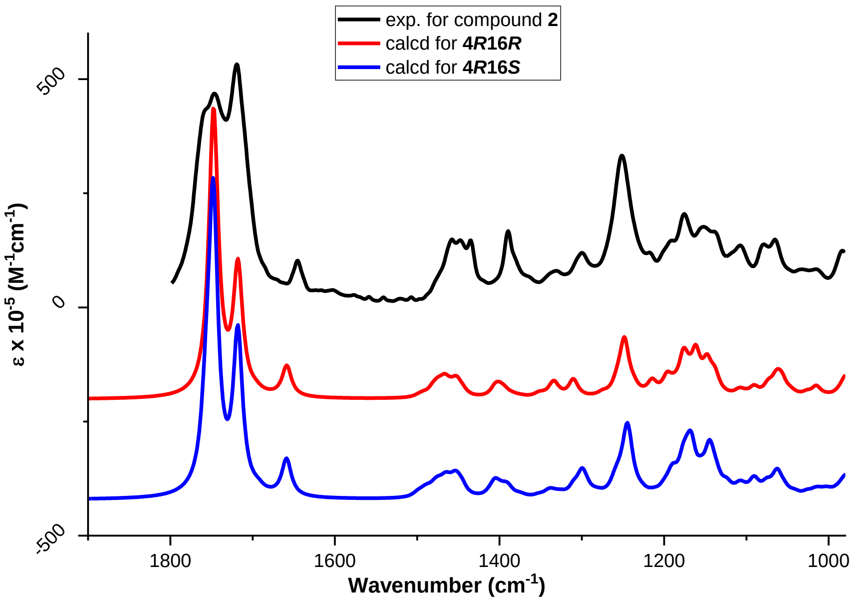

Figure S14. Comparison of experimental and computed IR spectra ($CDCl_3$) for compound **2**.
4*R* stands for 4*R*,5*R*,8*R*,9*R*,10*S*. The wavenumber scale factor 0.9835 was used to scale the computed spectra.

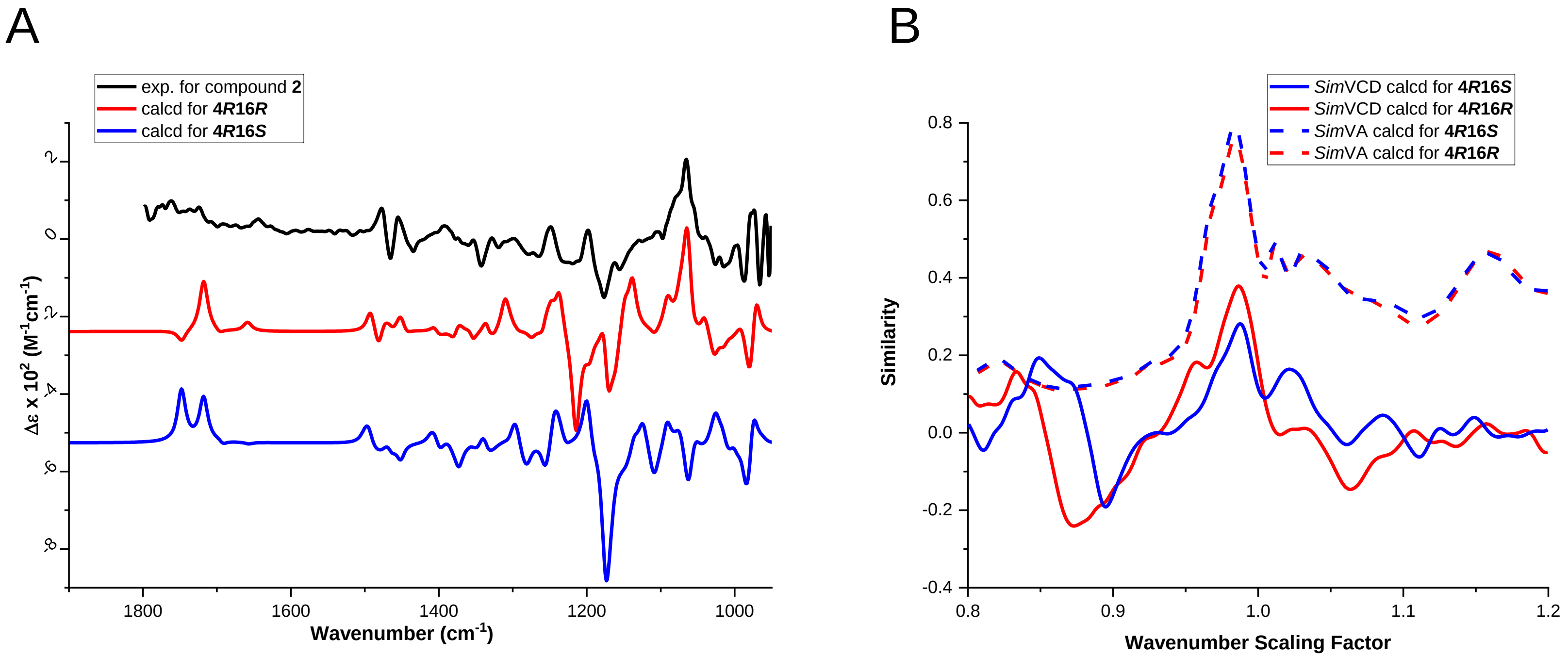

Figure S15. Comparison of experimental and computed VCD spectra in $CDCl_3$ for compound **2**.
(A). Similarities (*Sim*VA and *Sim*VCD) between experimental and computed VA and VCD spectra of **2** were plotted as functions of wavenumber scale factor (B). 4*R* stands for 4*R*,5*R*,8*R*,9*R*,10*S*. The wavenumber scale factor corresponding to the maximal *Sim*VA value in B (0.9835) was used to scale the computed spectra in A.

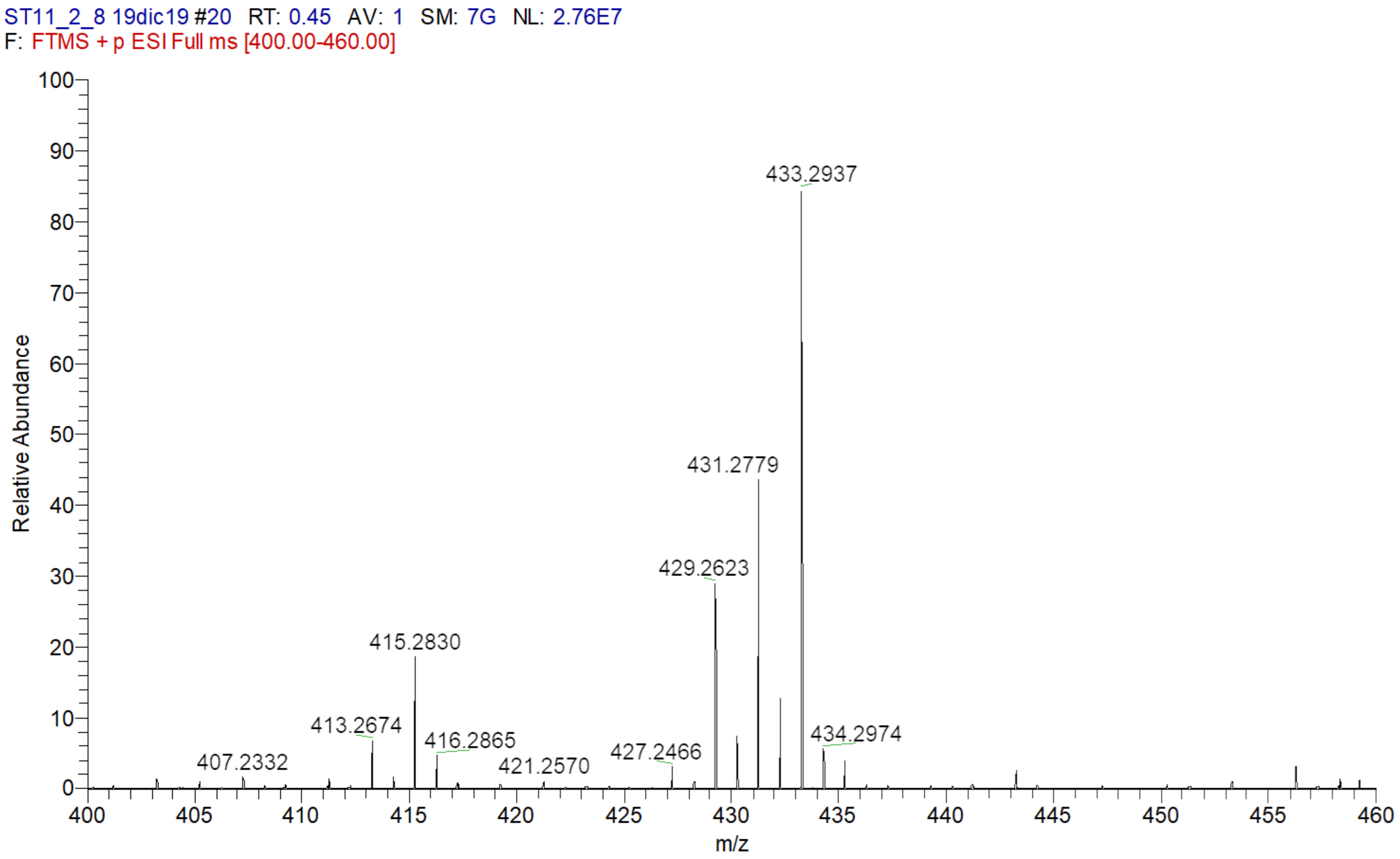

Figure S16. HRESIMS spectrum of compound **2**.

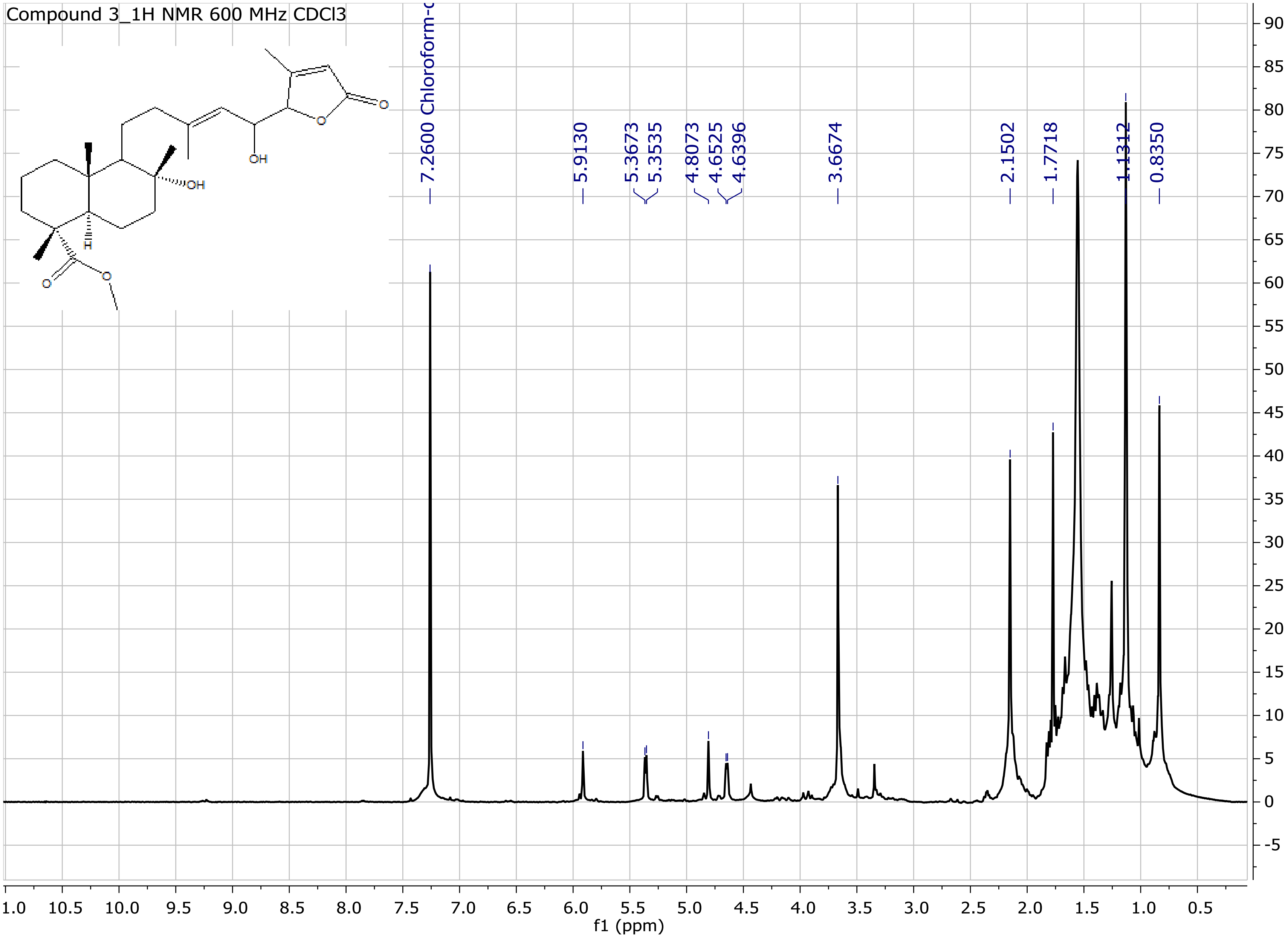

Figure S17. ^{1}H NMR (600 MHz, $CDCl_3$) spectrum of compound **3**.

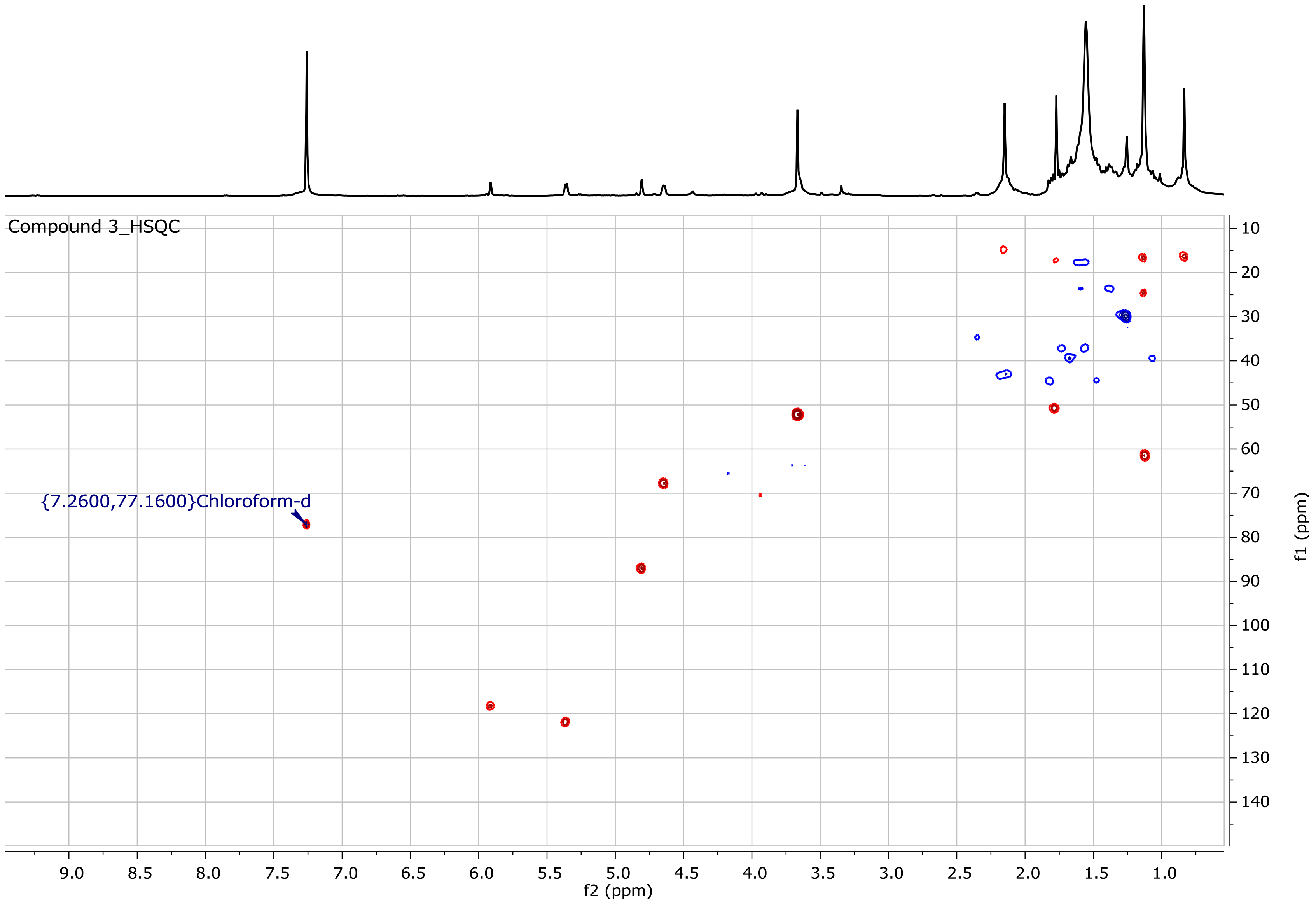

Figure S18. HSQC (600 MHz, $CDCl_3$) spectrum of compound **3**.

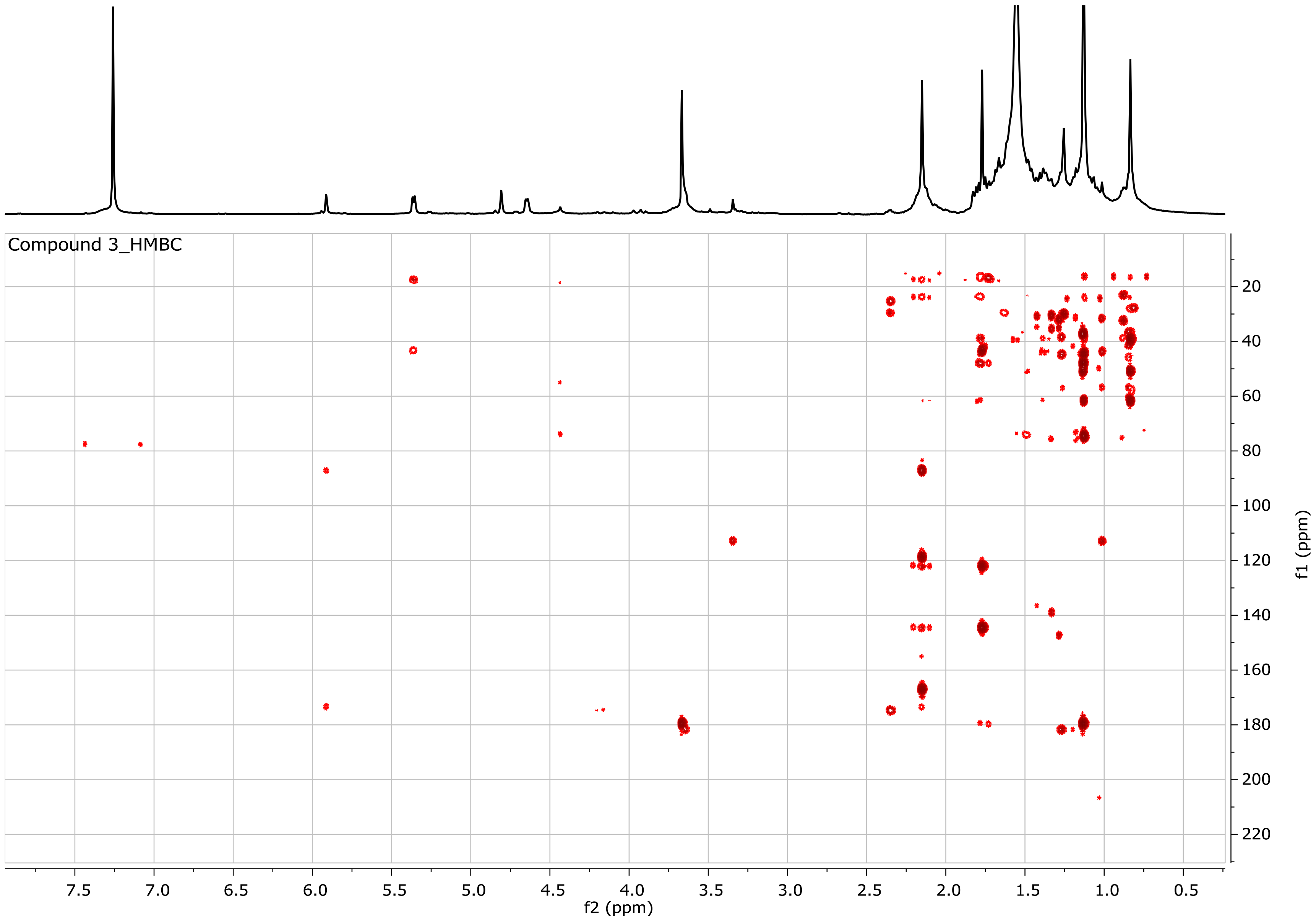

Figure S19. HMBC (600 MHz, $CDCl_3$) spectrum of compound **3**.

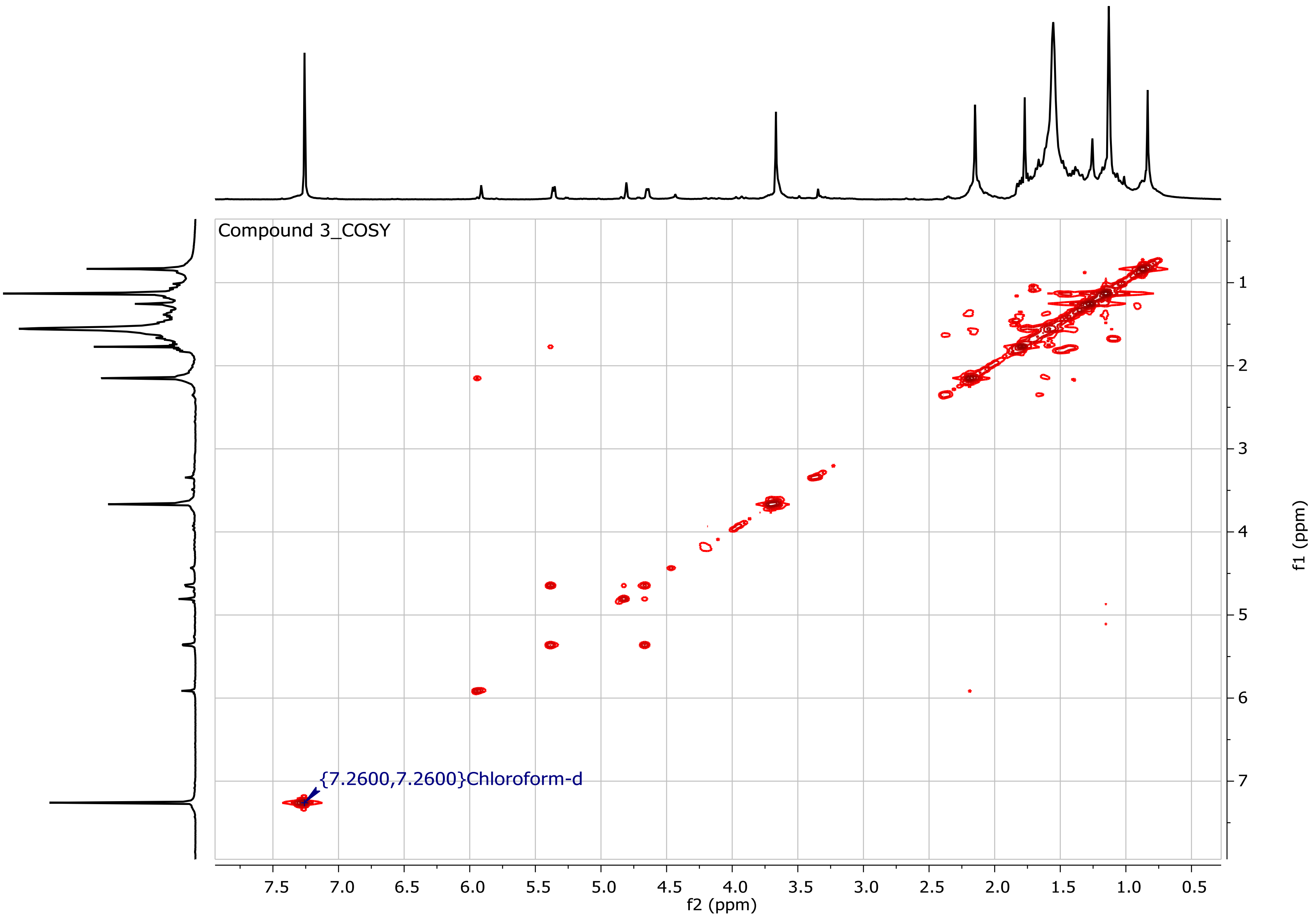

Figure S20. COSY (600 MHz, $CDCl_3$) spectrum of compound **3**.

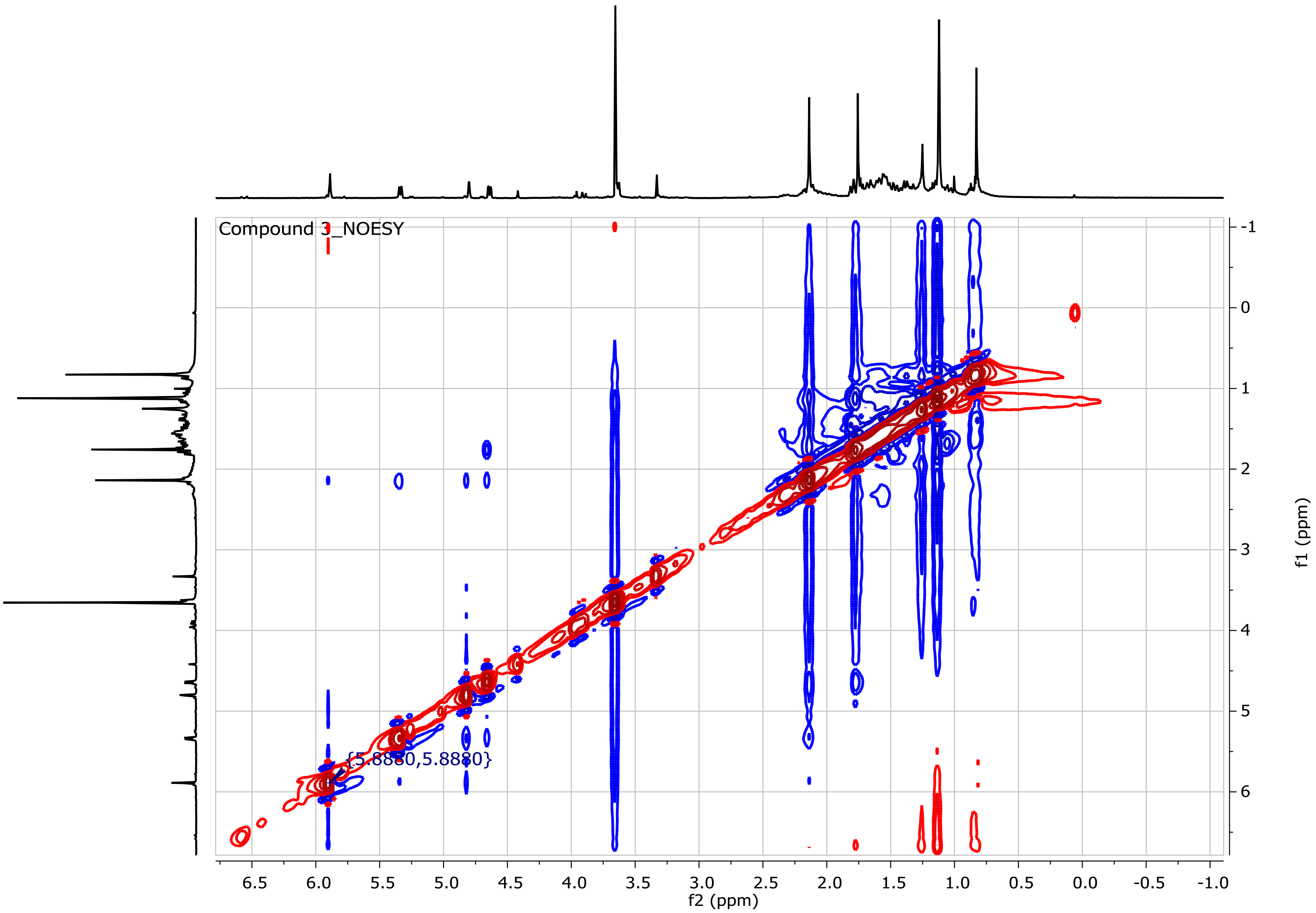

Figure S21. NOESY (500 MHz, $CDCl_3$) spectrum of compound **3**.

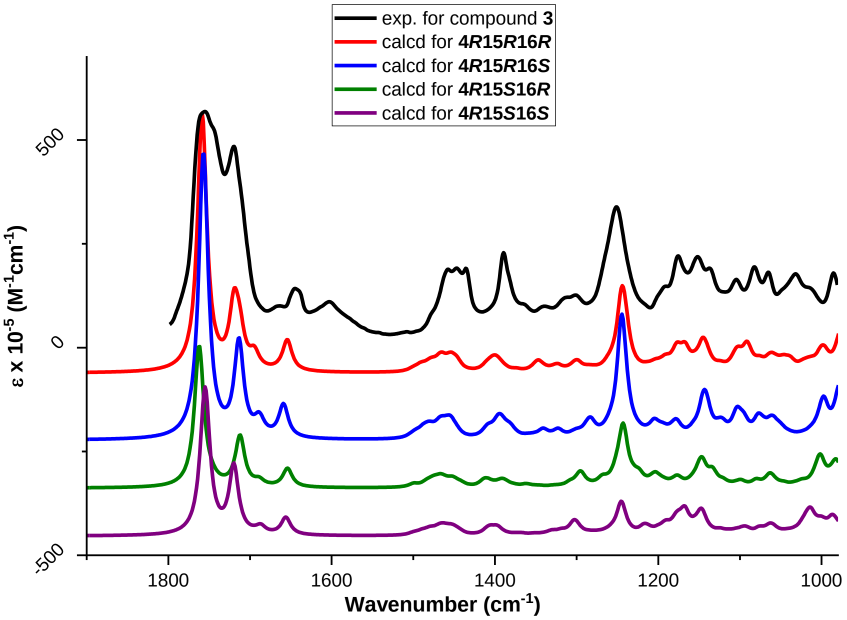

Figure S22. Comparison of experimental and computed IR spectra ($CDCl_3$) for compound **3**.
4*R* stands for 4*R*,5*R*,8*R*,9*R*,10*S*. The wavenumber scale factor 0.9845 was used to scale the computed spectra.

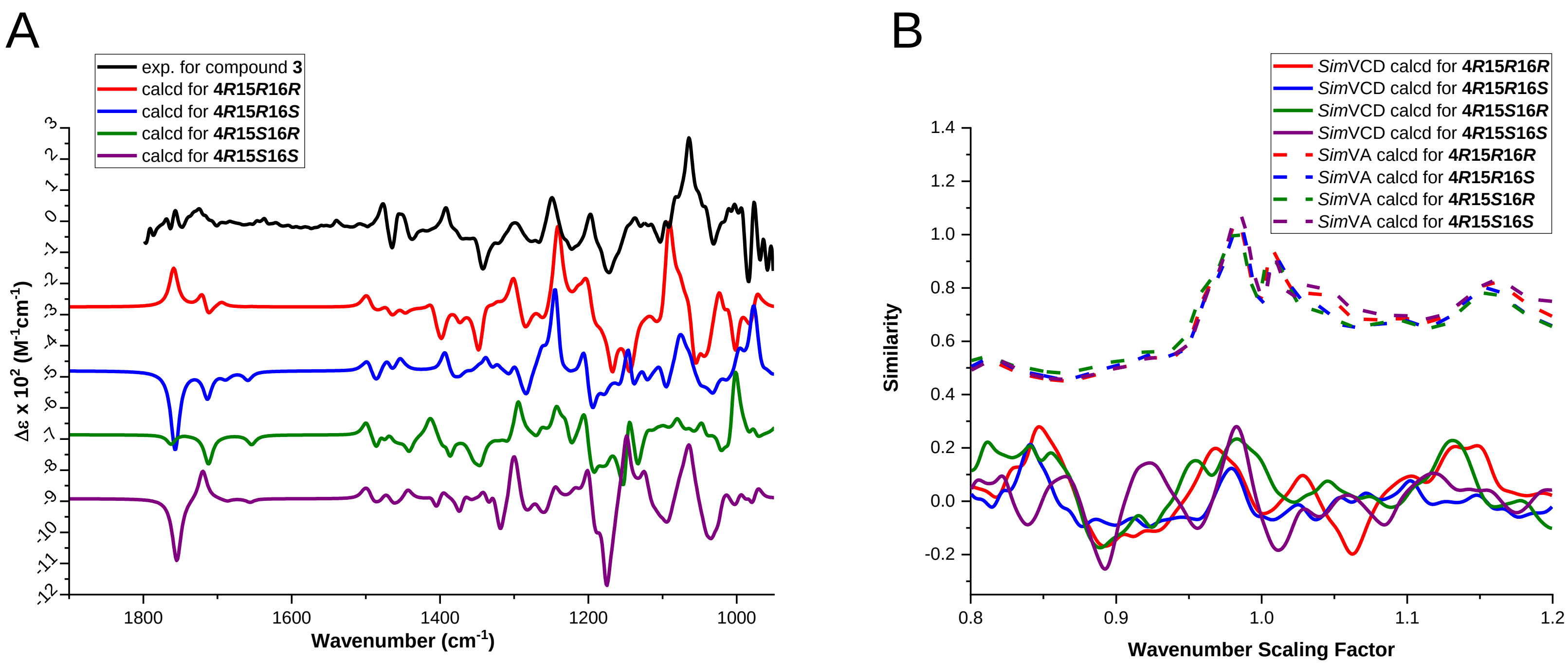

Figure S23. Comparison of experimental and computed VCD spectra in $CDCl_3$ for compound **3**.
(A). Similarities (*Sim*VA and *Sim*VCD) between experimental and computed VA and VCD spectra of **3** were plotted as functions of wavenumber scale factor (B). 4*R* stands for 4*R*,5*R*,8*R*,9*R*,10*S*. The wavenumber scale factor corresponding to the maximal *Sim*VA value in B (0.9845) was used to scale the computed spectra in A.

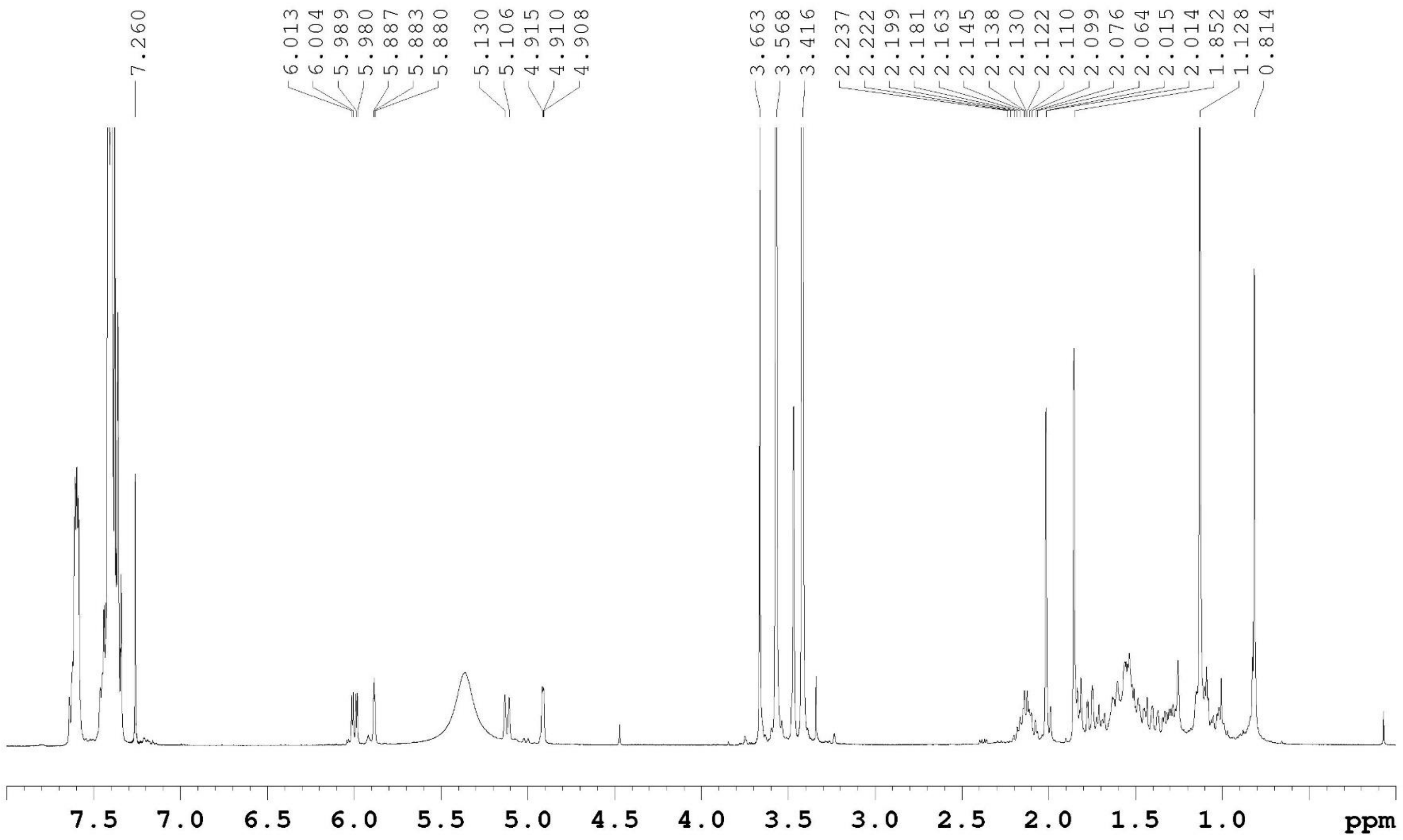

Figure S24. ^{1}H NMR (400 MHz, $CDCl_3$) spectrum of S-MTPA ester of compound **3** (by esterification of **3** with (R)-(-)-MTPA-Cl).

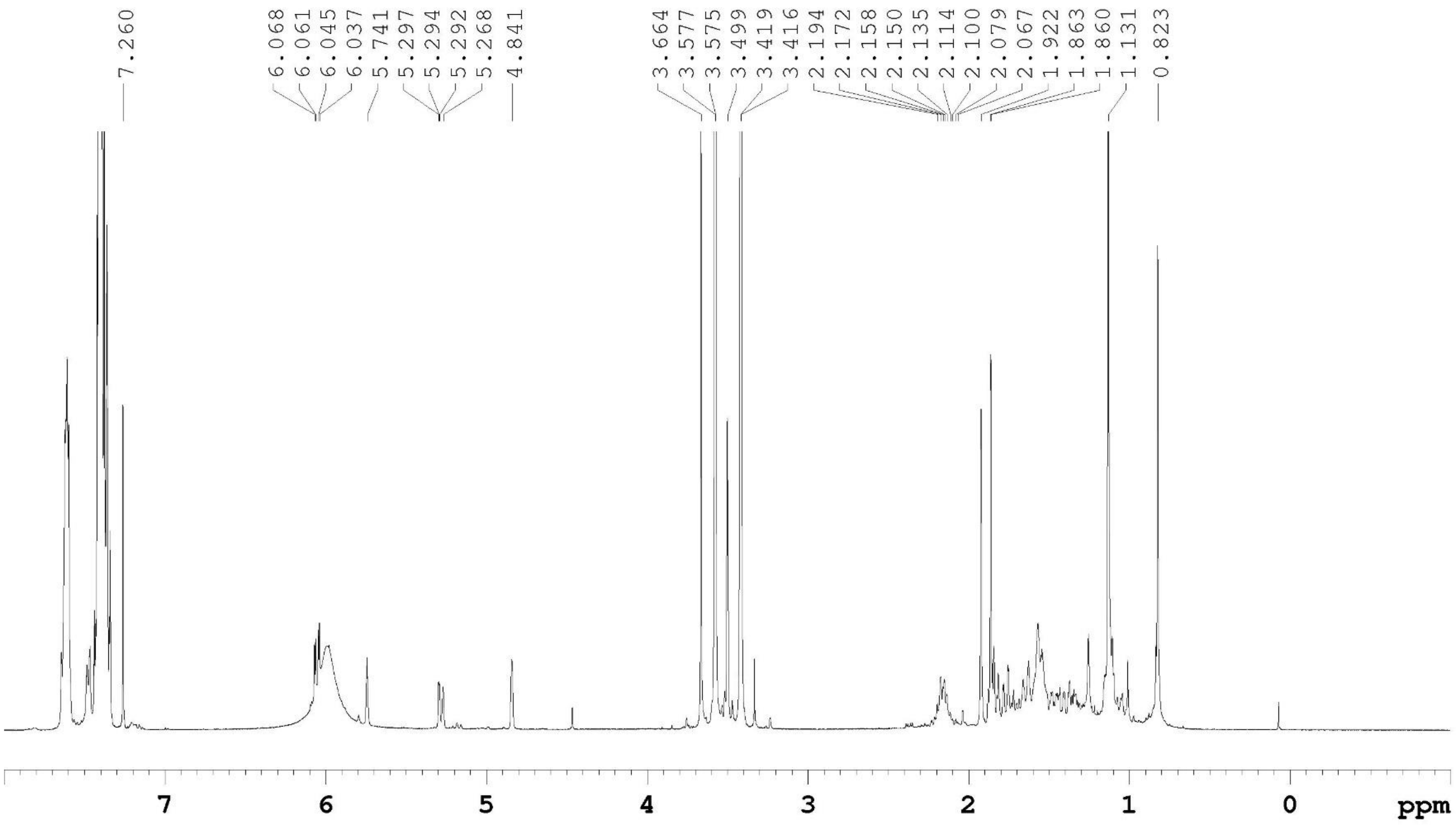

Figure S25. ^{1}H NMR (400 MHz, $CDCl_3$) spectrum of R-MTPA ester of compound **3** (by esterification of **3** with (S)-(+)-MTPA-Cl).

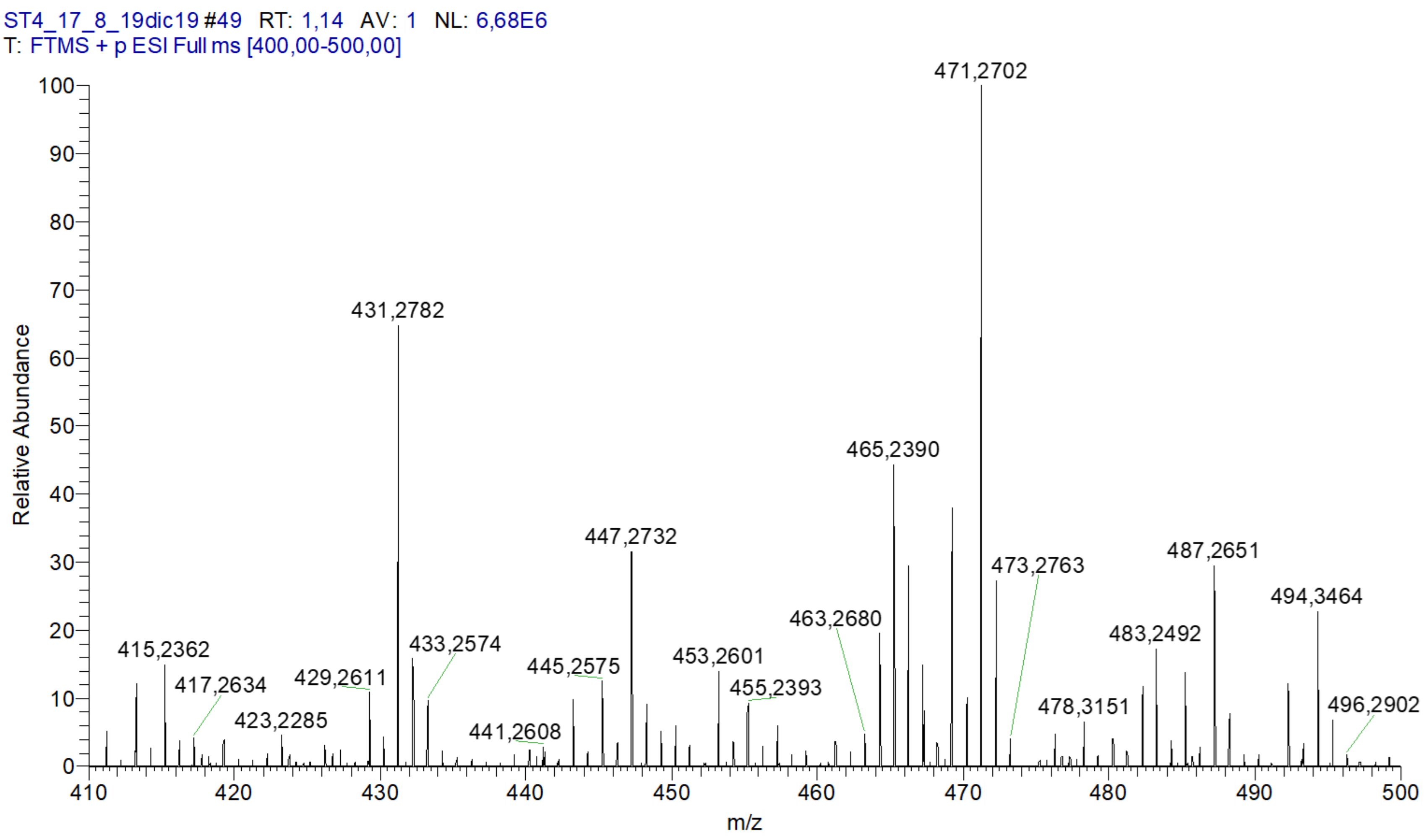

Figure S26. HRESIMS spectrum of compound **3**.

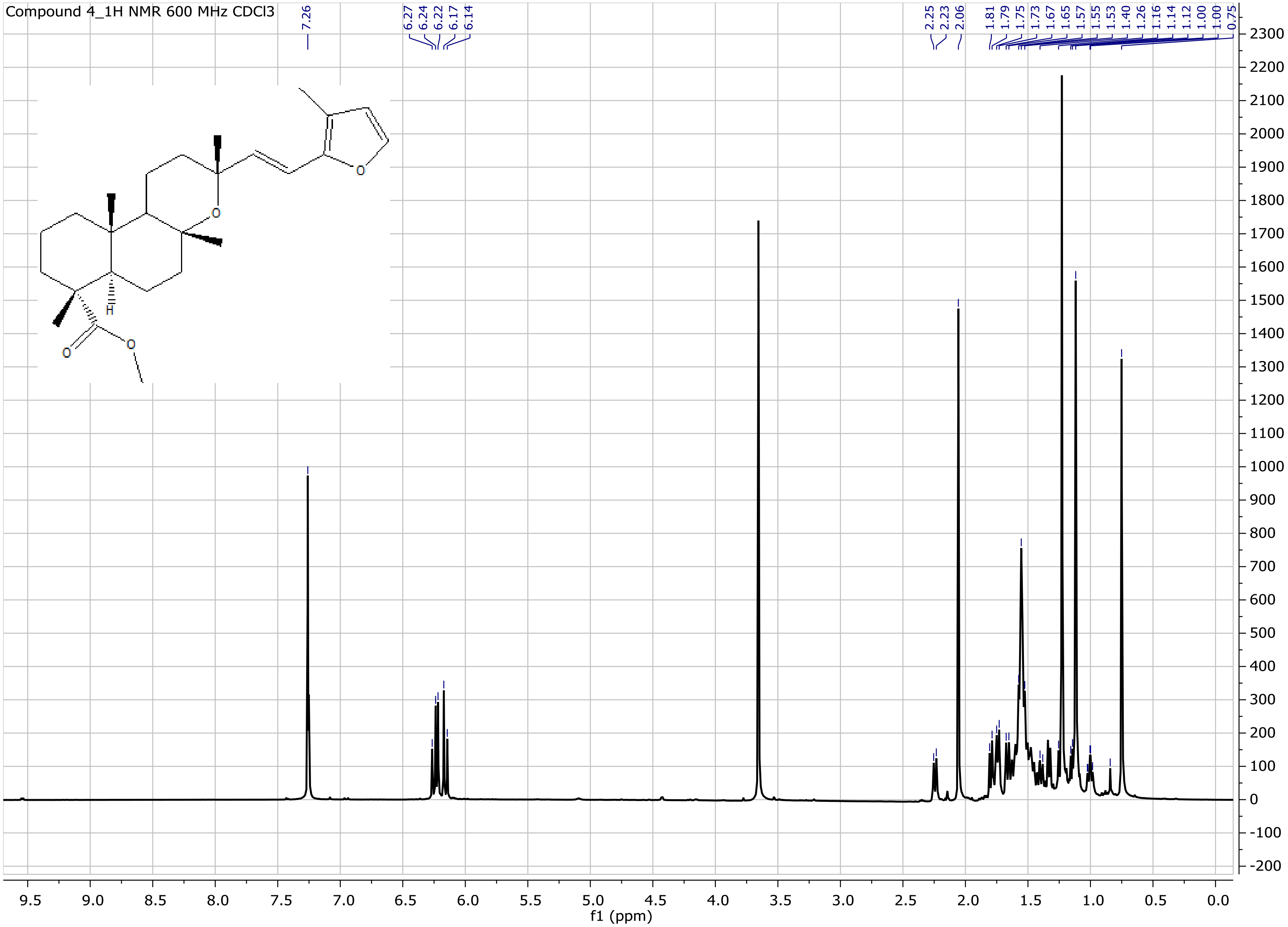

Figure S27. ^{1}H NMR (600 MHz, $CDCl_3$) spectrum of compound **4**.

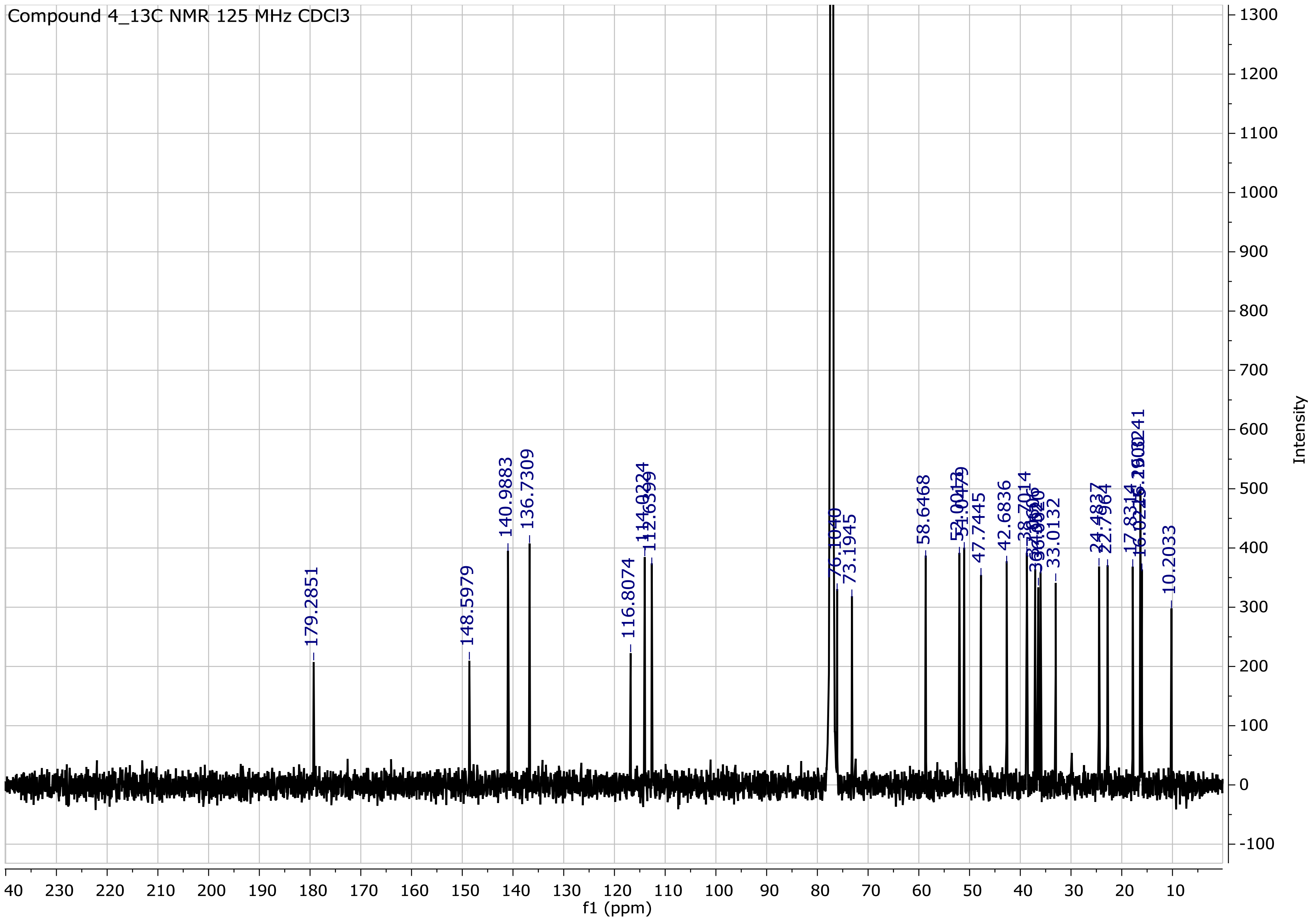

Figure S28. ^{13}C NMR (125 MHz, $CDCl_3$) spectrum of compound **4**.

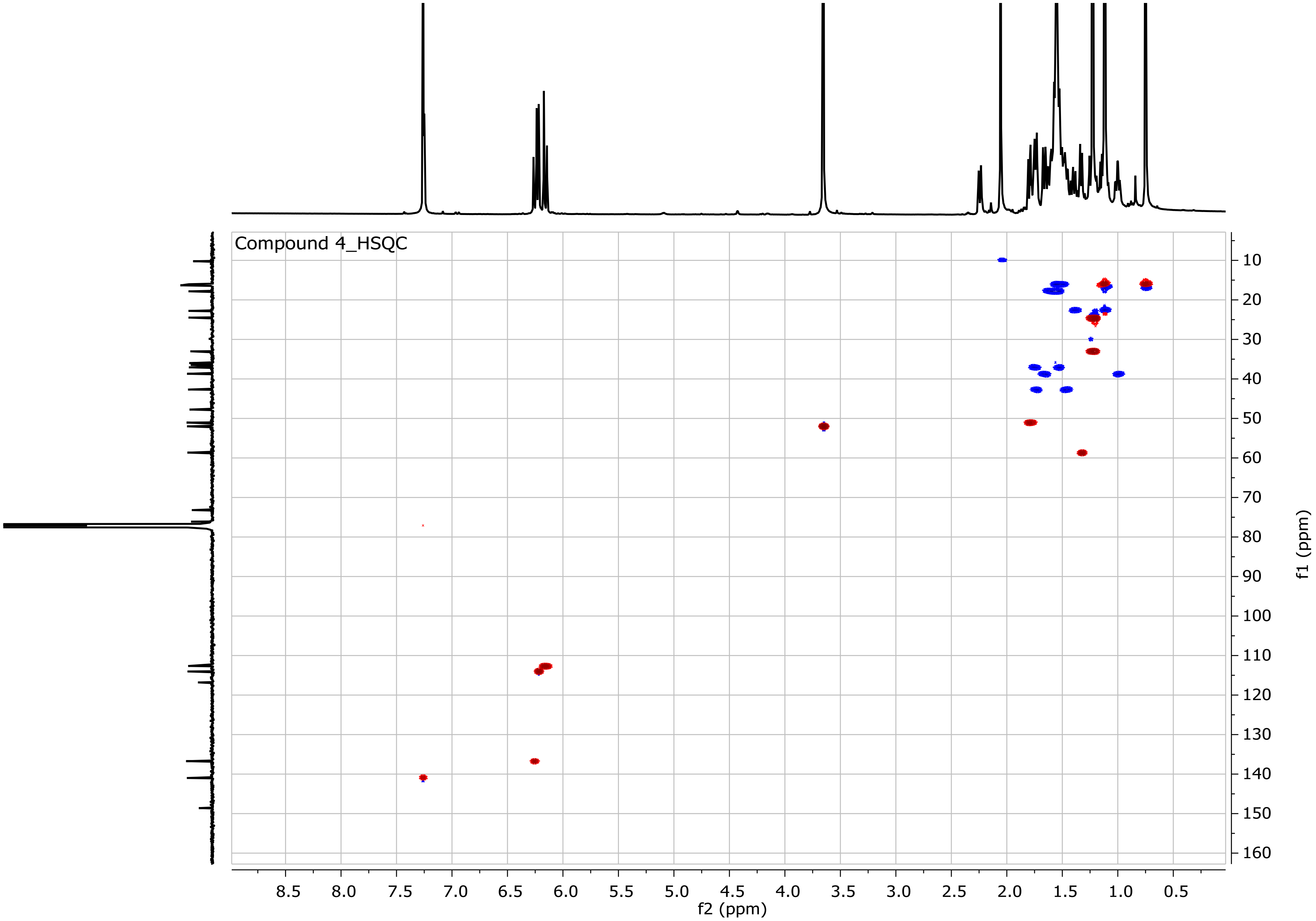

Figure S29. HSQC (600 MHz, $CDCl_3$) spectrum of compound **4**.

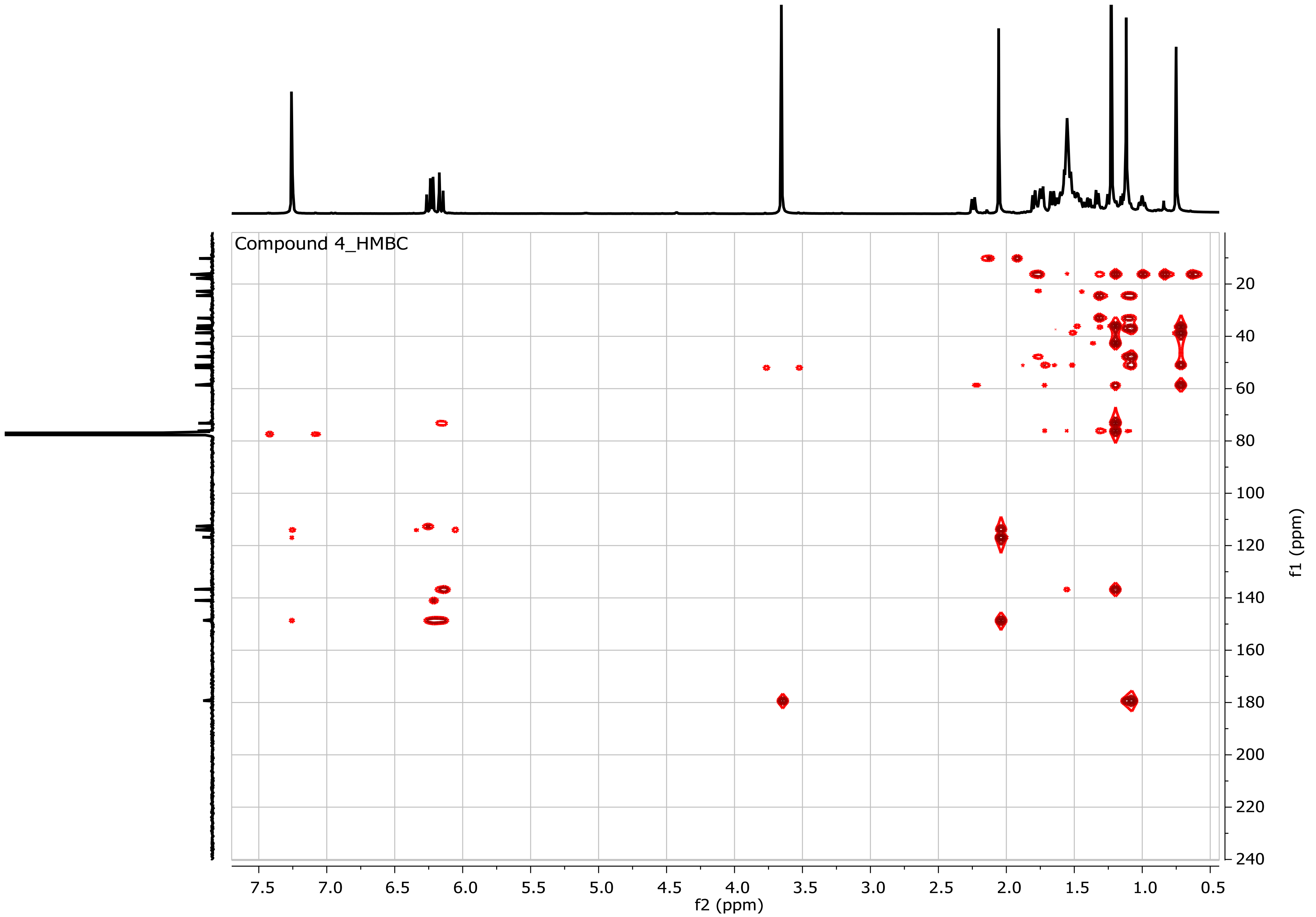

Figure S30. HMBC (600 MHz, $CDCl_3$) spectrum of compound **4**.

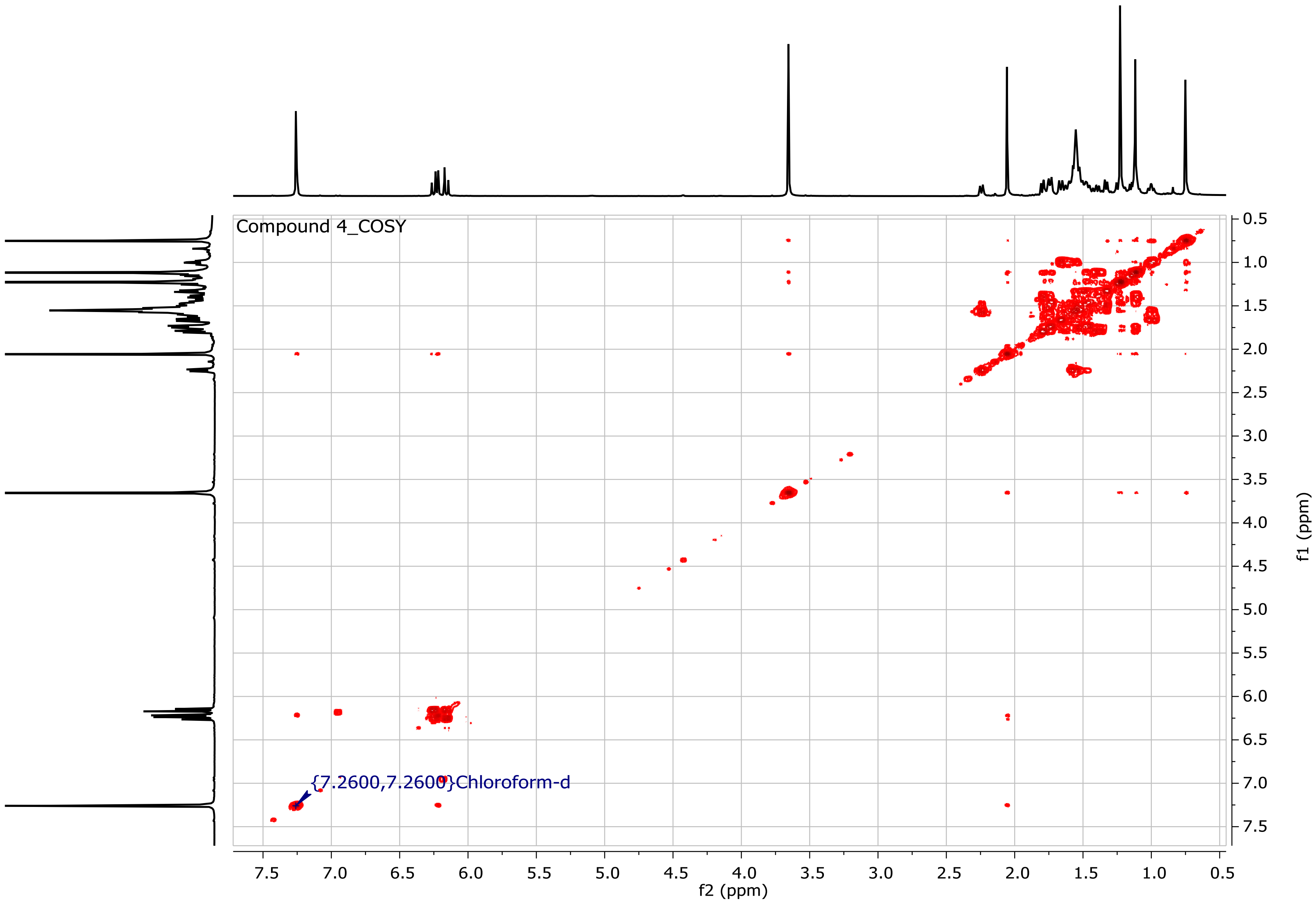

Figure S31. COSY (600 MHz, $CDCl_3$) spectrum of compound **4**.

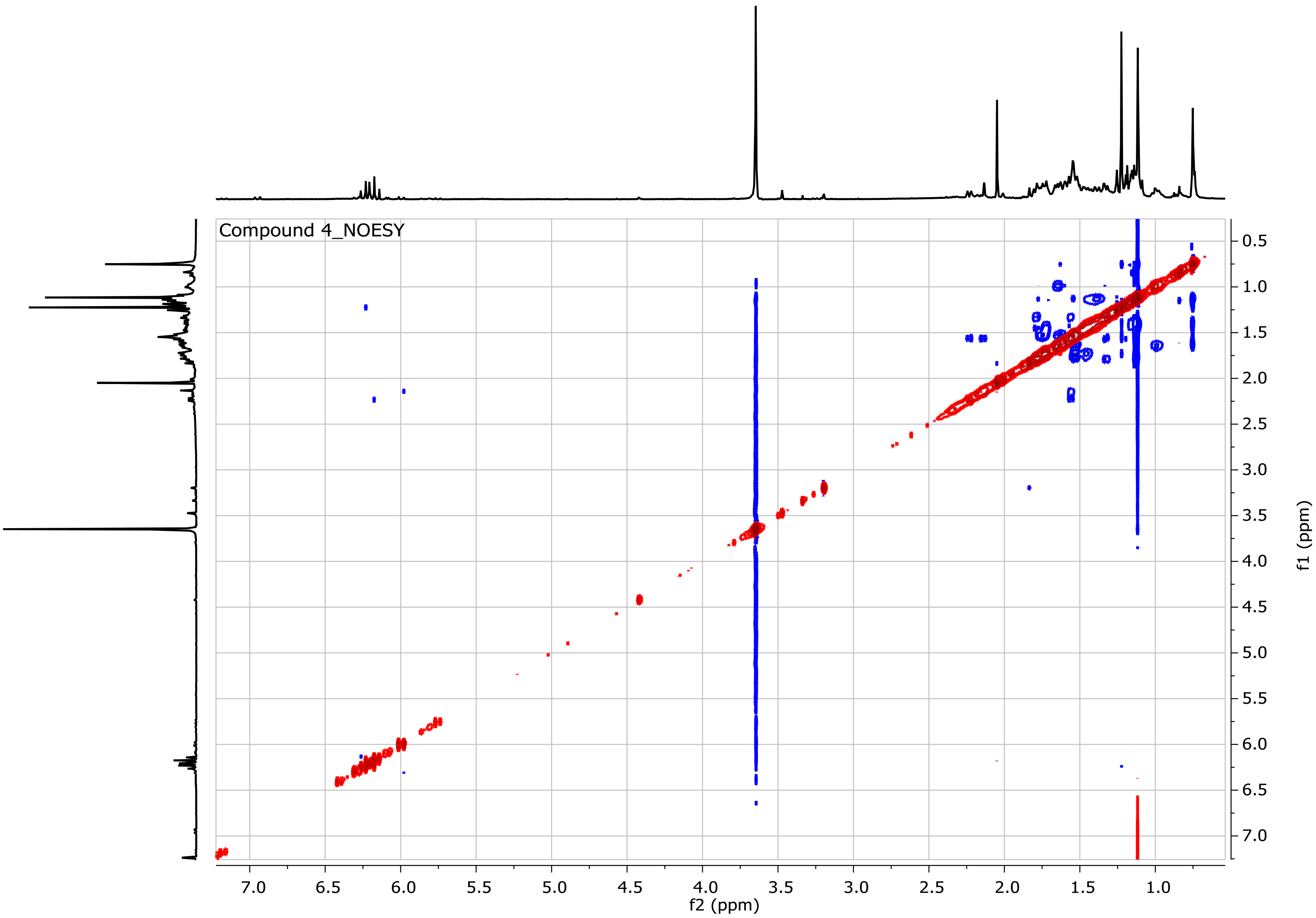

Figure S32. NOESY (500 MHz, $CDCl_3$) spectrum of compound **4**.

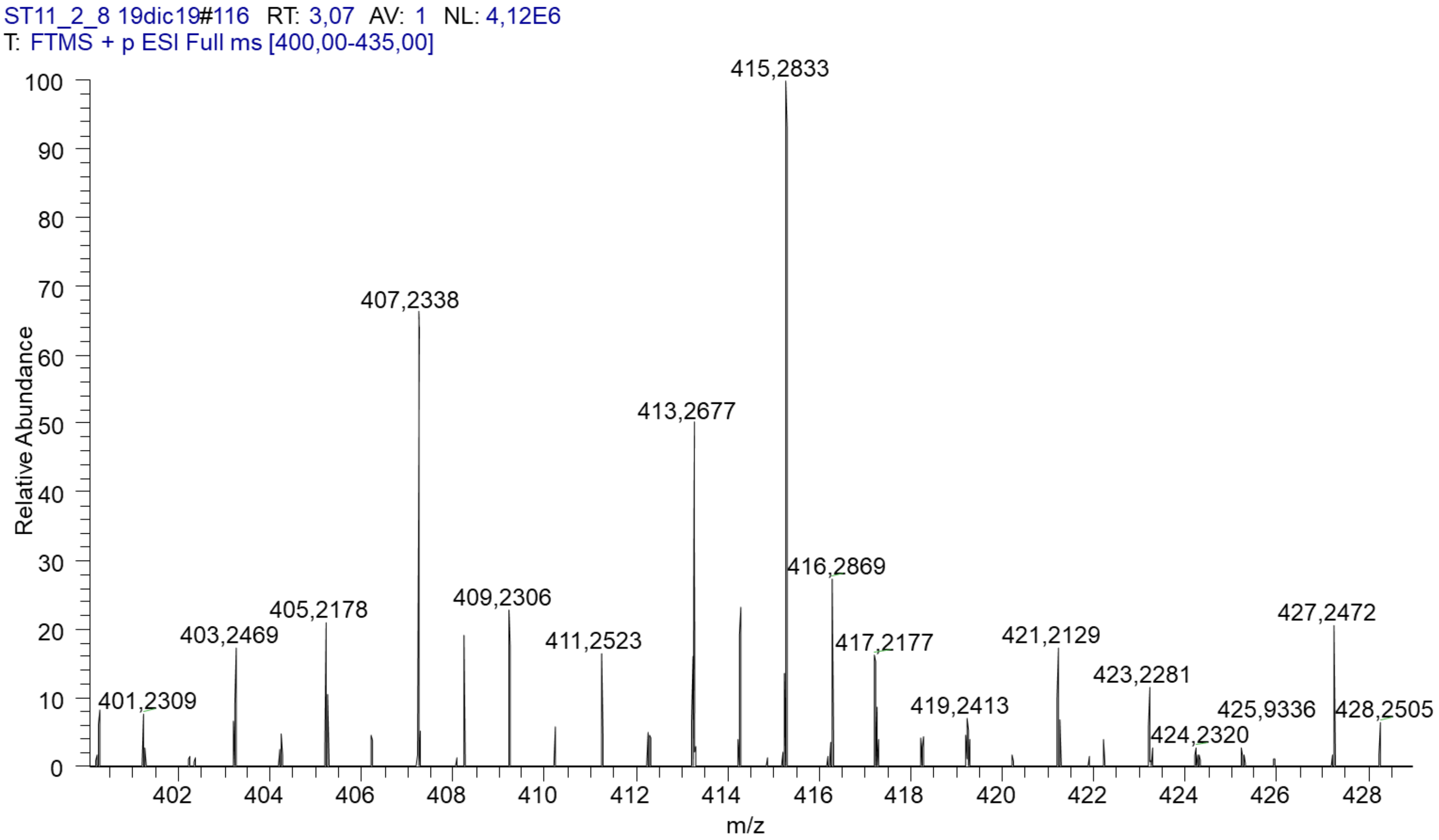

Figure S33. HRESIMS spectrum of compound **4**.

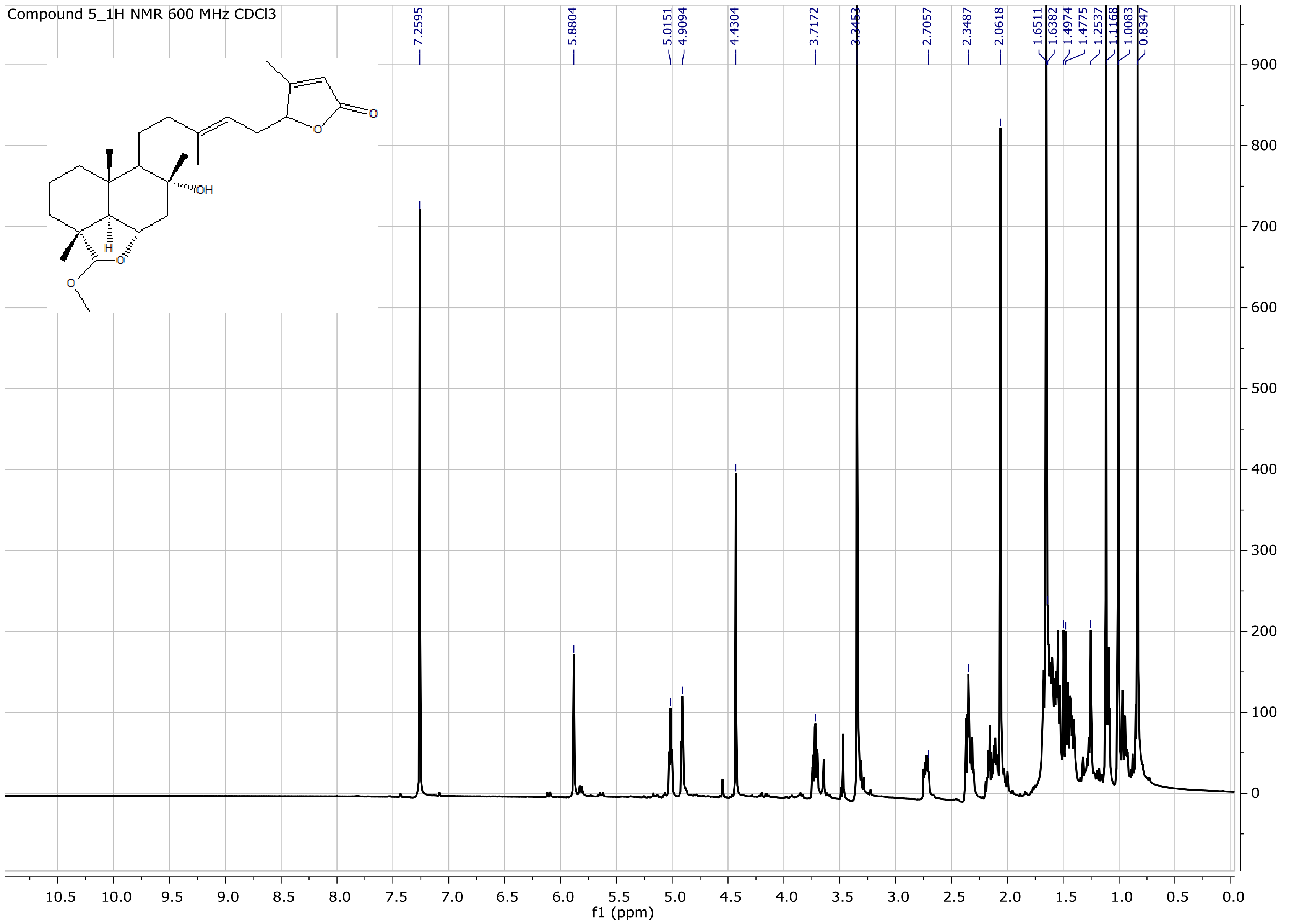

Figure S34. ^{1}H NMR (600 MHz, $CDCl_3$) spectrum of compound **5**.

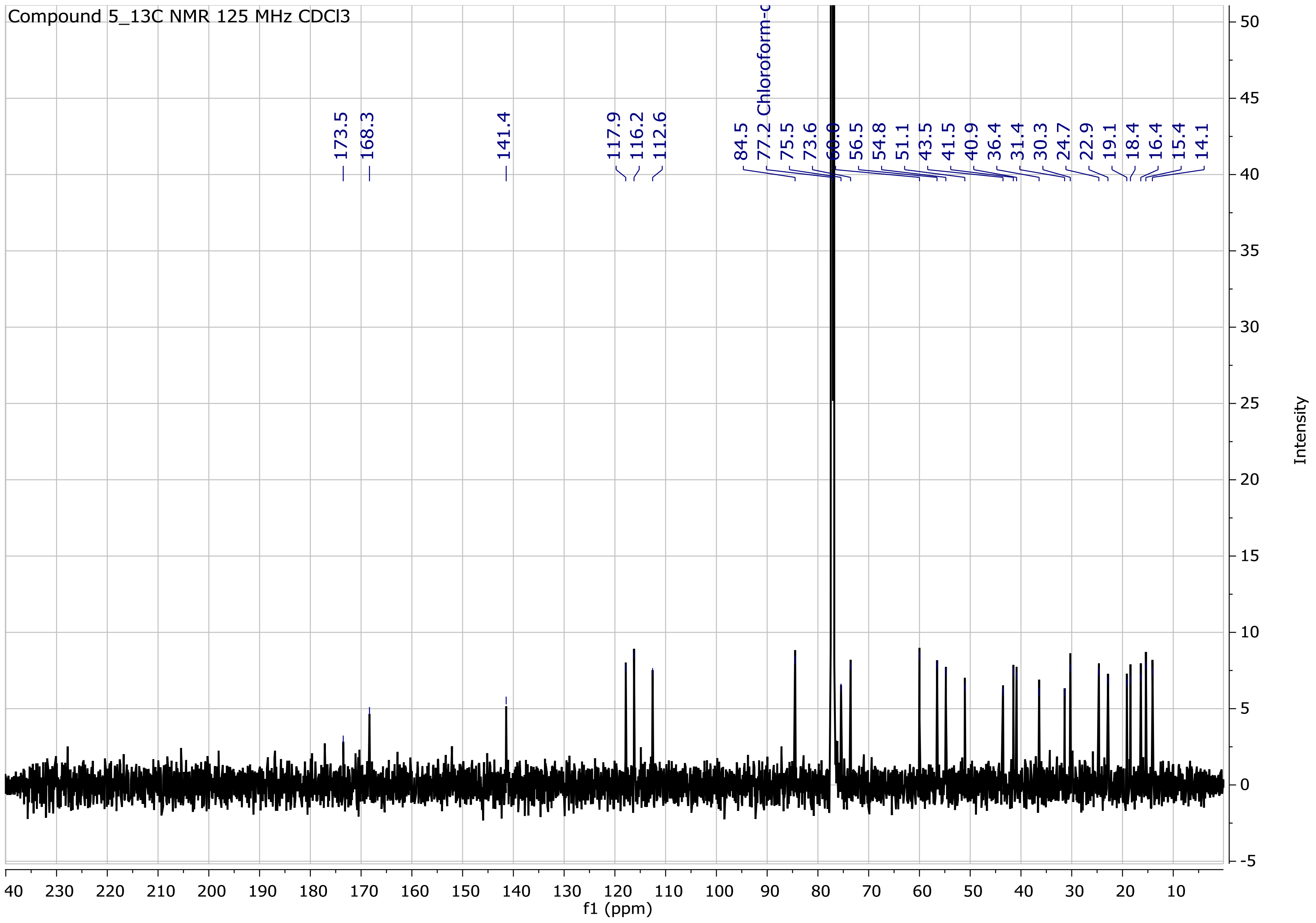

Figure S35. ^{13}C NMR (125 MHz, $CDCl_3$) spectrum of compound **5**.

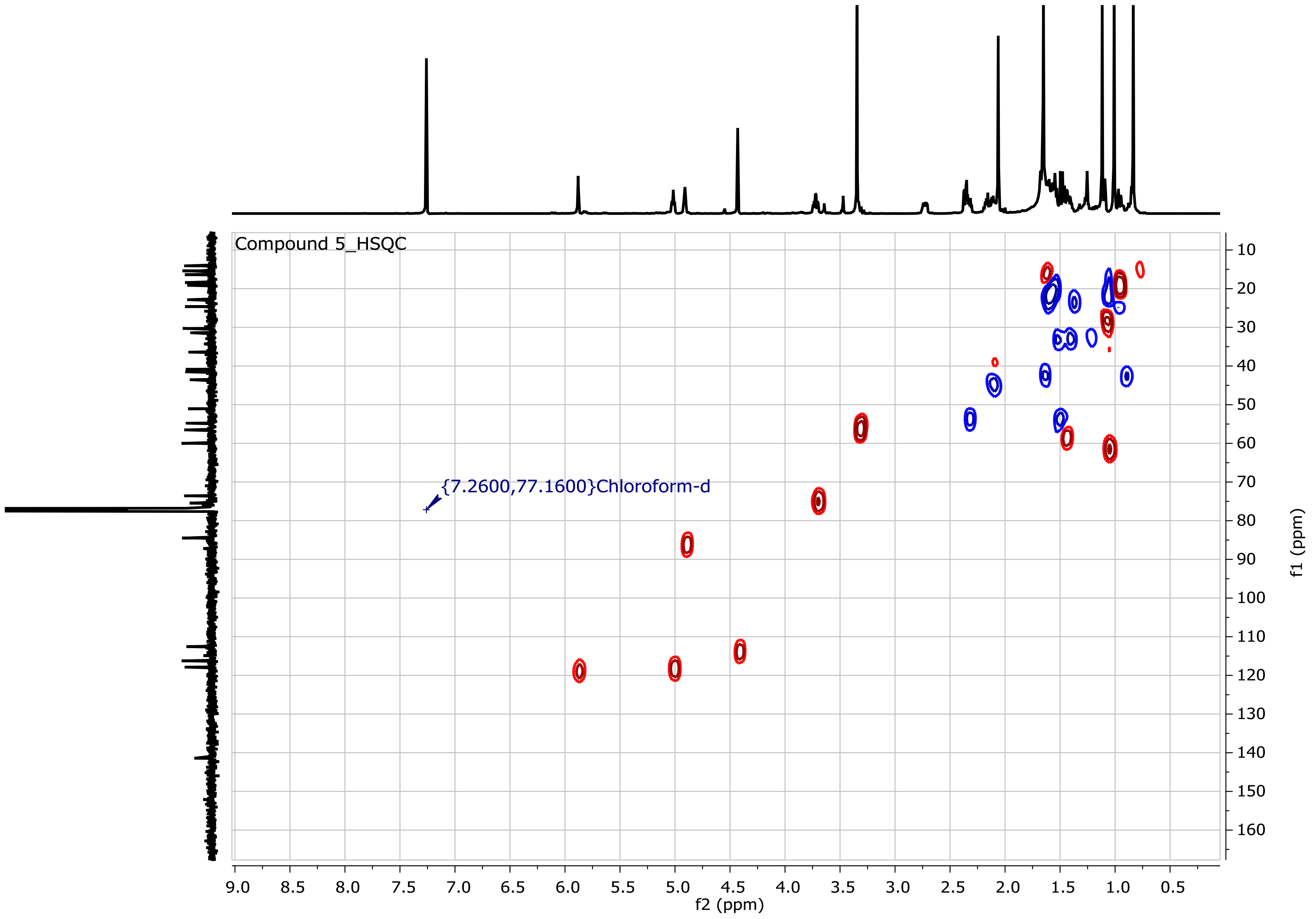

Figure S36. HSQC (600 MHz, $CDCl_3$) spectrum of compound **5**.

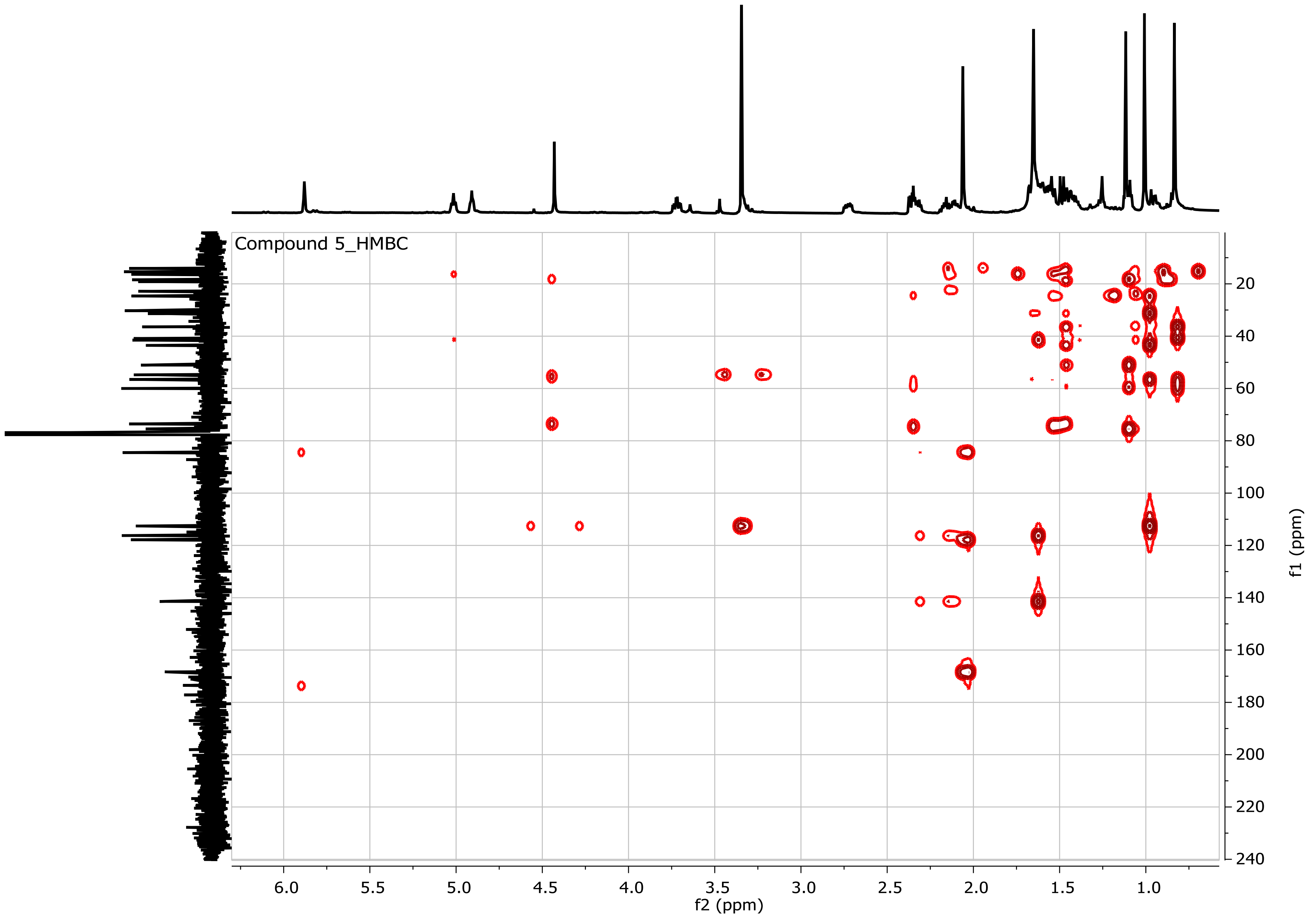

Figure S37. HMBC (600 MHz, $CDCl_3$) spectrum of compound **5**.

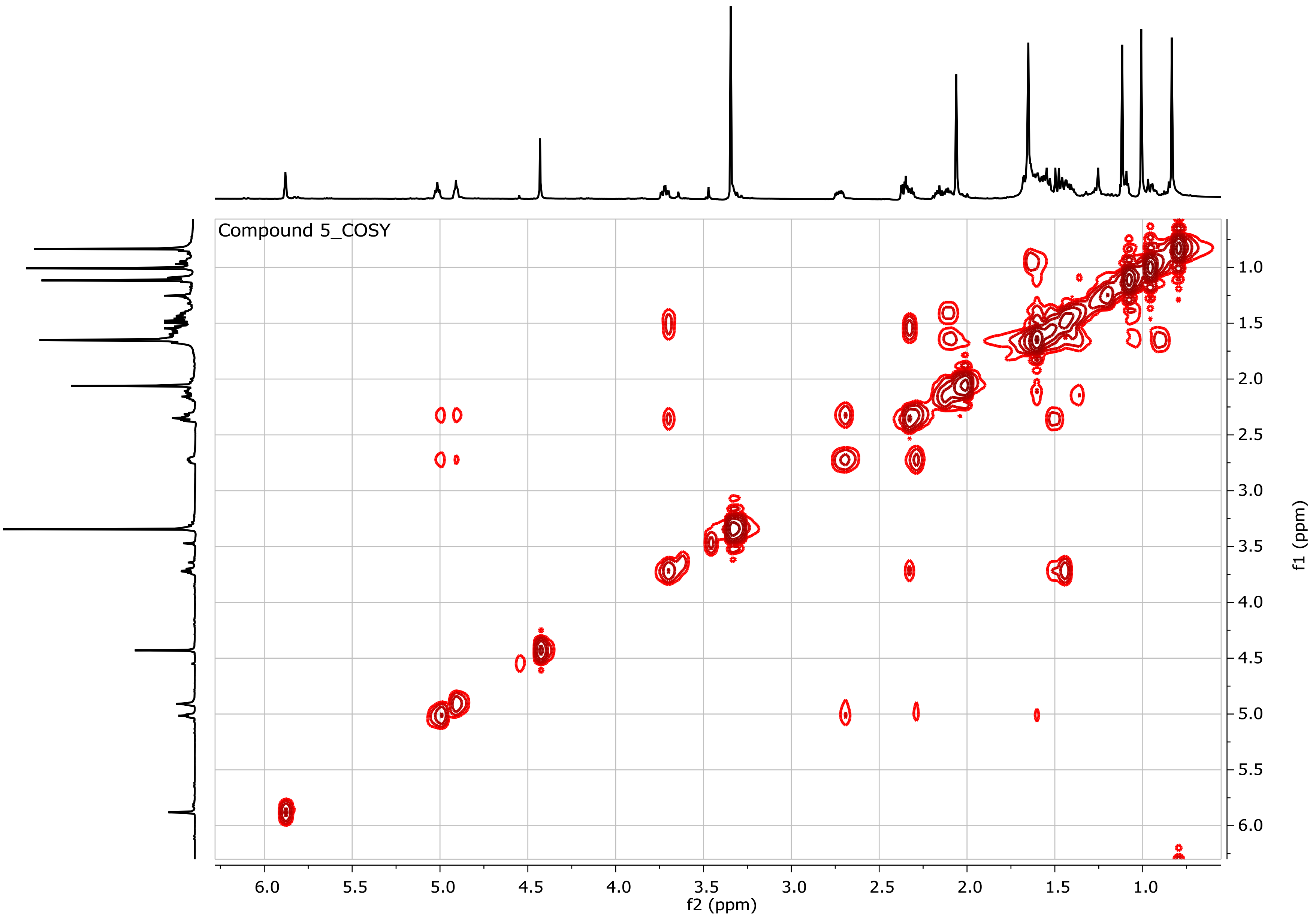

Figure S38. COSY (600 MHz, $CDCl_3$) spectrum of compound **5**.

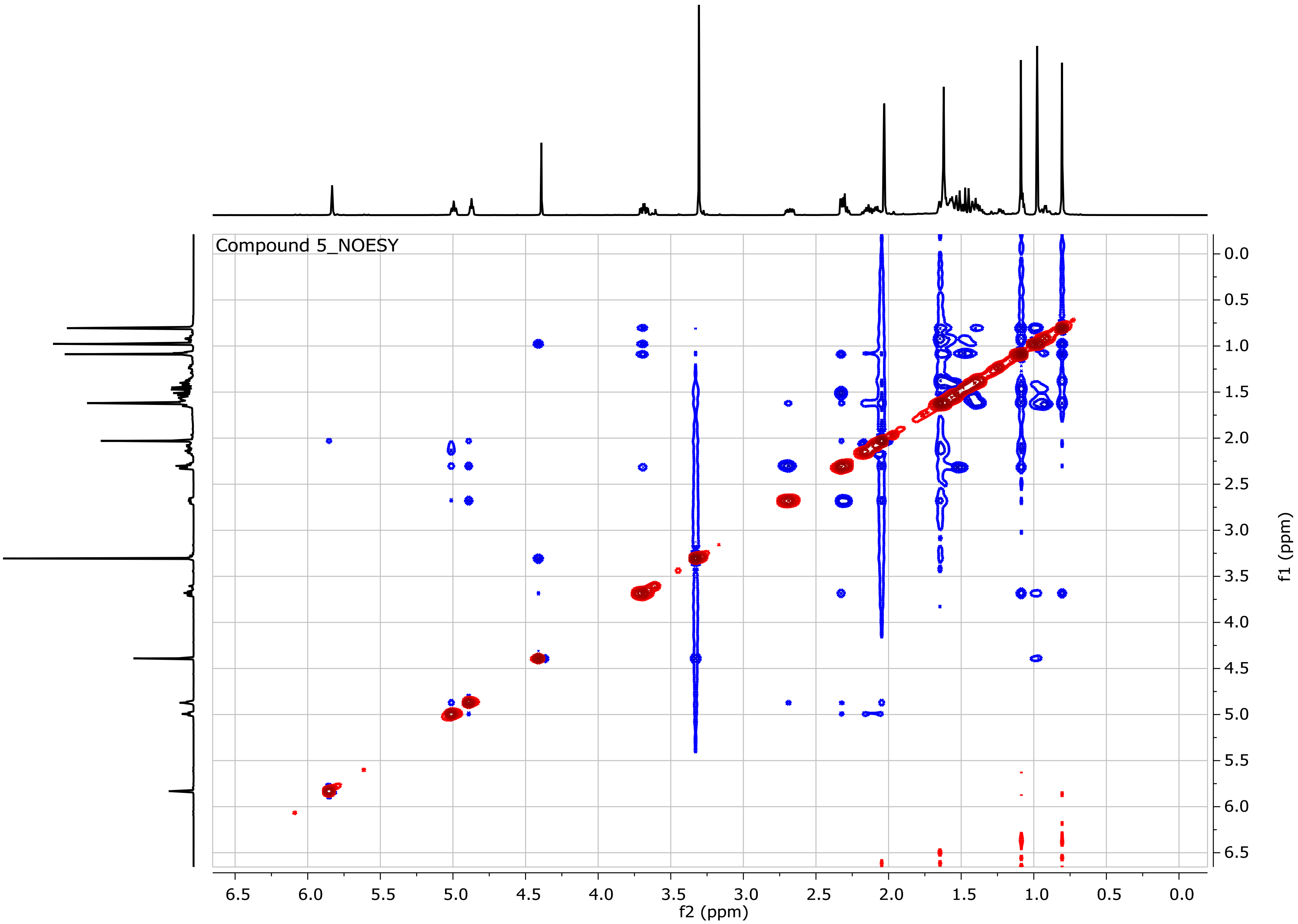

Figure S39. NOESY (500 MHz, $CDCl_3$) spectrum of compound **5**.

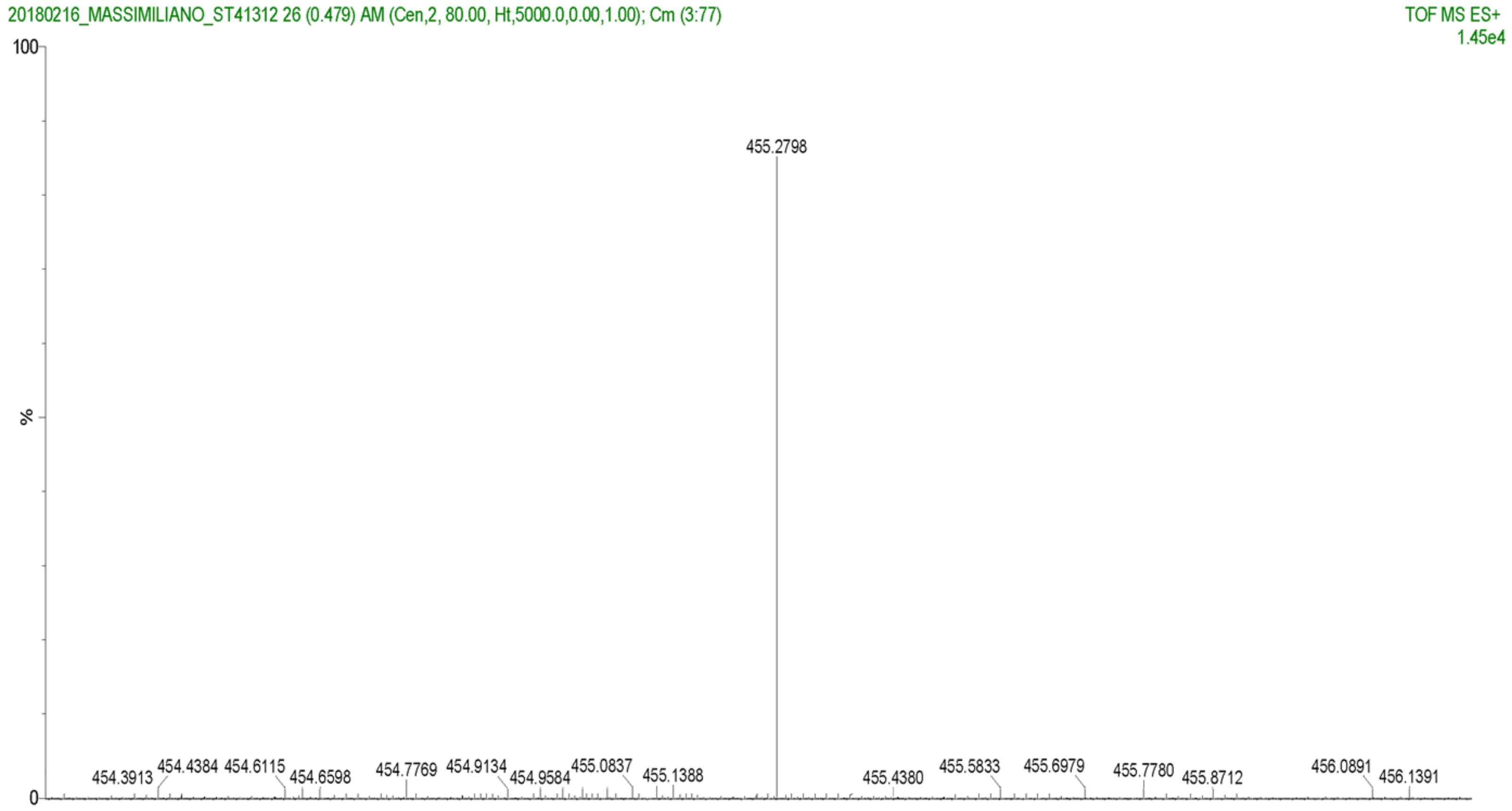

Figure S40. HRESIMS spectrum of compound **5**.

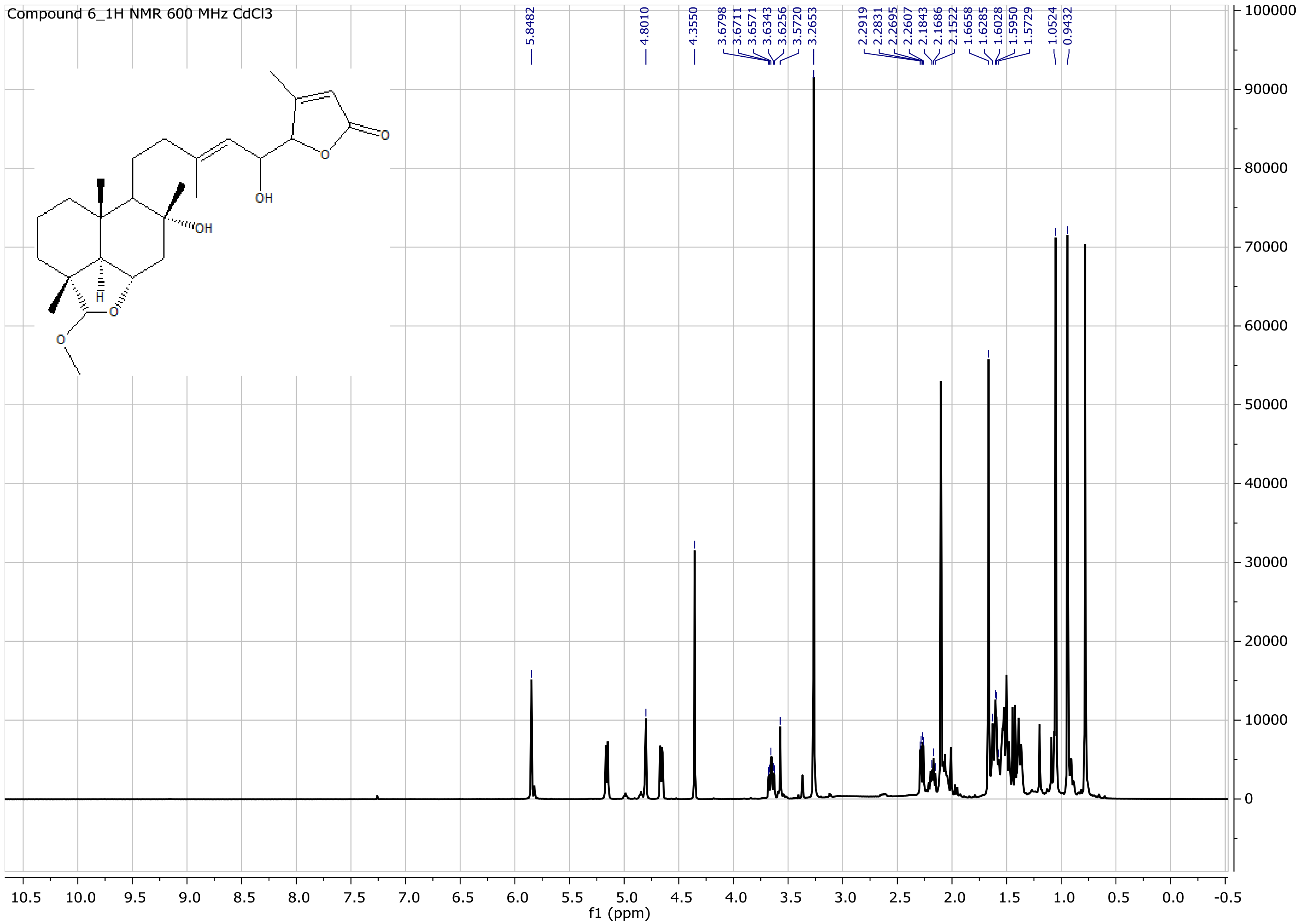

Figure S41. ^{1}H NMR (600 MHz, $CDCl_3$) spectrum of compound **6**.

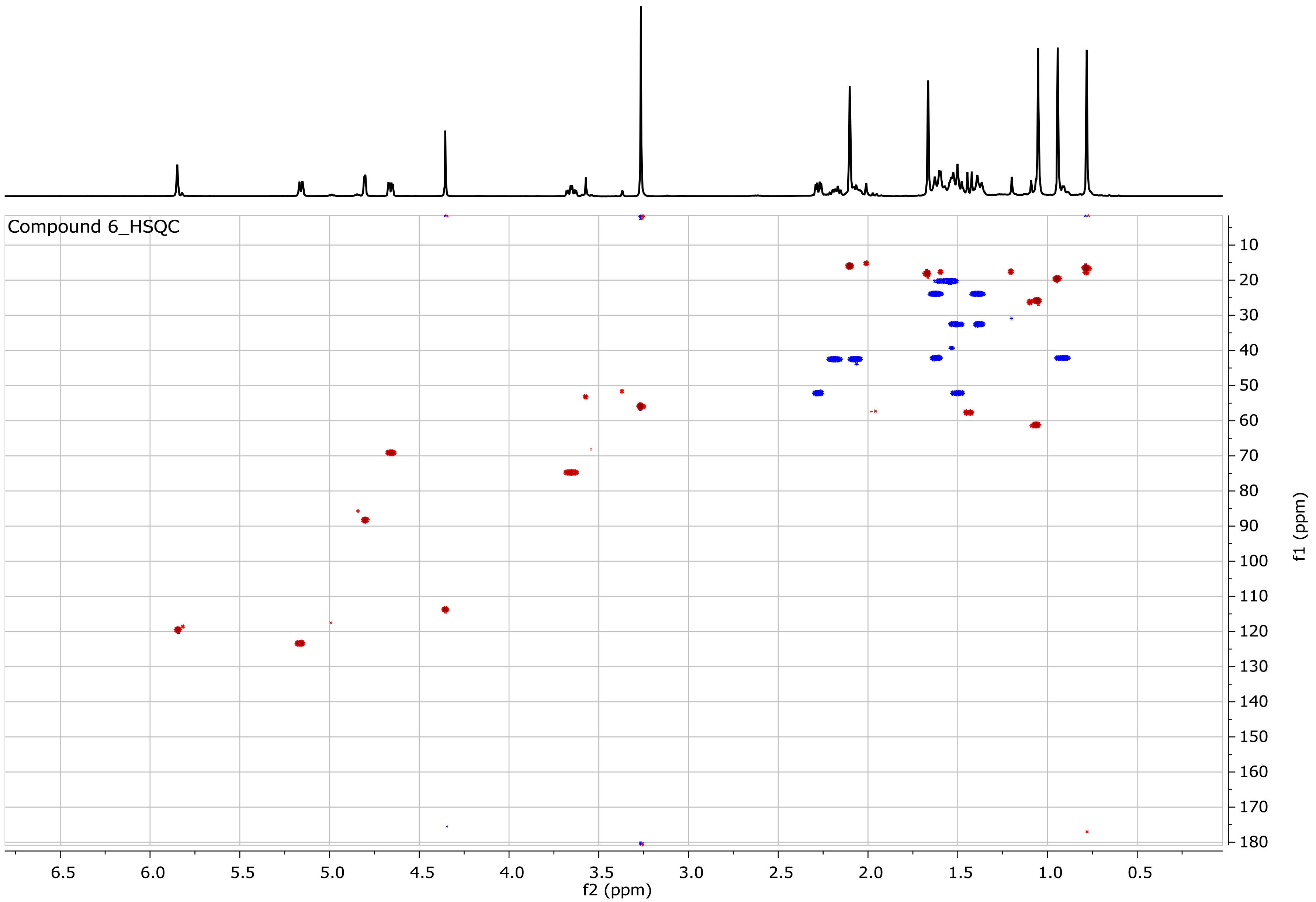

Figure S42. HSQC (600 MHz, $CDCl_3$) spectrum of compound **6**.

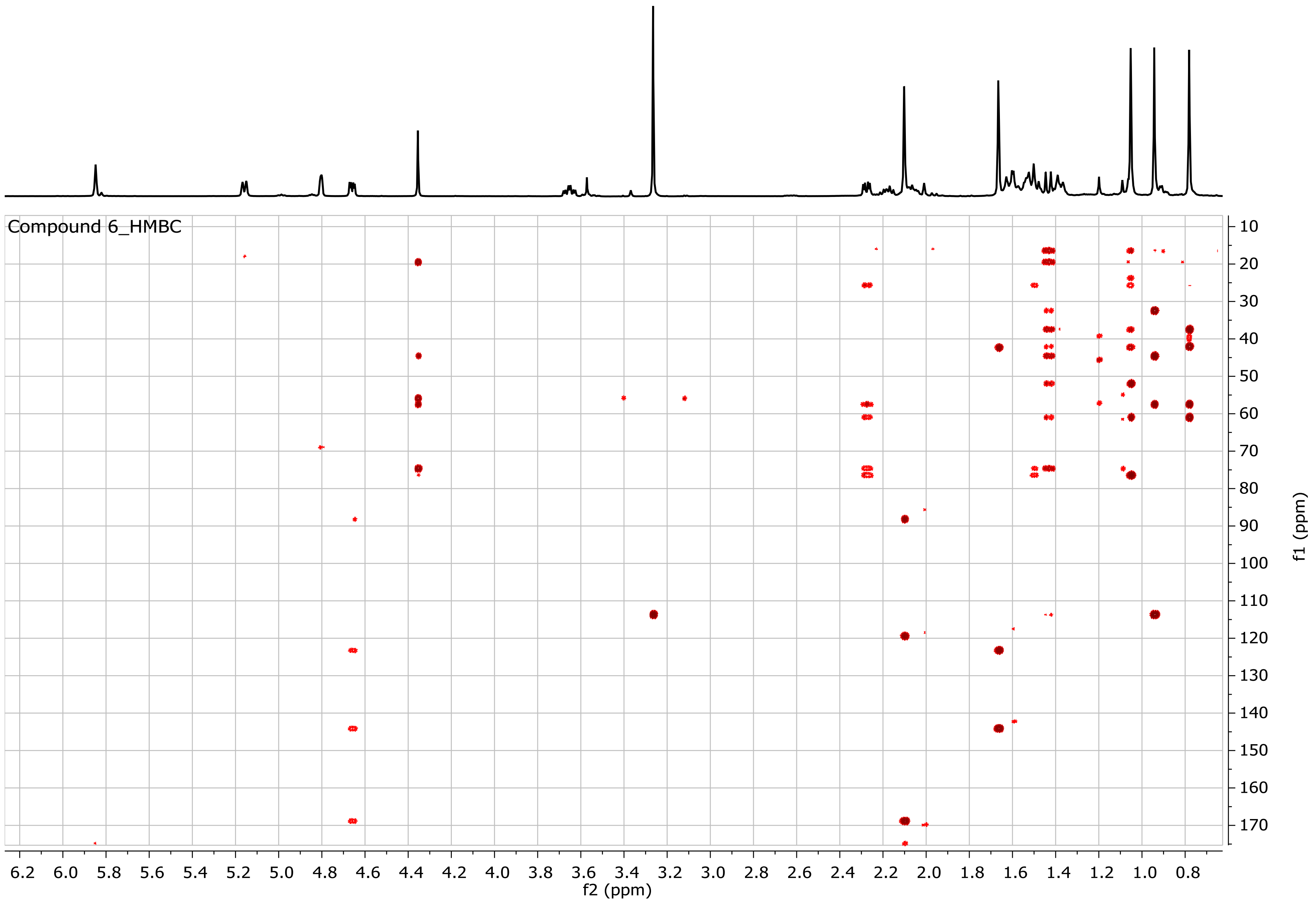

Figure S43. HMBC (600 MHz, $CDCl_3$) spectrum of compound **6**.

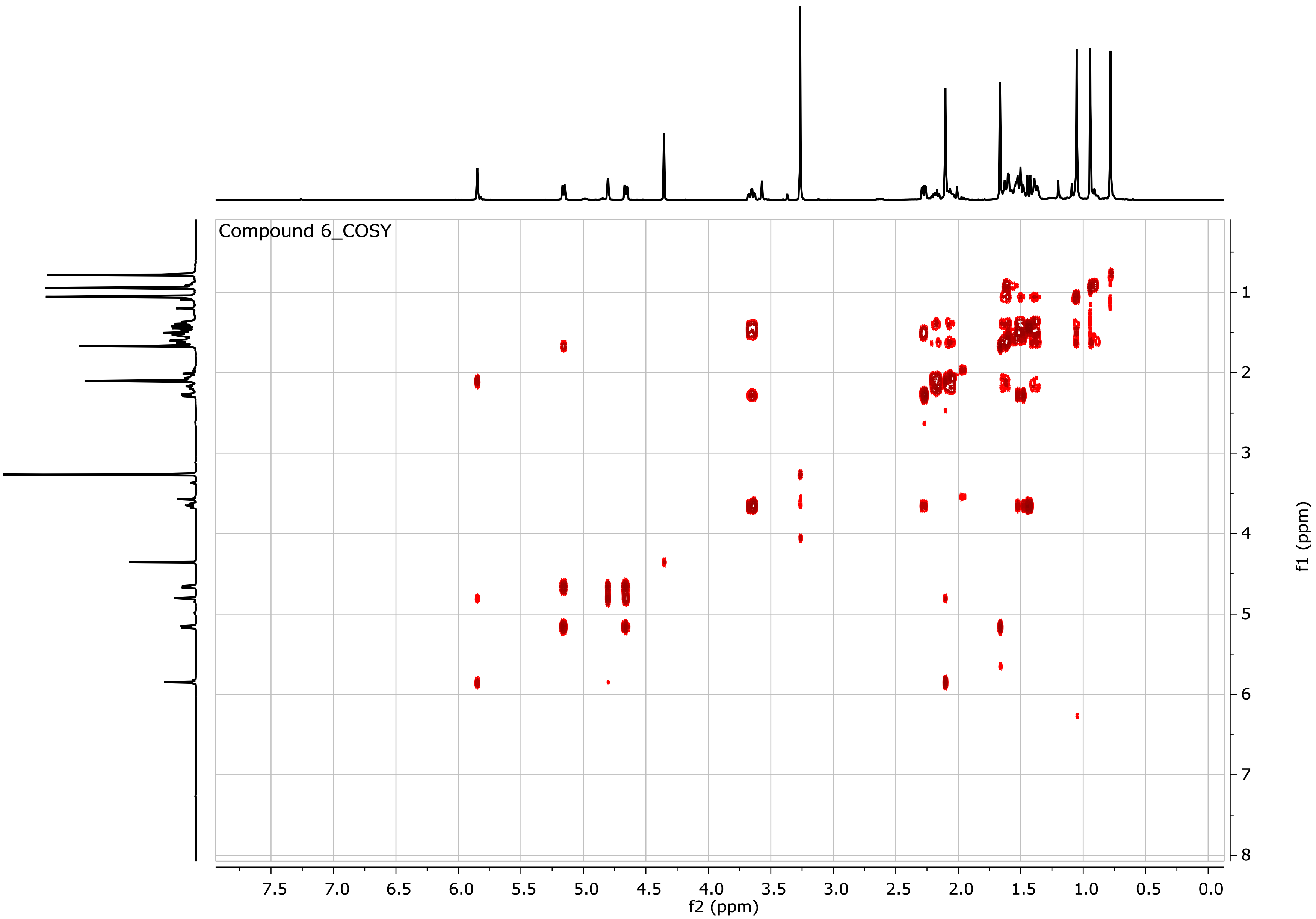

Figure S44. COSY (600 MHz, $CDCl_3$) spectrum of compound **6**.

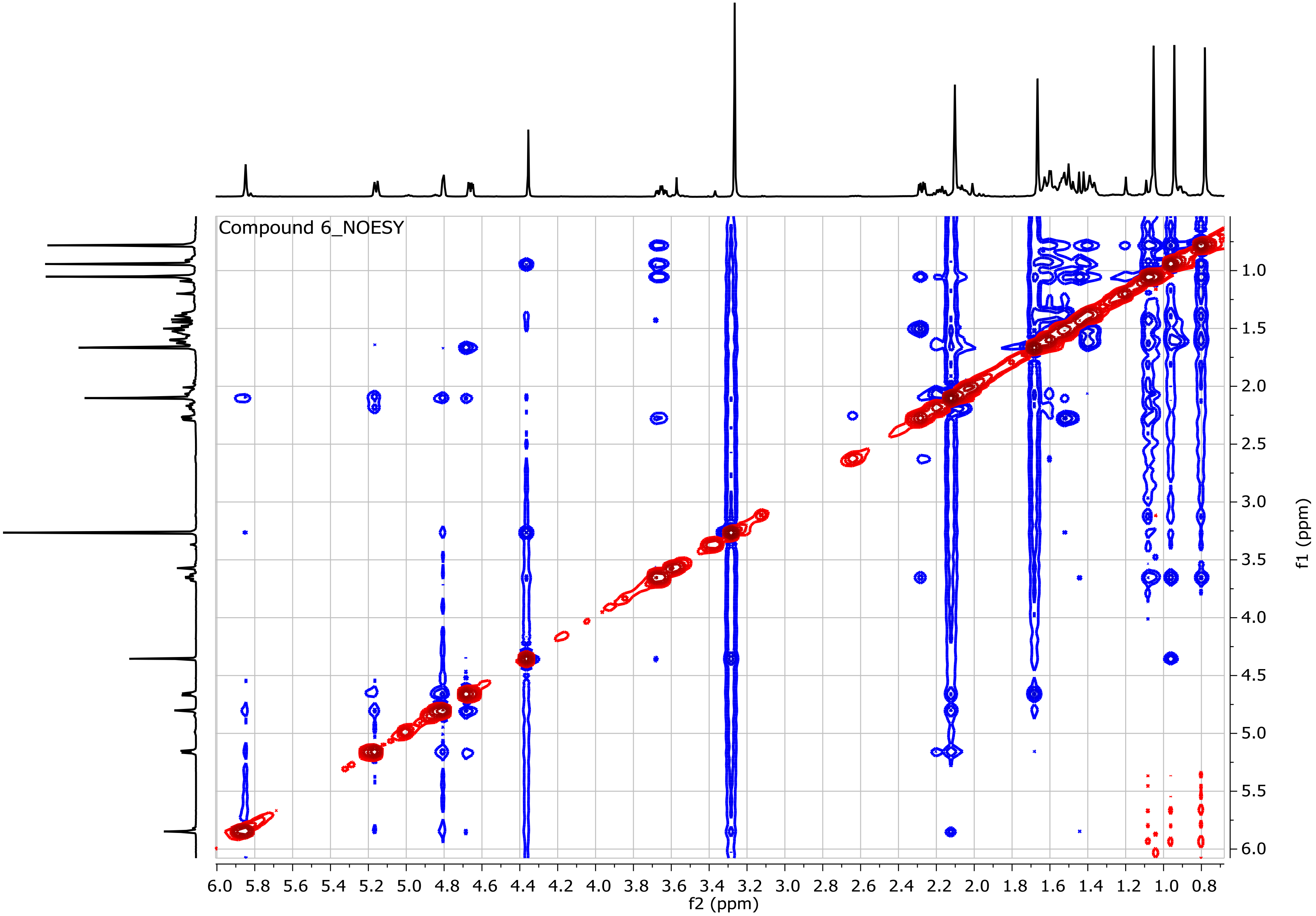

Figure S45. NOESY (500 MHz, $CDCl_3$) spectrum of compound **6**.

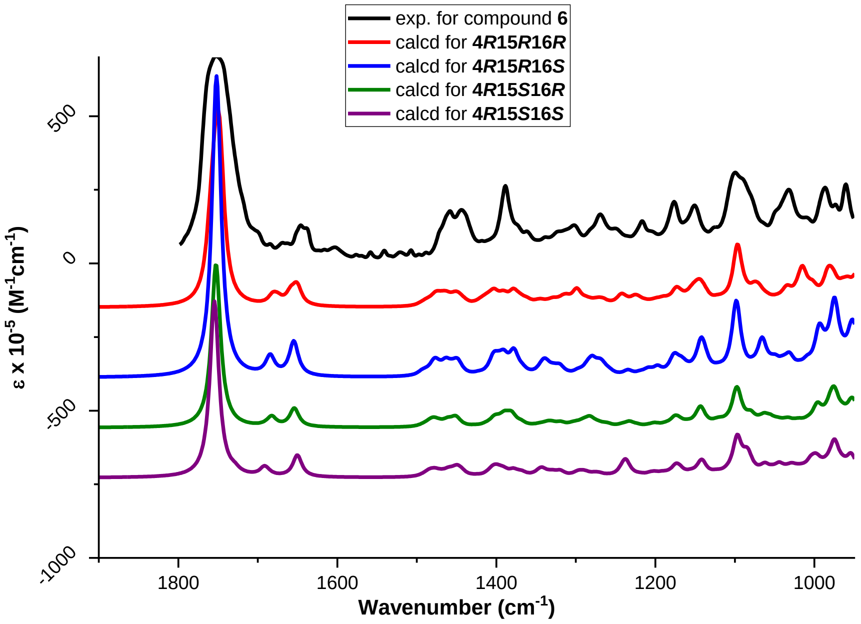

Figure S46. Comparison of experimental and computed IR spectra ($CDCl_3$) for compound **6.**
4*R* stands for 4*R*,5*R*,6*S*,8*R*,9*R*,10*S*,23*S*. The wavenumber scale factor 0.9820 was used to scale the computed spectra.

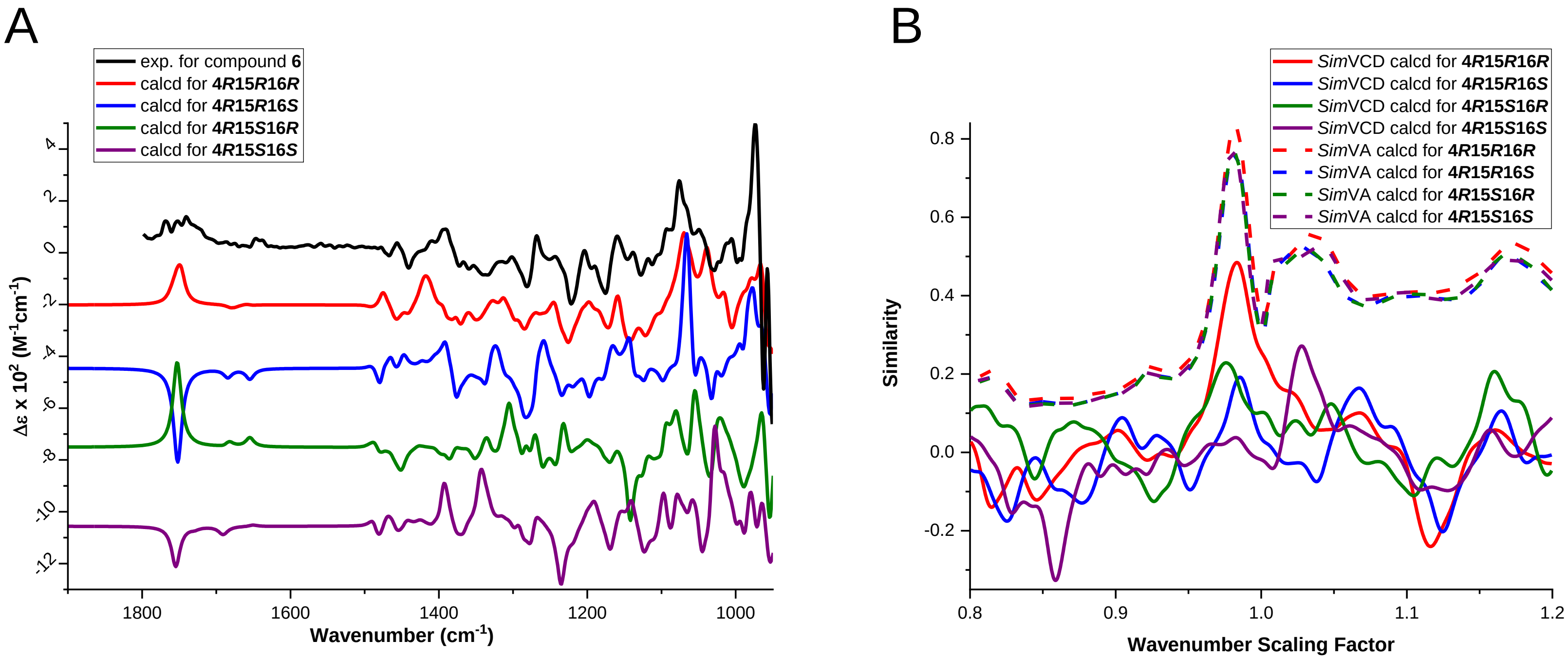

Figure S47. Comparison of experimental and computed VCD spectra in $CDCl_3$ for compound **6** (A). Similarities (*Sim*VA and *Sim*VCD) between experimental and computed VA and VCD spectra of **6** were plotted as functions of wavenumber scale factor (B). 4*R* stands for 4*R*,5*R*,6*S*,8*R*,9*R*,10*S*,23*S*. The wavenumber scale factor corresponding to the maximal *Sim*VA value in B (0.9820) was used to scale the computed spectra in A.

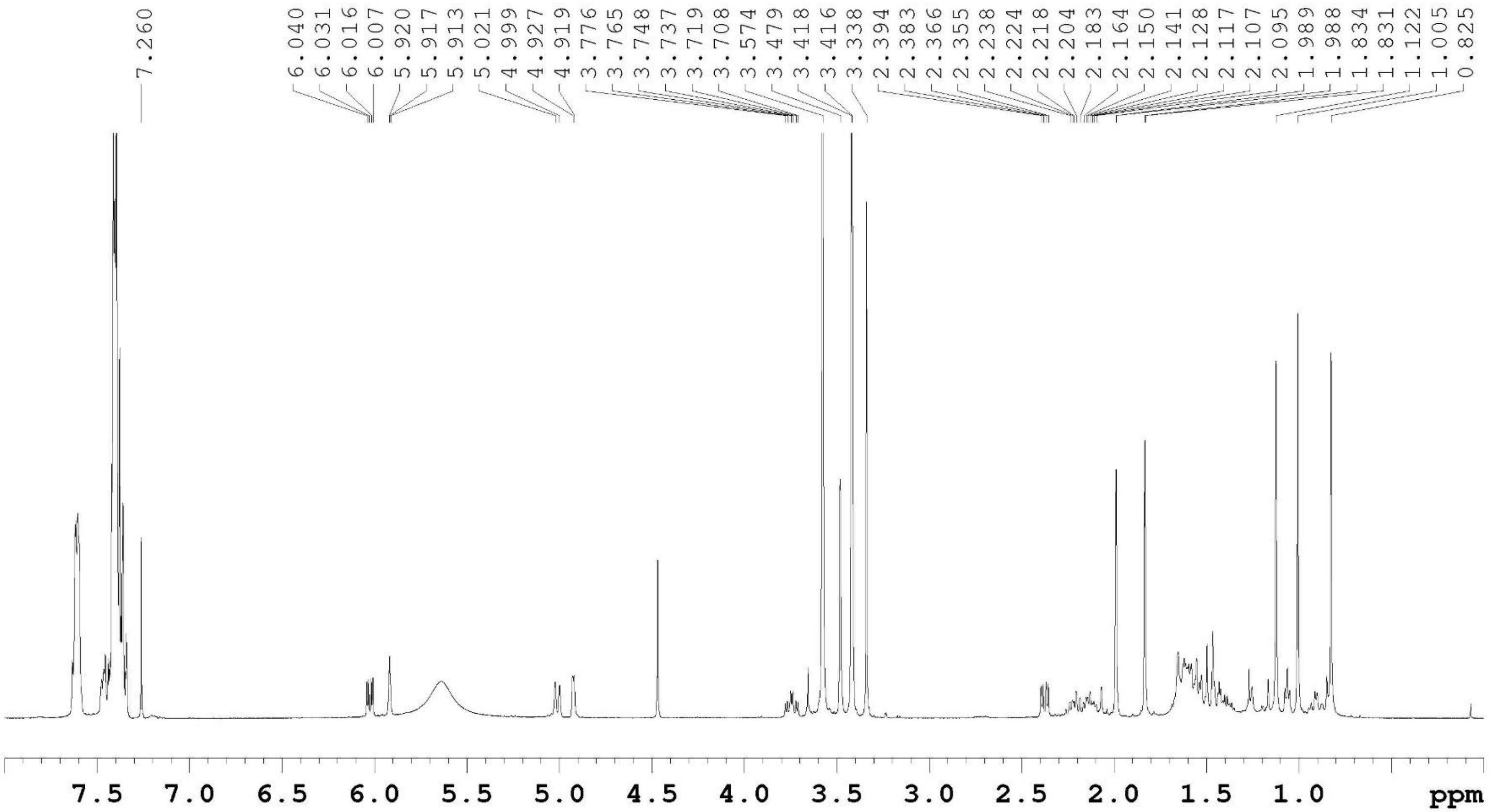

Figure S48. ^{1}H NMR (400 MHz, $CDCl_3$) spectrum of S-MTPA ester of compound **6** (by esterification of **6** with (R)-(-)-MTPA-Cl).

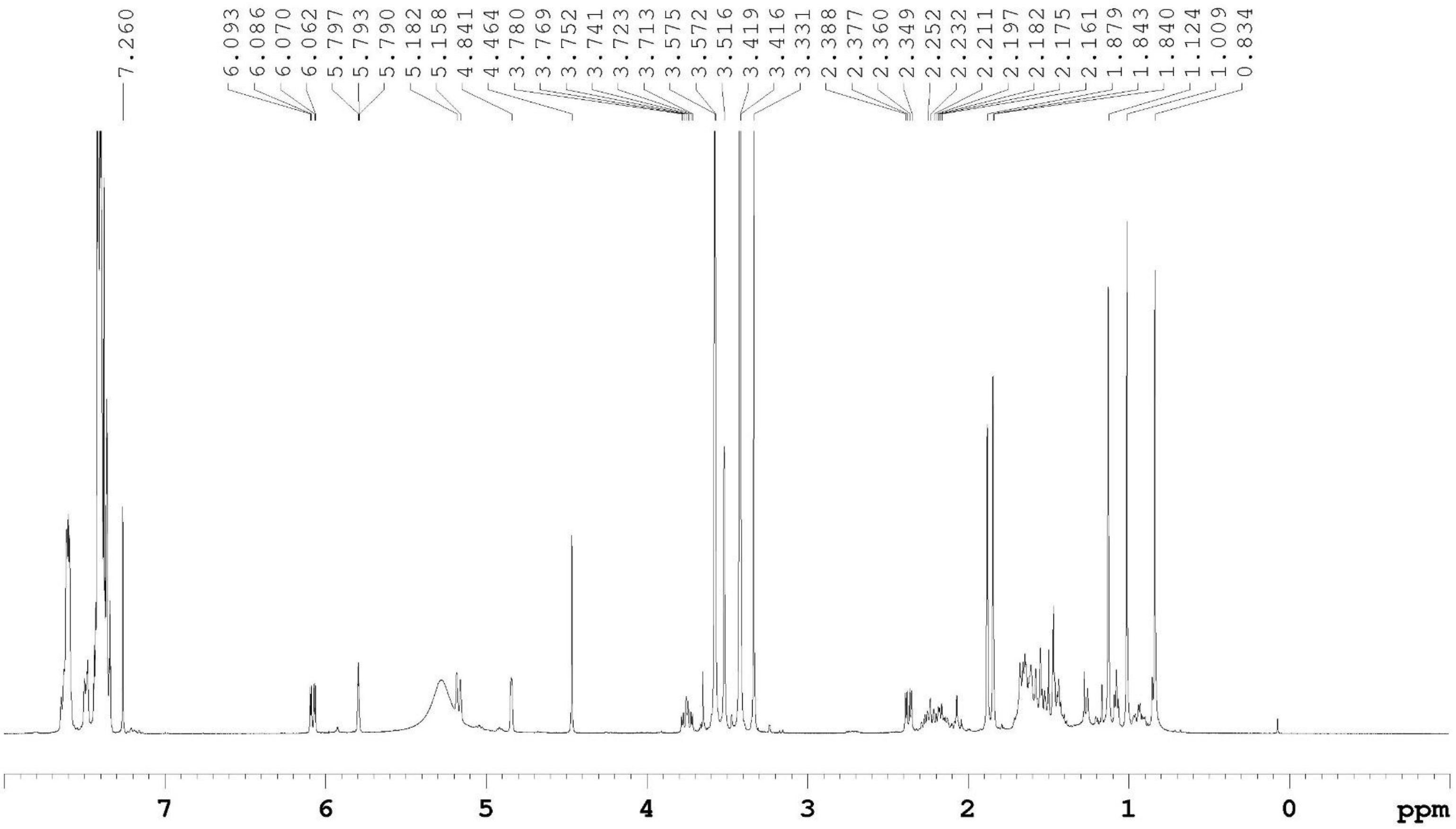

Figure S49. ^{1}H NMR (400 MHz, $CDCl_3$) spectrum of R-MTPA ester of compound **6** (by esterification of **6** with (S)-(+)-MTPA-Cl).

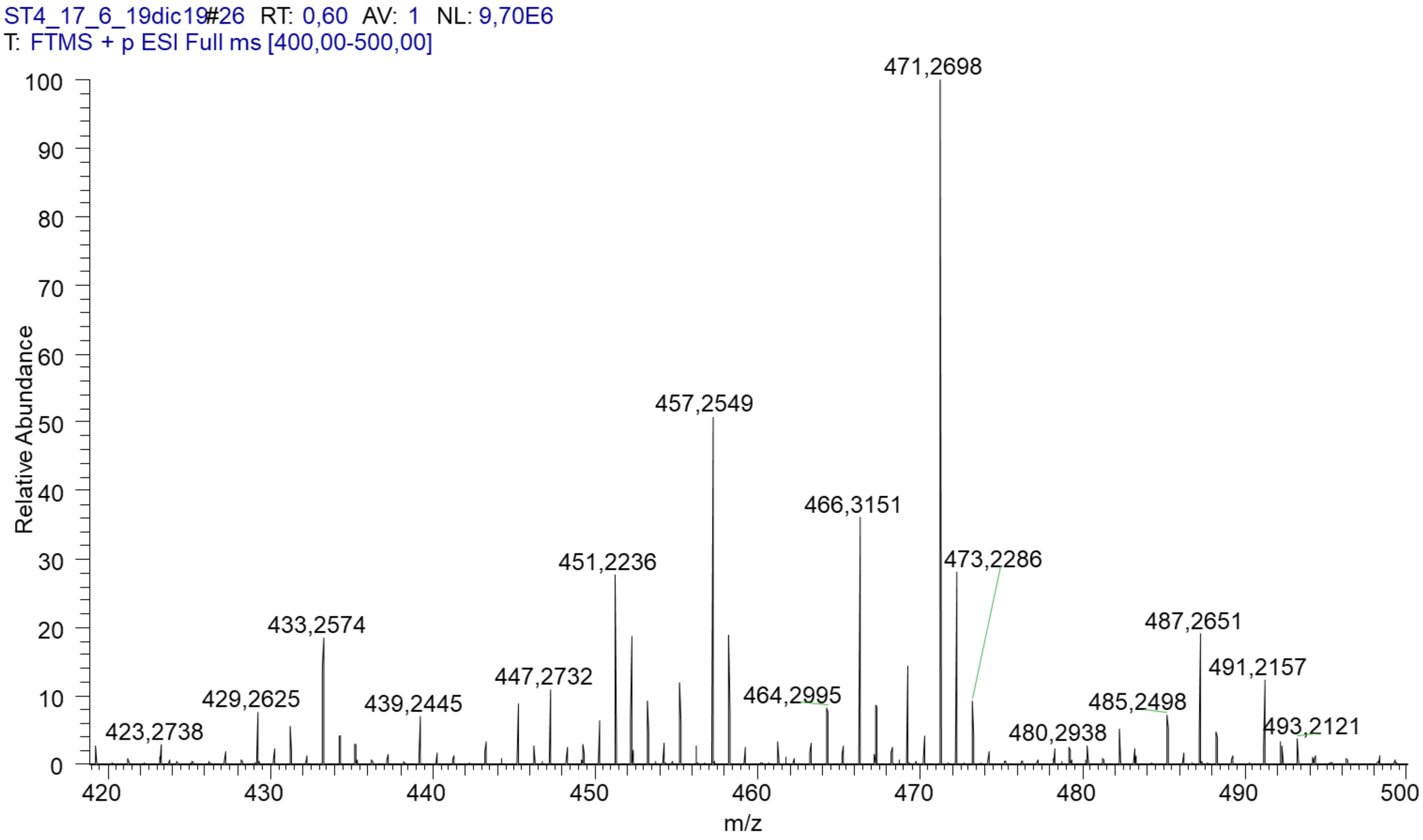

Figure S50. HRESIMS spectrum of compound **6**.

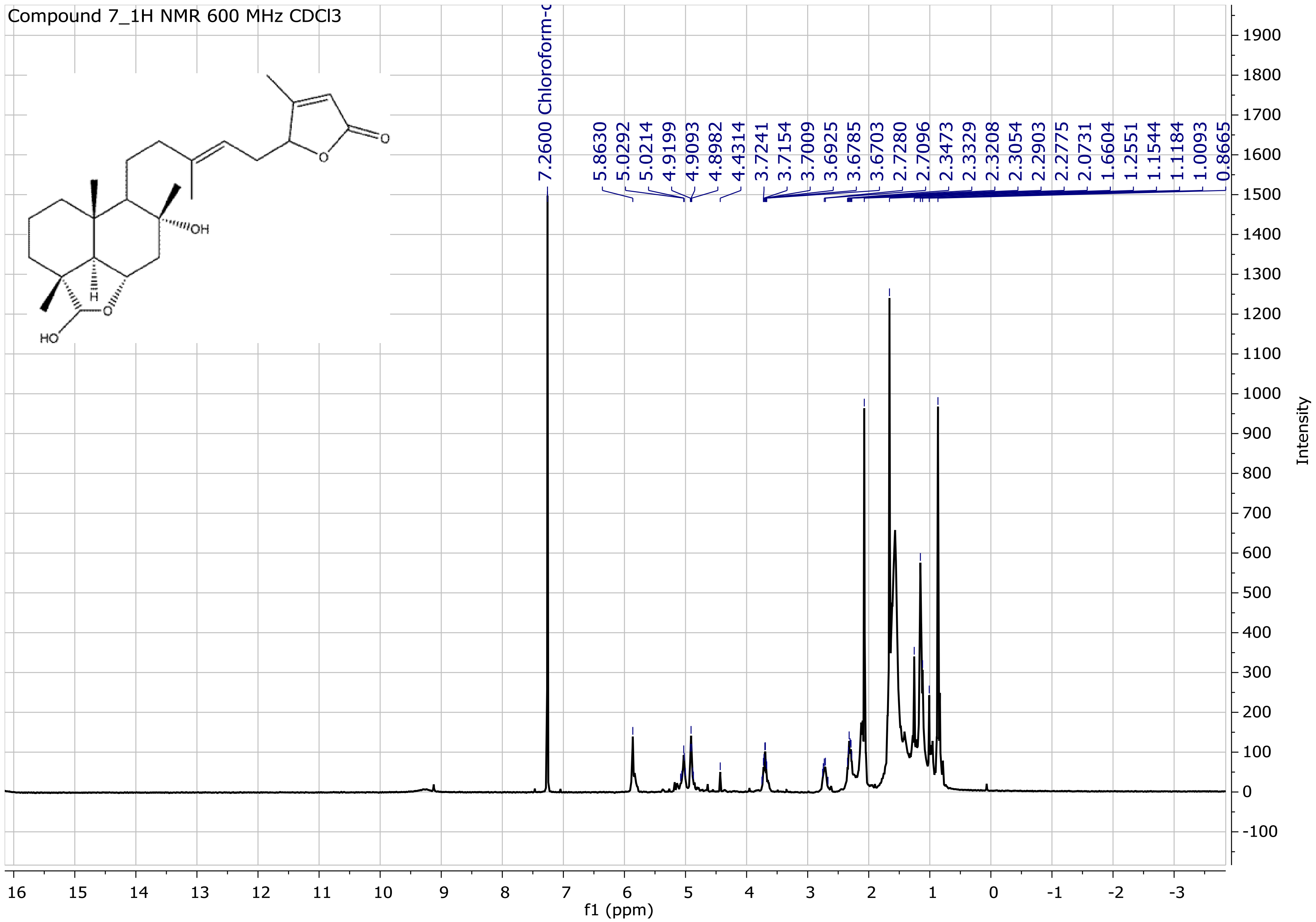

Figure S51. ^{1}H NMR (600 MHz, $CDCl_3$) spectrum of compound **7**.

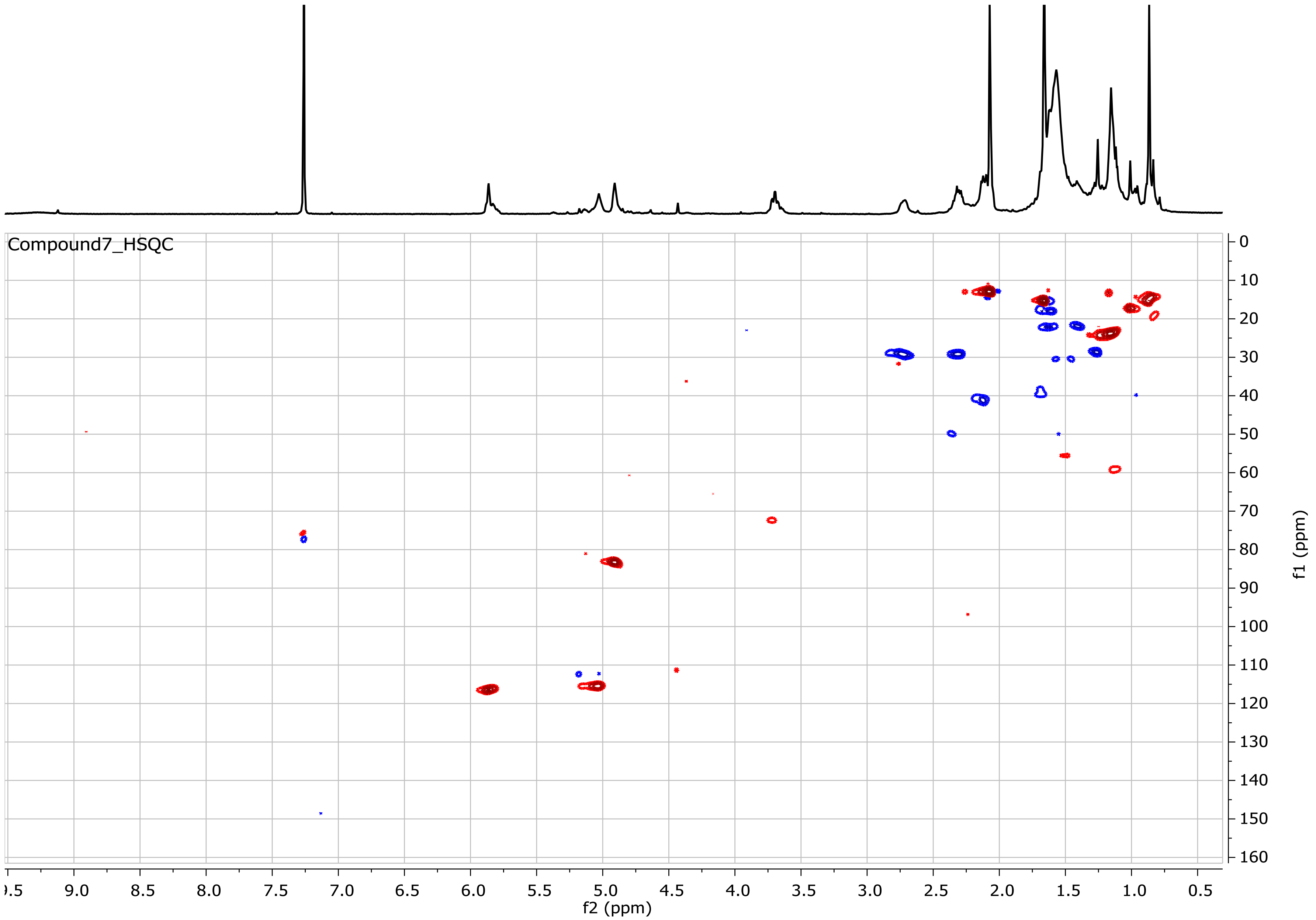

Figure S52. HSQC (600 MHz, $CDCl_3$) spectrum of compound **7**.

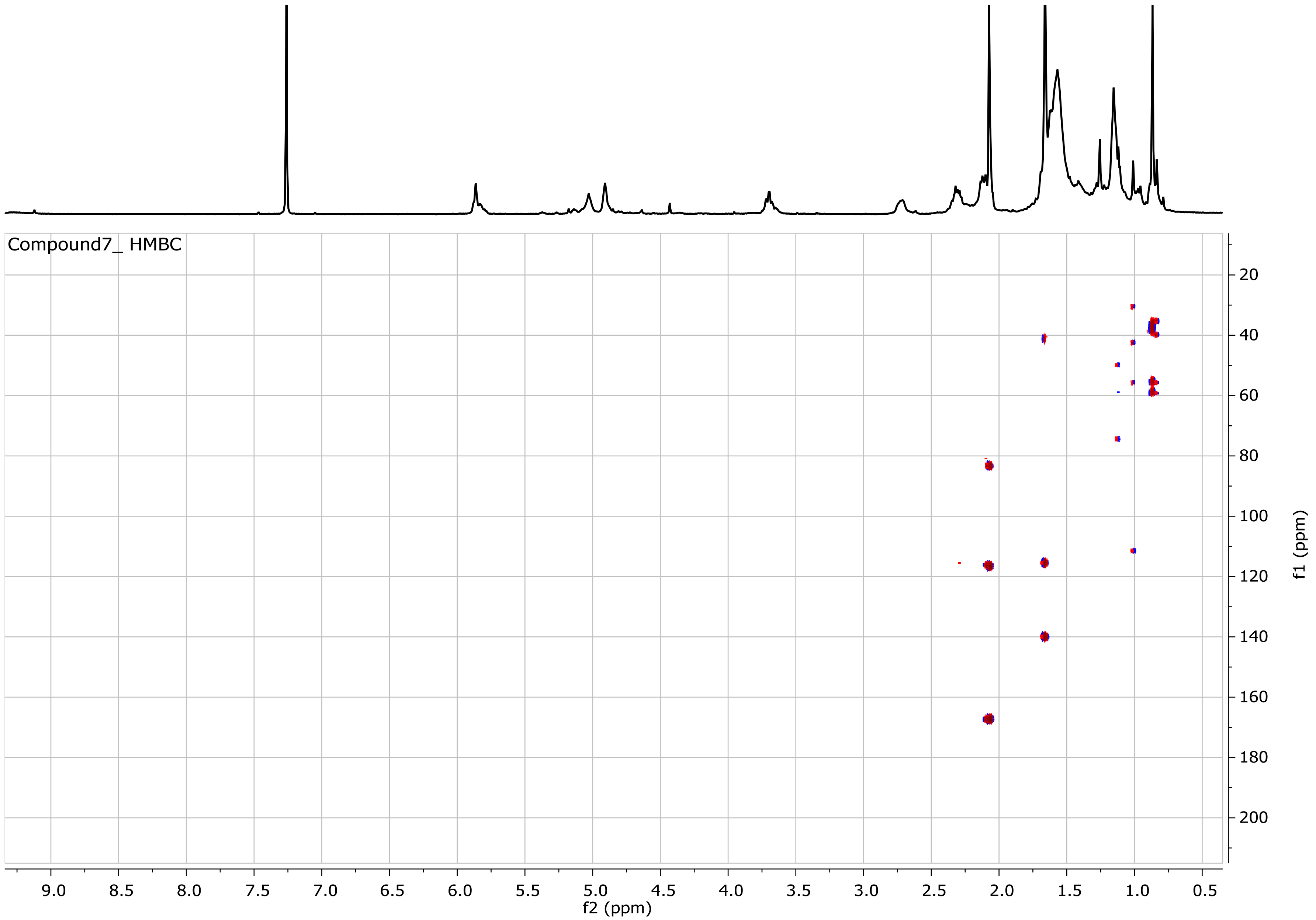

Figure S53. HMBC (600 MHz, $CDCl_3$) spectrum of compound **7**.

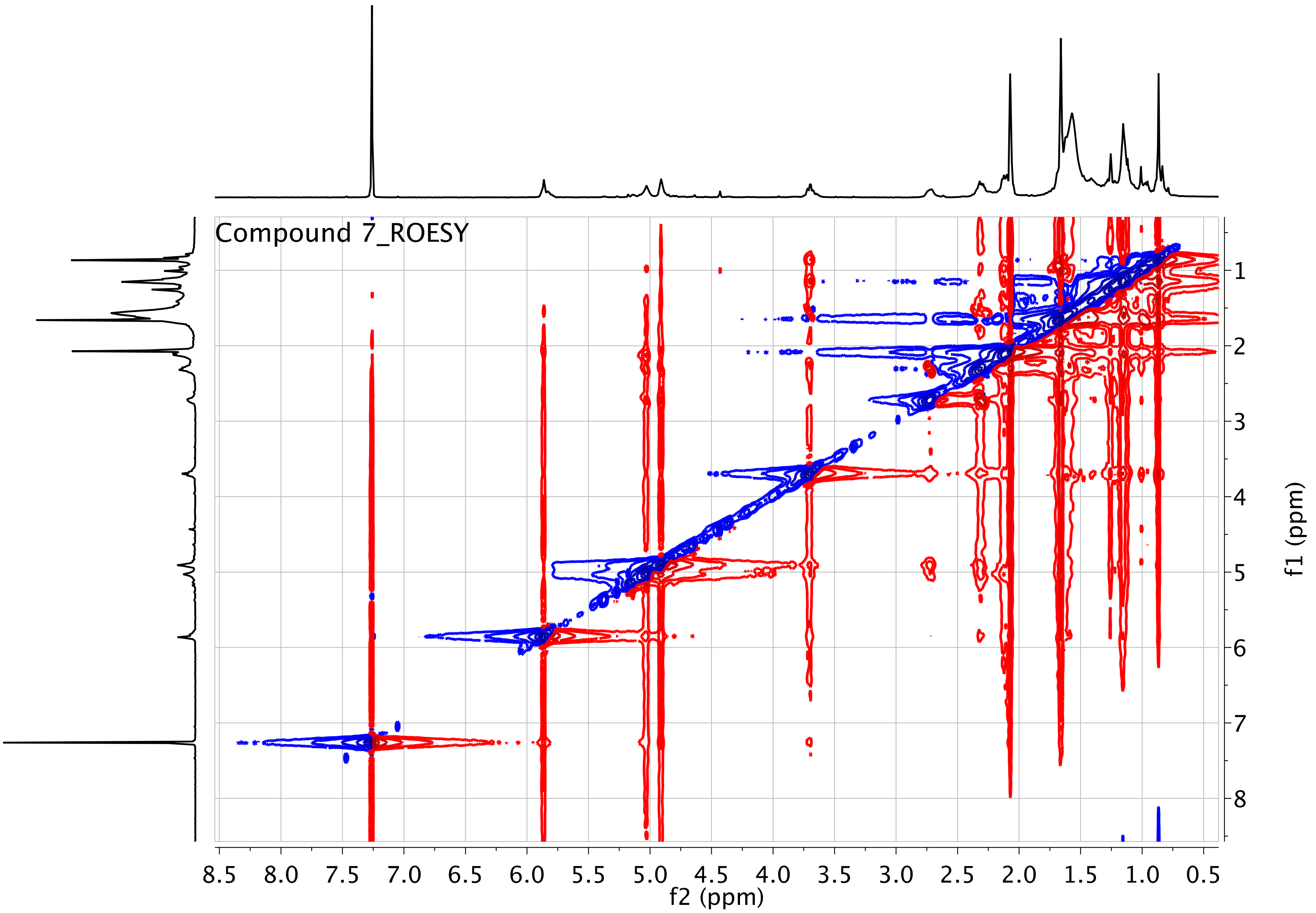

Figure S54. ROESY (600 MHz, $CDCl_3$) spectrum of compound **7**.

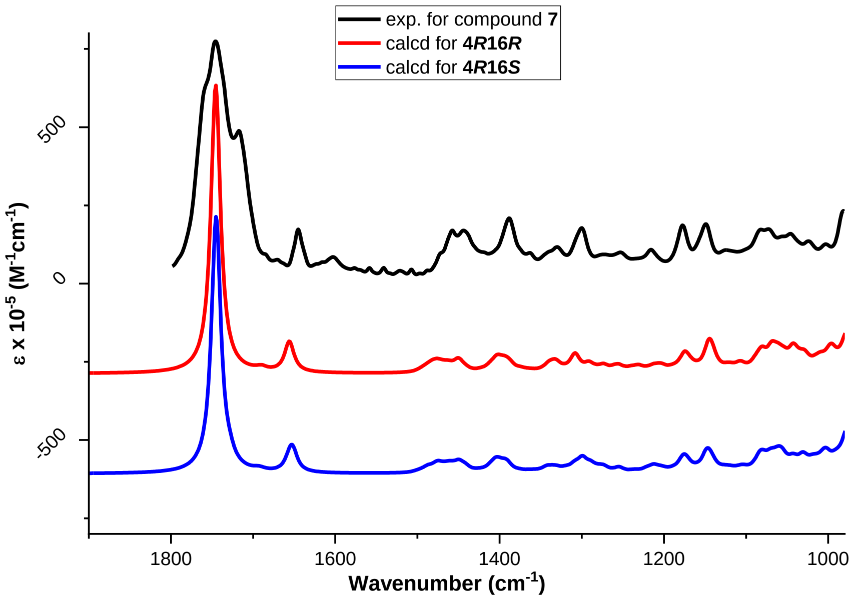

Figure S55. Comparison of experimental and computed IR spectra ($CDCl_3$) for compound **7**.
4*R* stands for 4*R*,5*R*,6*S*,8*R*,9*R*,10*S*,23*S*. The wavenumber scale factor 0.9820 was used to scale the computed spectra.

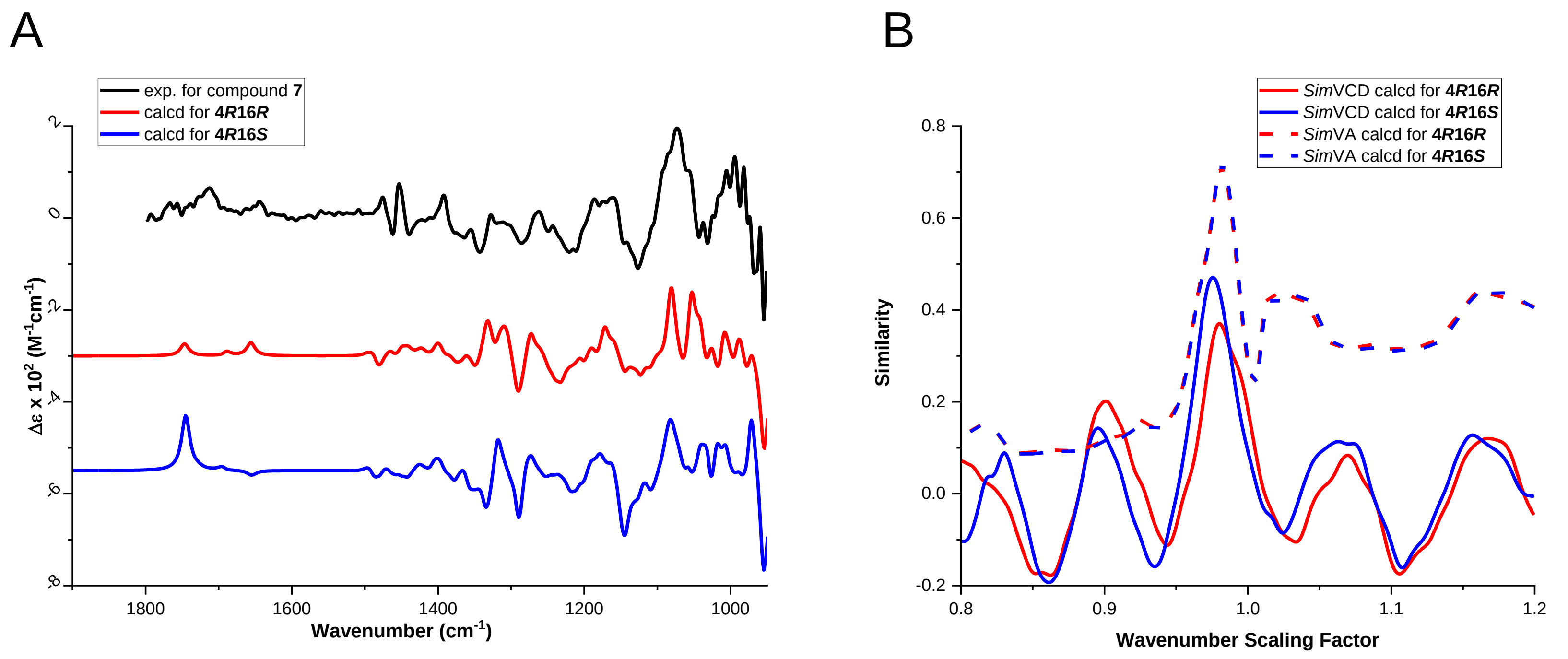

Figure S56. Comparison of experimental and computed VCD spectra in $CDCl_3$ for compound **7**.
(A). Similarities (*Sim*VA and *Sim*VCD) between experimental and computed VA and VCD spectra of **7** were plotted as functions of wavenumber scale factor (B). 4*R* stands for 4*R*,5*R*,6*S*,8*R*,9*R*,10*S*,23*S*. The wavenumber scale factor corresponding to the maximal *Sim*VA value in B (0.9820) was used to scale the computed spectra in A.

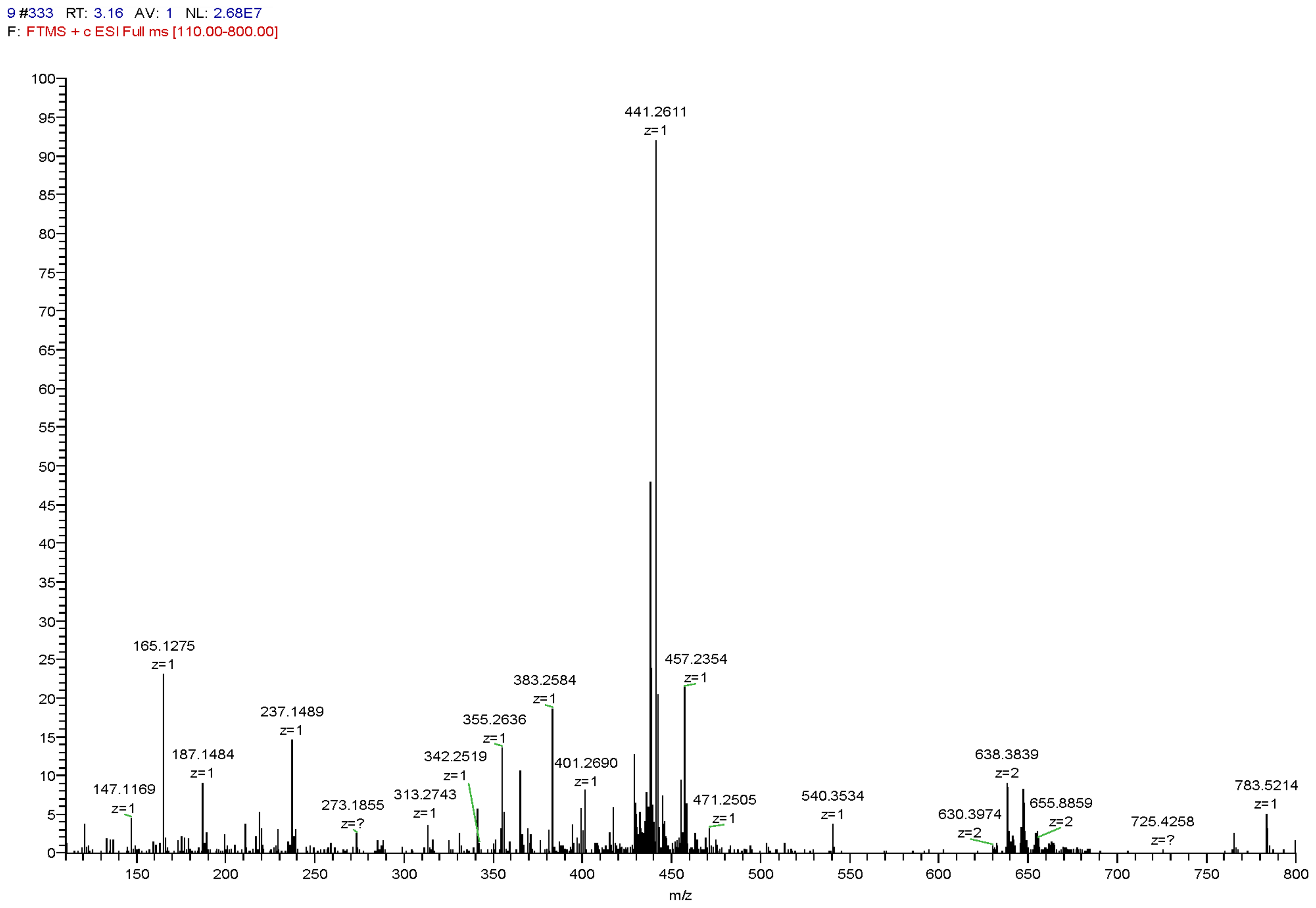

Figure S57. HRESIMS spectrum of compound **7**.

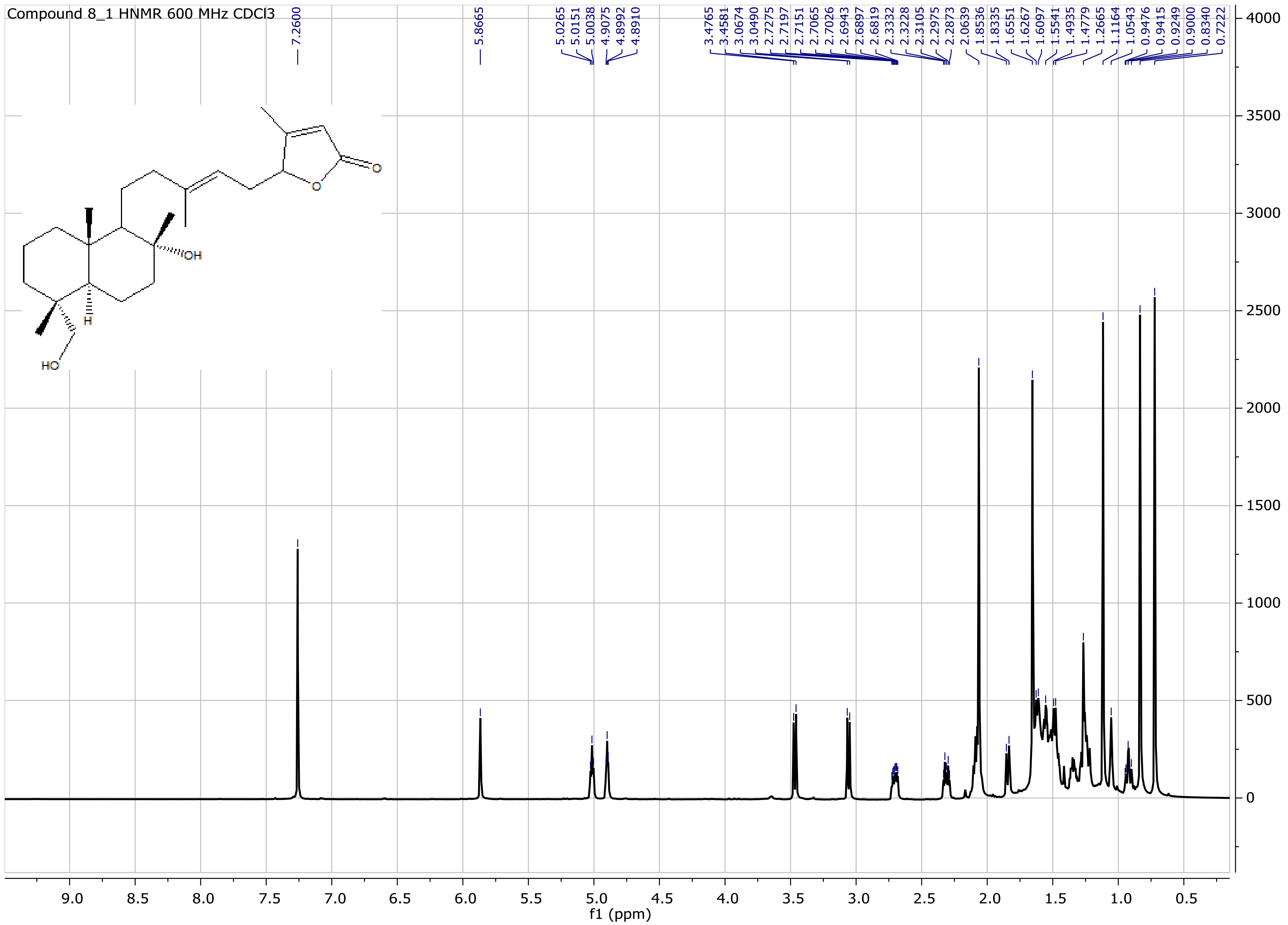

Figure S58. ^{1}H NMR (600 MHz, $CDCl_3$) spectrum of compound **8**.

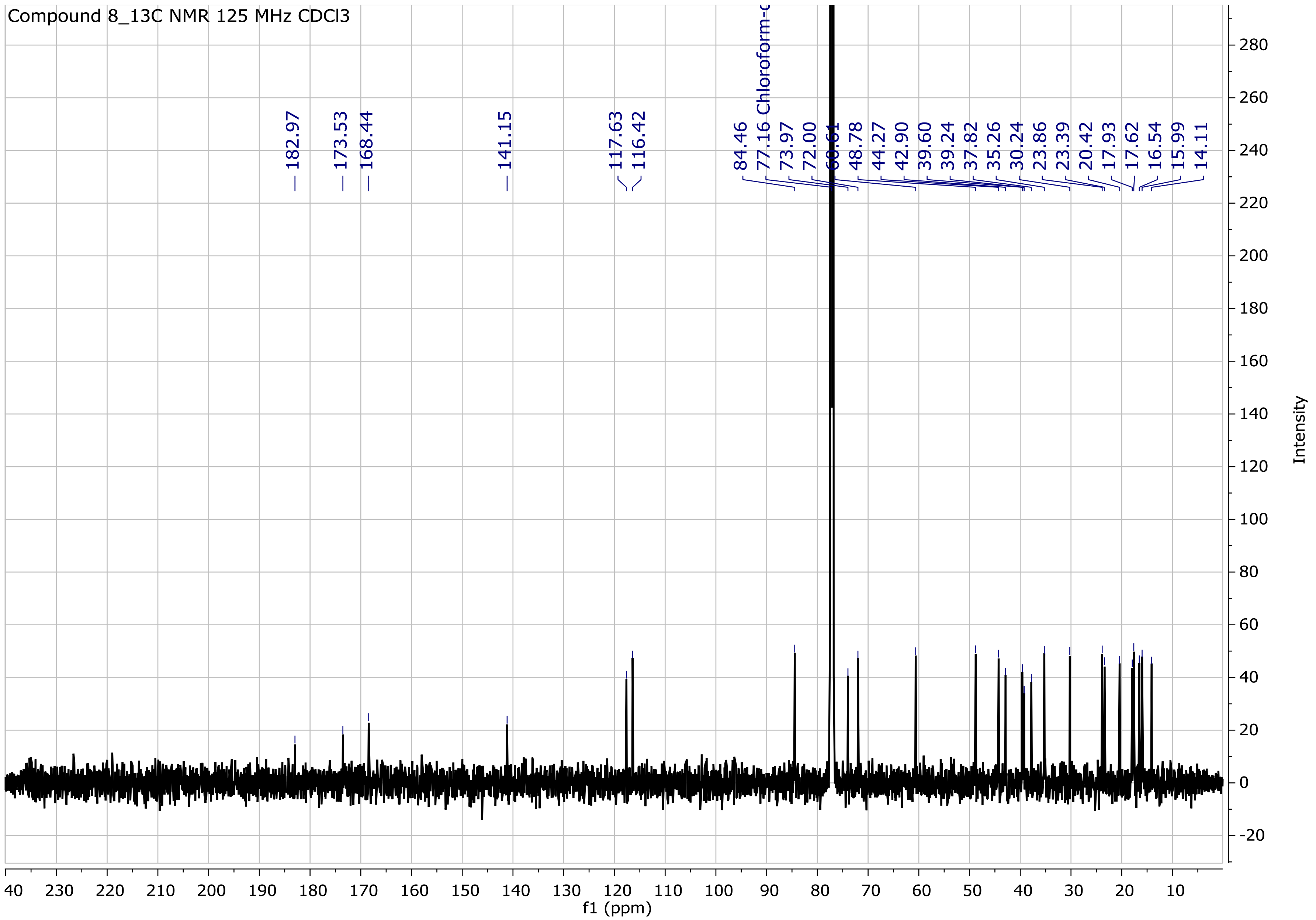

Figure S59. ^{13}C NMR (125 MHz, $CDCl_3$) spectrum of compound **8**.

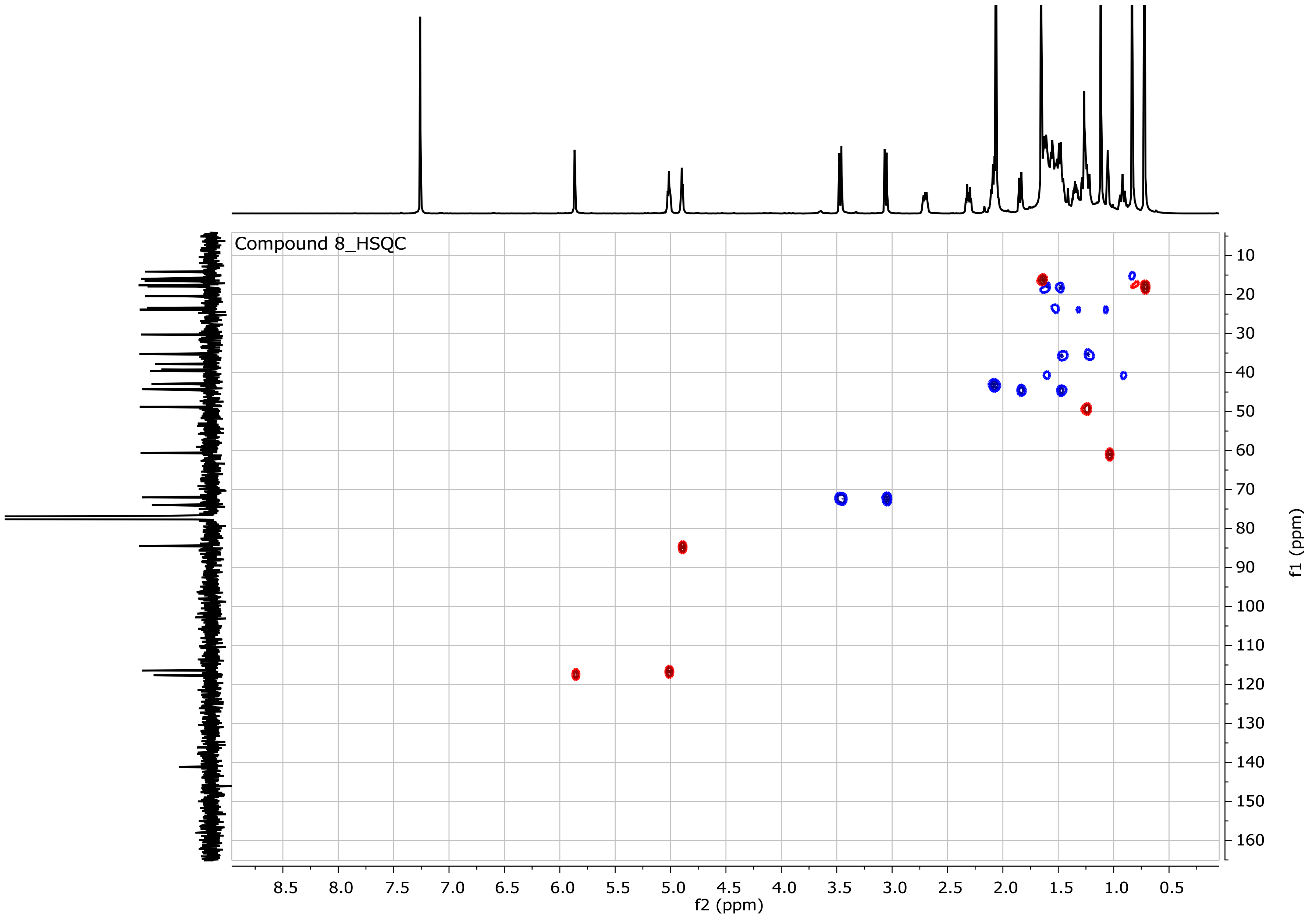

Figure S60. HSQC (600 MHz, $CDCl_3$) spectrum of compound **8**.

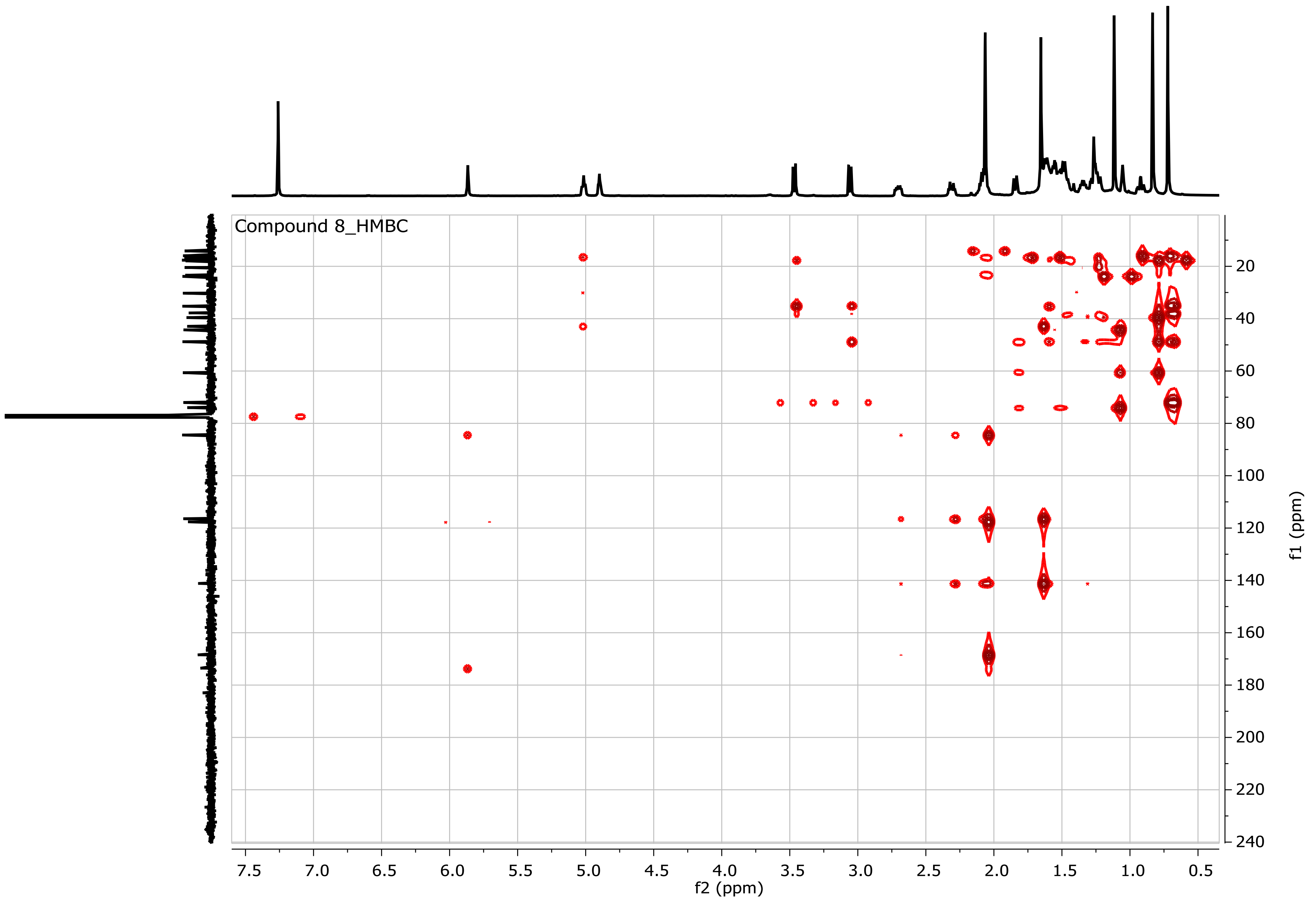

Figure S61. HMBC (600 MHz, $CDCl_3$) spectrum of compound **8**.

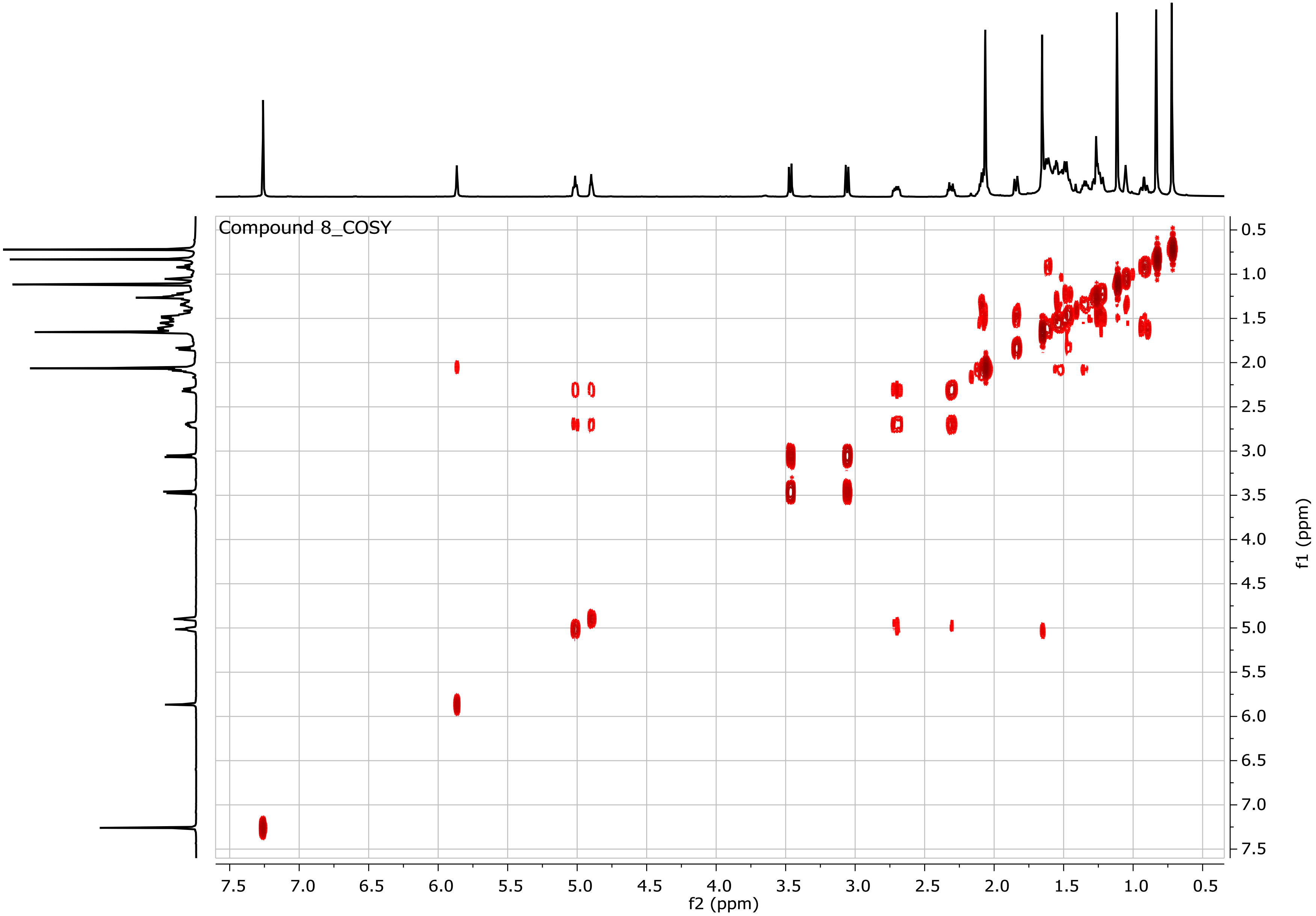

Figure S62. COSY (600 MHz, $CDCl_3$) spectrum of compound **8**.

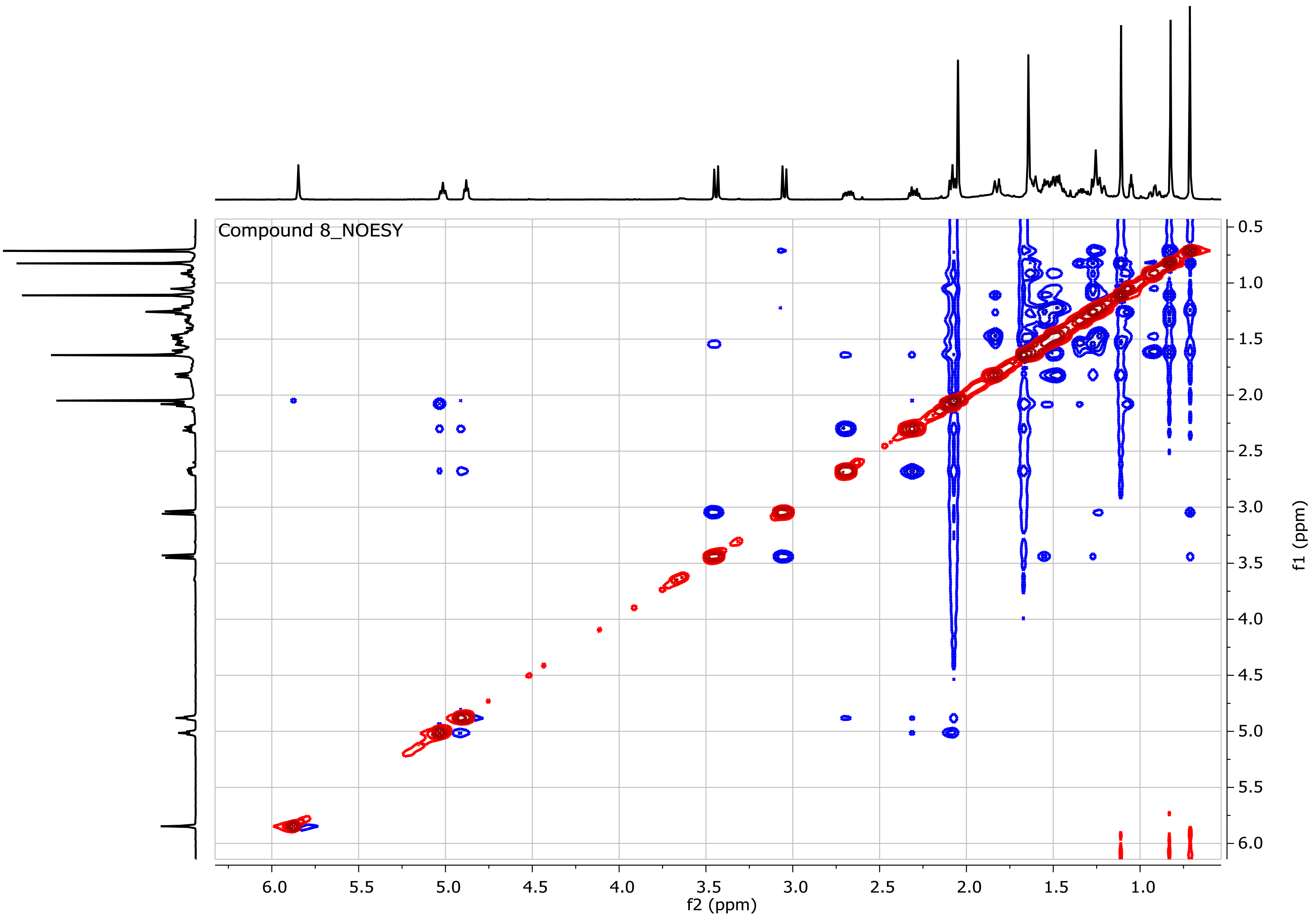

Figure S63. NOESY (500 MHz, $CDCl_3$) spectrum of compound **8**.

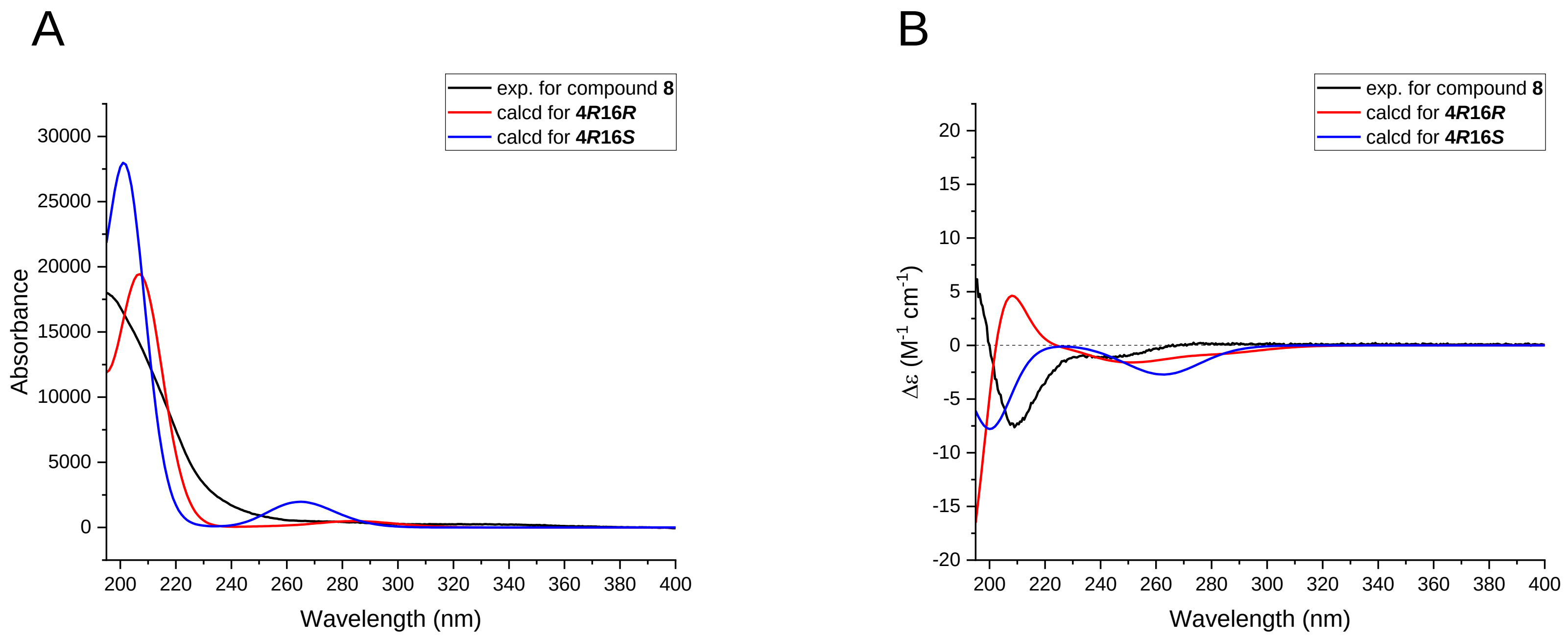

Figure S64. Comparison of experimental and computed UV (A) and ECD (B) (CH_3OH) spectra for compound **8**. 4*R* stands for 4*R*5*R*8*R*9*R*10*S*.

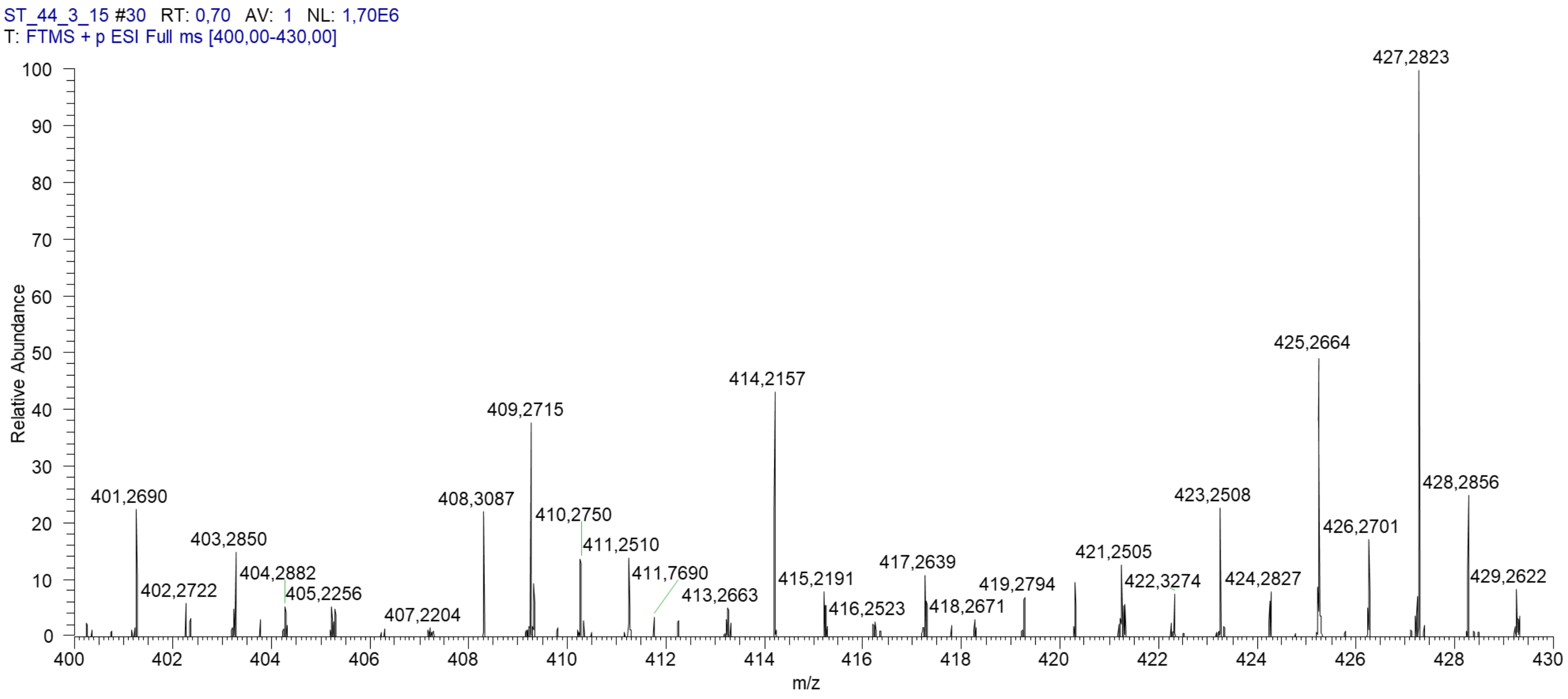

Figure S65. HRESIMS spectrum of compound **8**.

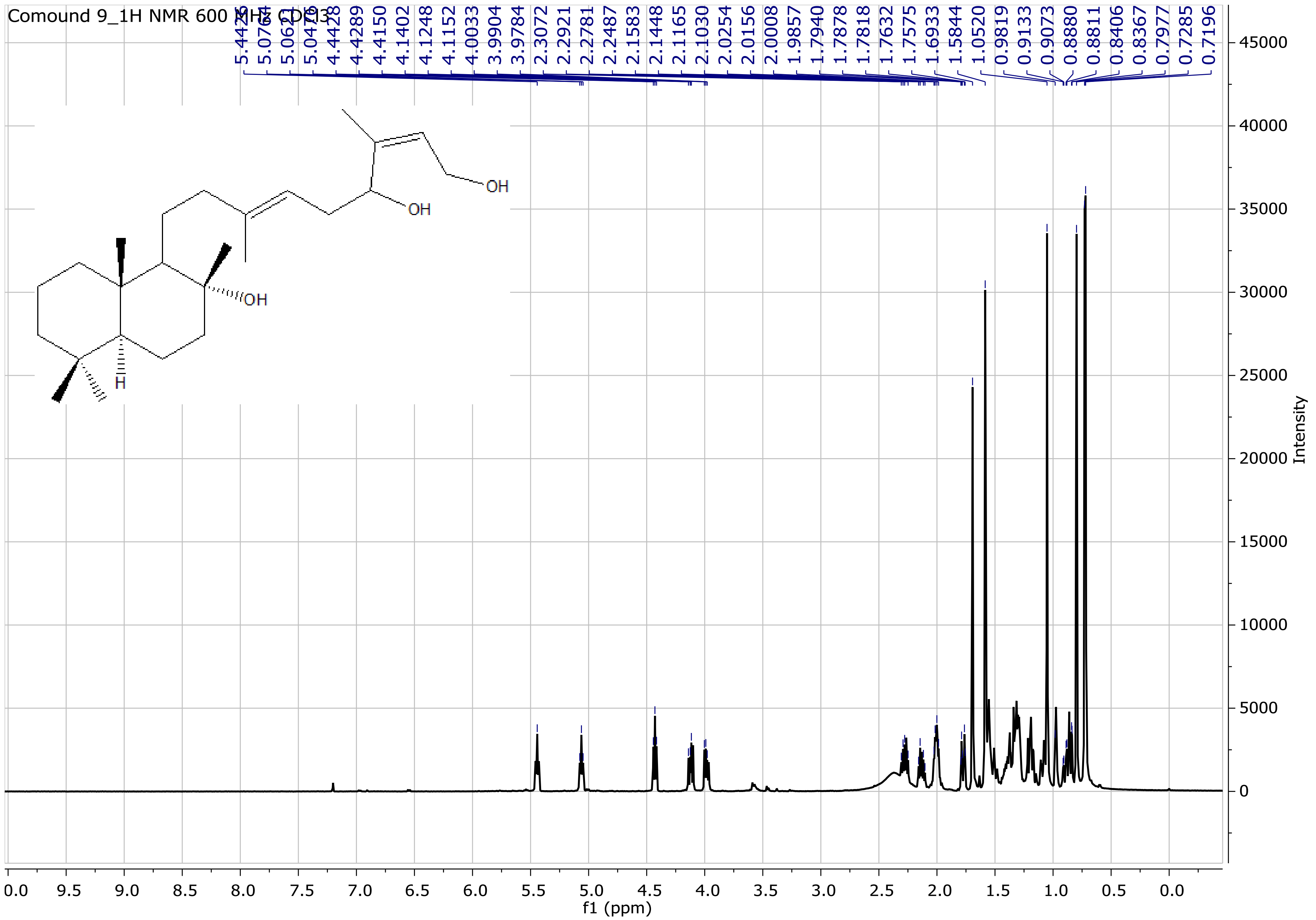

Figure S66. ^{1}H NMR (600 MHz, $CDCl_3$) spectrum of compound **9**.

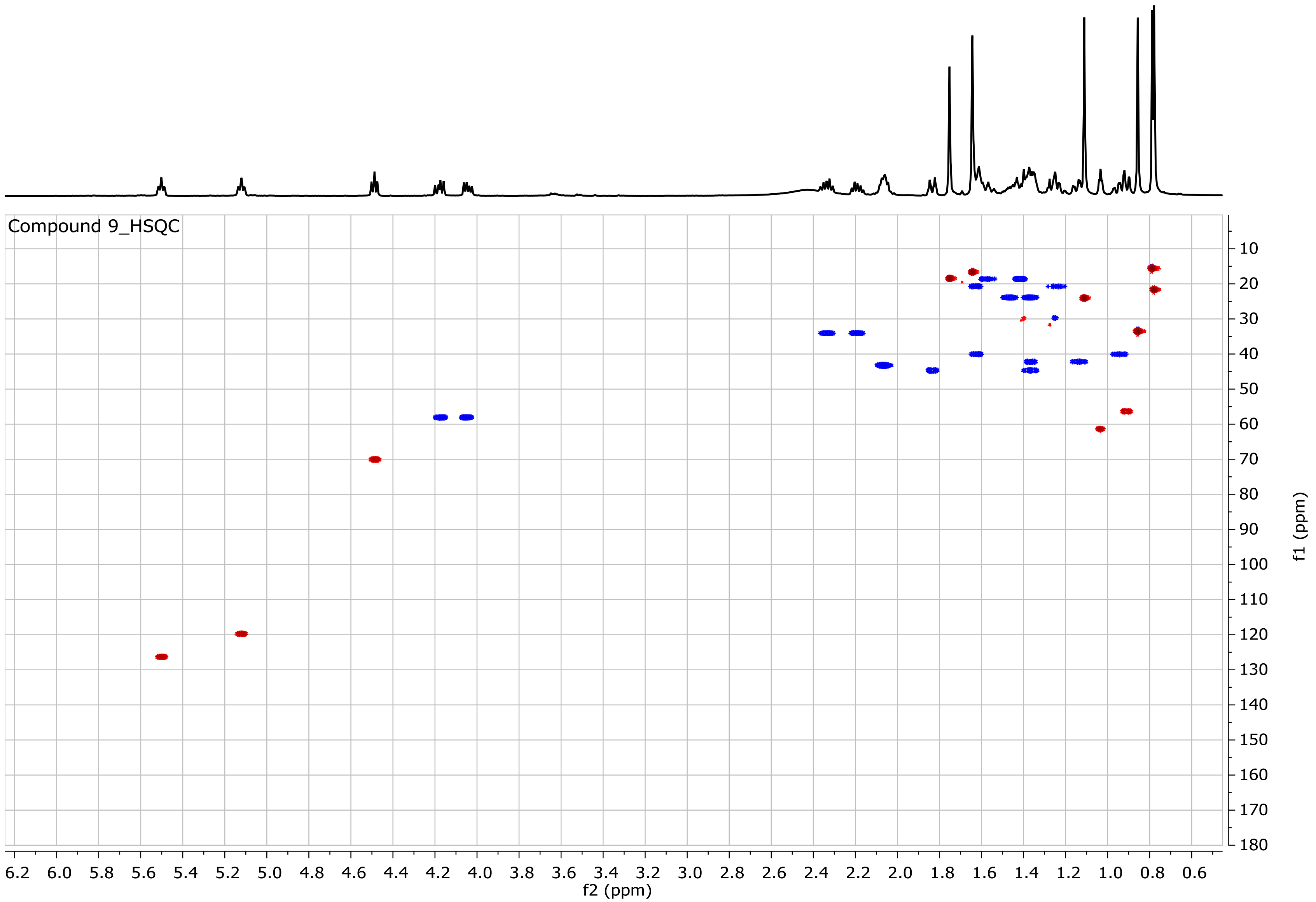

Figure S67. HSQC (600 MHz, $CDCl_3$) spectrum of compound **9**.

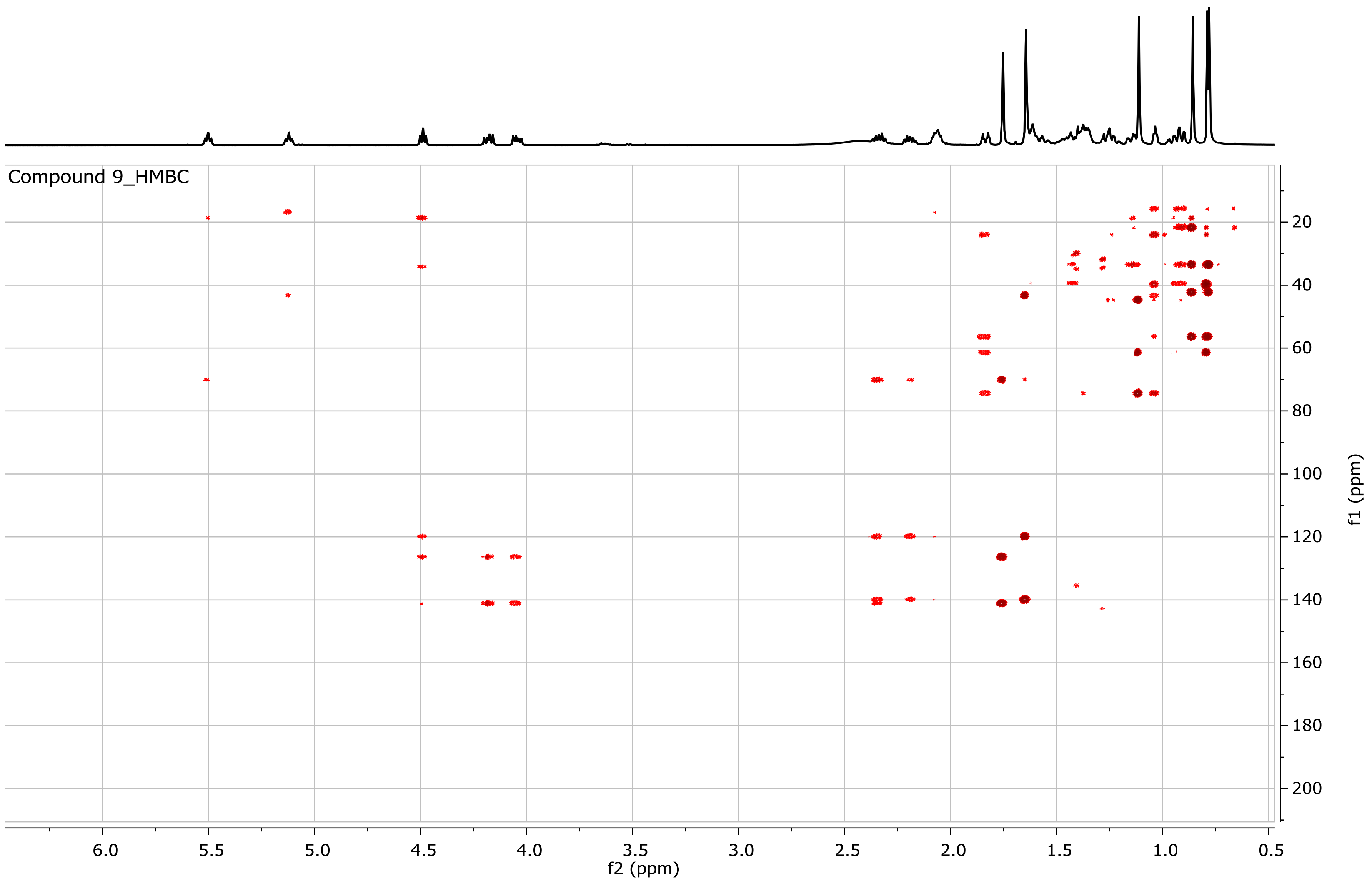

Figure S68. HMBC (600 MHz, $CDCl_3$) spectrum of compound **9**.

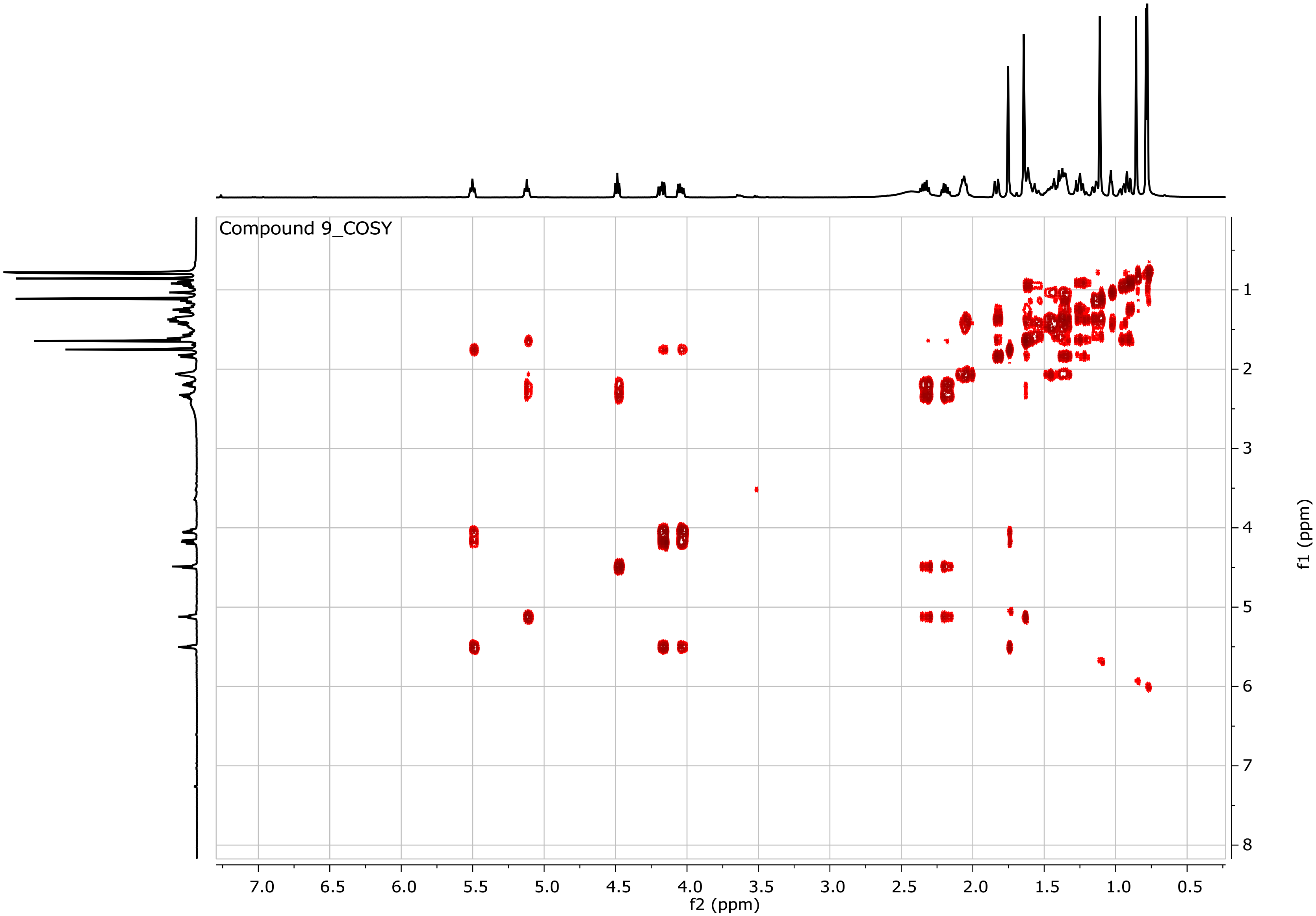

Figure S69. COSY (600 MHz, $CDCl_3$) spectrum of compound **9**.

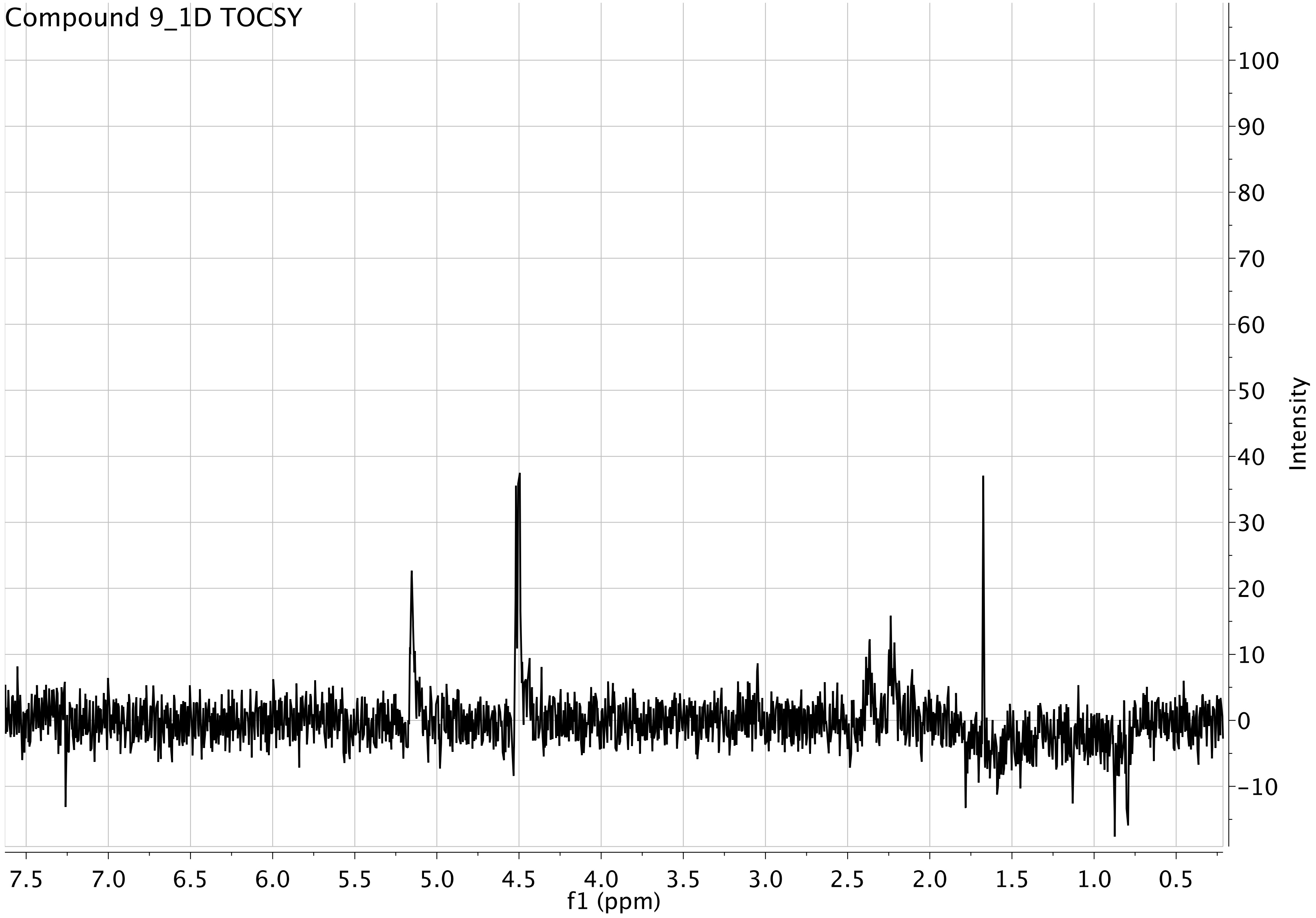

Figure S70. 1D TOCSY (600 MHz, $CDCl_3$) spectrum of compound **9**.

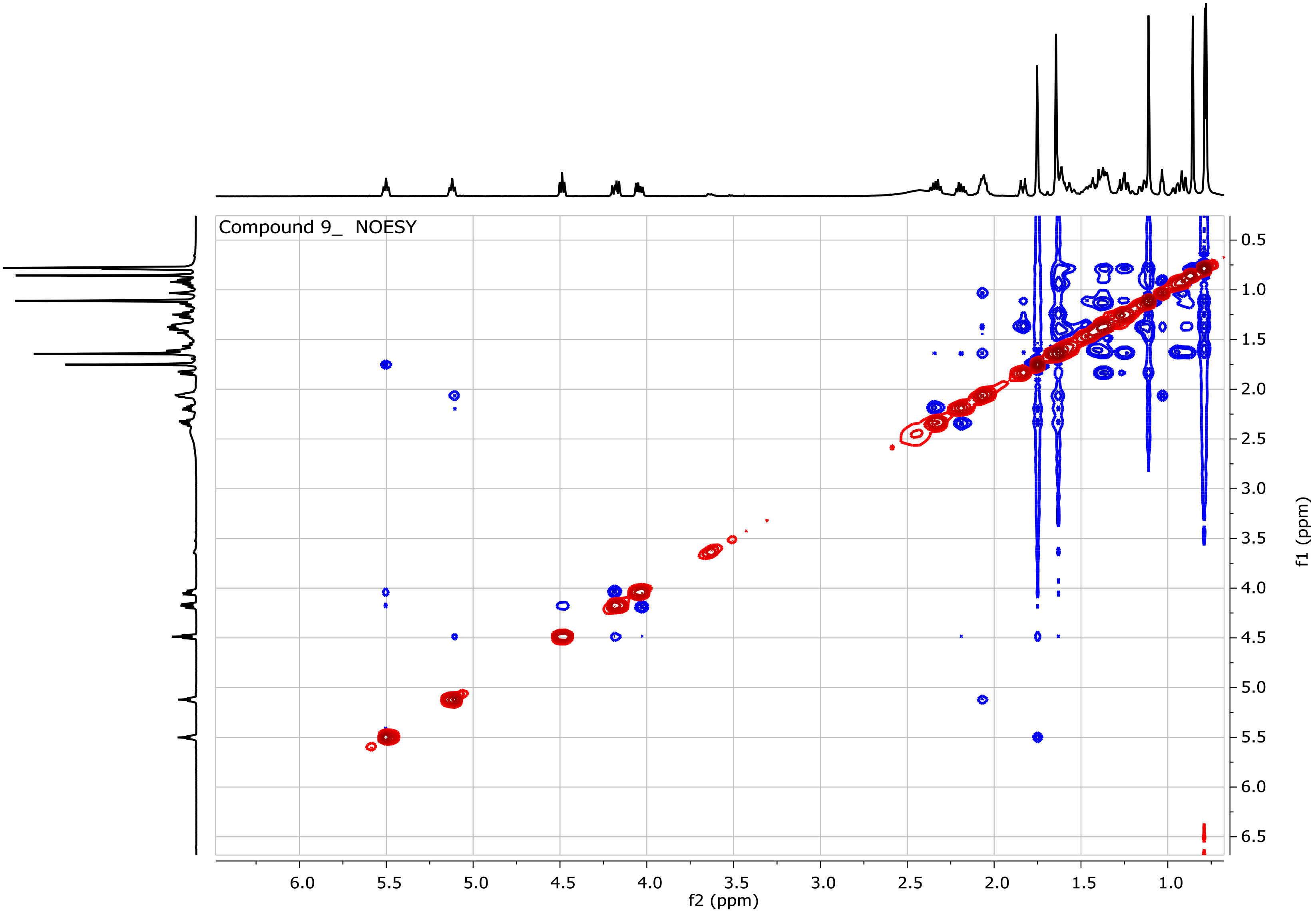

Figure S71. NOESY (500 MHz, $CDCl_3$) spectrum of compound **9**.

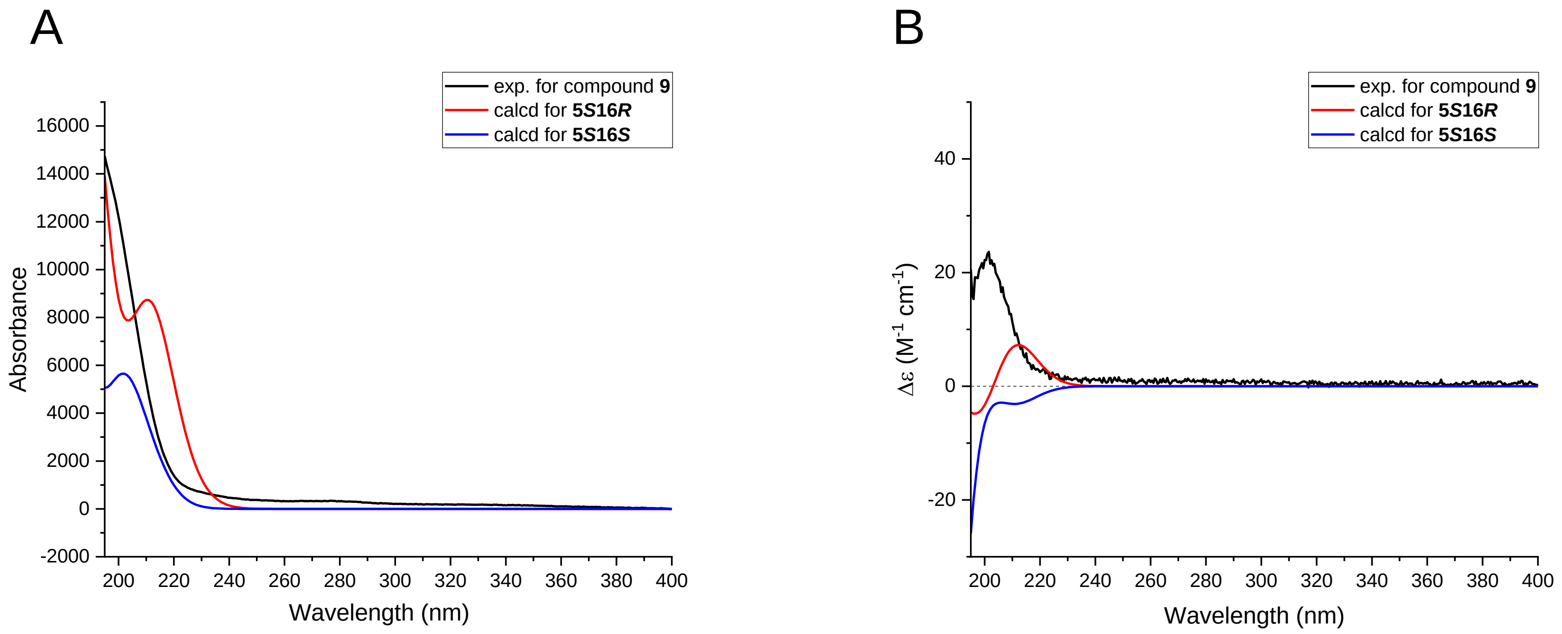

Figure S72. Comparison of experimental and computed UV (A) and ECD (B) spectra (CH_3OH) for compound **9**. 5*S* stands for 5*S*8*R*9*R*10*S*.

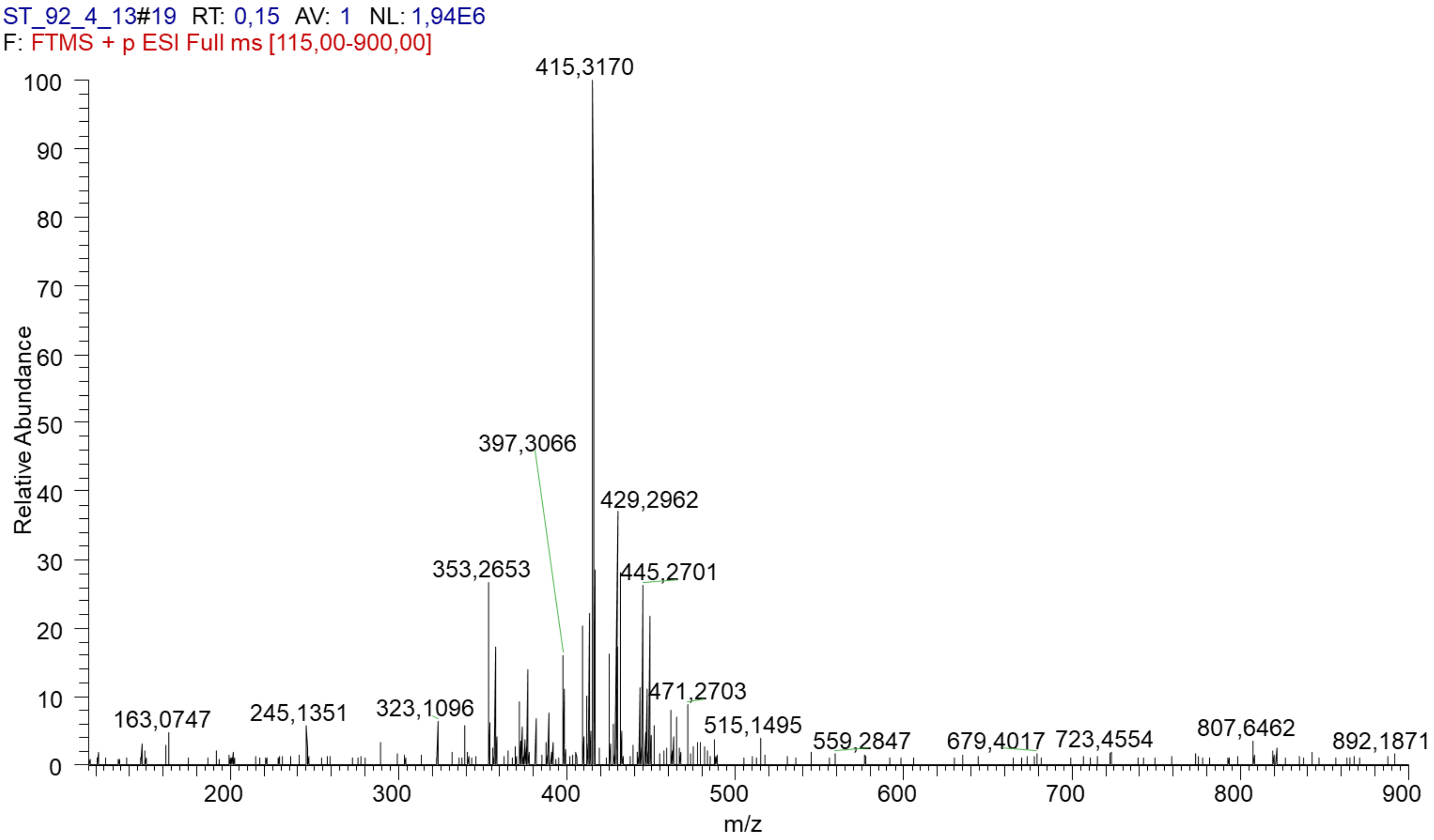

Figure S73. HRESIMS spectrum of compound **9**.

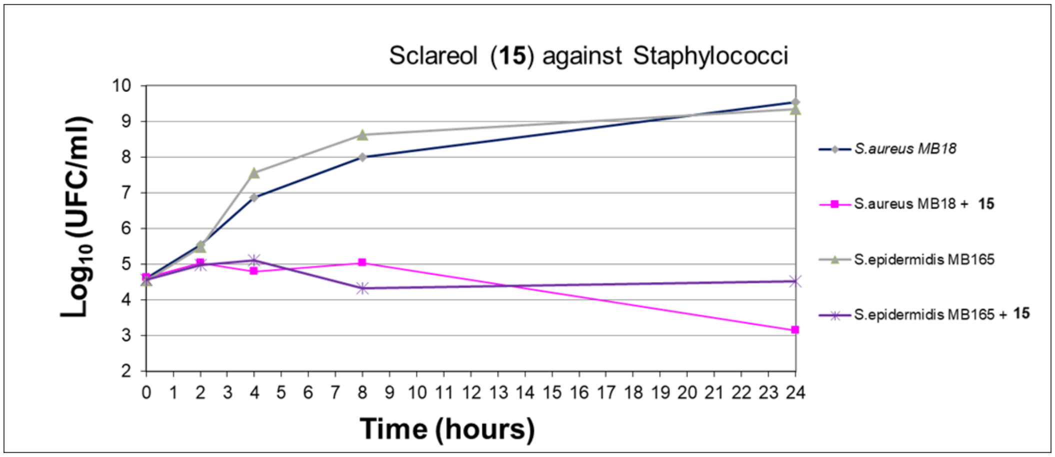

Figure S74. Effect of sclareol (**15**) on viable cell number of selected susceptible *Staphylococcus* strains. Time-kill curves were recorded in the absence or in the presence of 15 at a concentration of 4 × MIC.

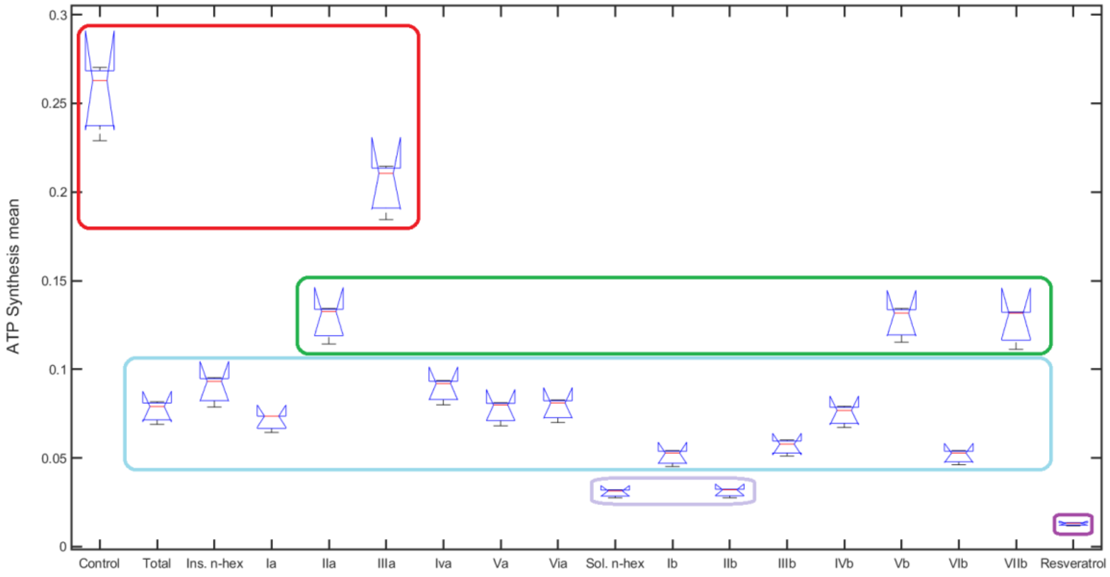

Figure S75. Effect of total extract, *n*-hexane insoluble and soluble fractions and relative main fractions (I_a-VI_a, I_b-VI_b) on ATP synthesis in rod Outer Segments (OS).
ATP synthesis by OS (5µg) in the presence of total extract, n-hexane insoluble and soluble fractions and relative main fractions (I_a-VI_a, I_b-VI_b) (80 µg/mL). Activity is expressed as µmol ATP produced/min/mg of total protein. All data were tested with one-way ANOVA (using MATLAB 2019a statistical toolkit). Mean and standard deviation of each measure are presented according to the standards of MATLAB boxplot. The application of Bonferroni method defined 5 groups ($p < 0.05$) indicated by colored rectangles.

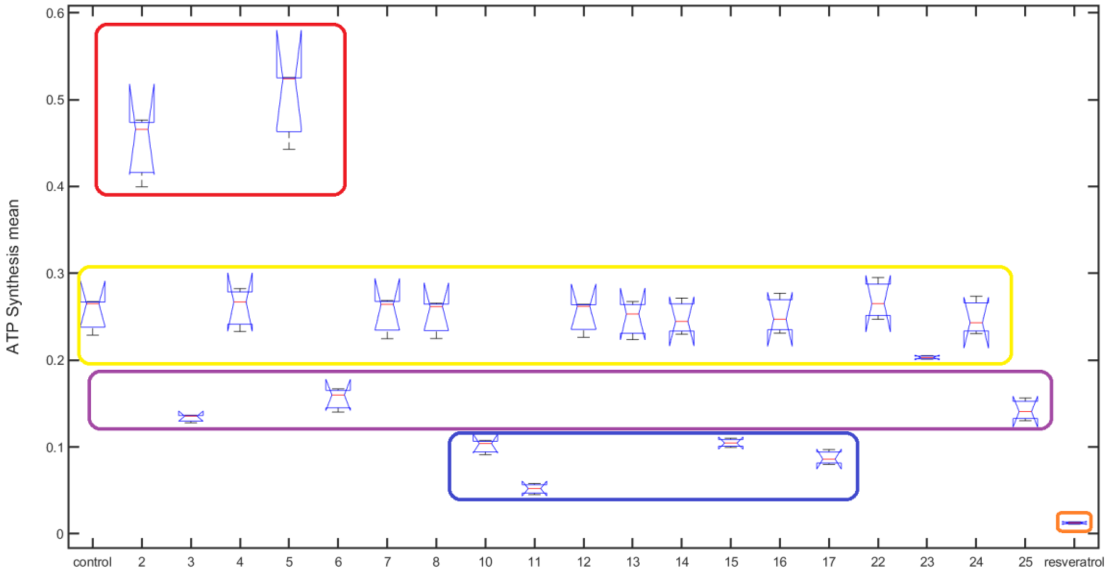

Figure S76. Effect of compounds isolated from *S. tingitana* on ATP synthesis in rod Outer Segments (OS). ATP synthesis by OS (5µg) in the presence of the pure compounds (80 µg/mL). Activity is expressed as µmol ATP produced/min/mg of total protein. All data were tested with one-way ANOVA (using MATLAB 2019a statistical toolkit). Mean and standard deviation of each measure are presented according to the standards of MATLAB boxplot. The application of Bonferroni method defined 5 groups ($p < 0.05$) indicated by colored rectangles.

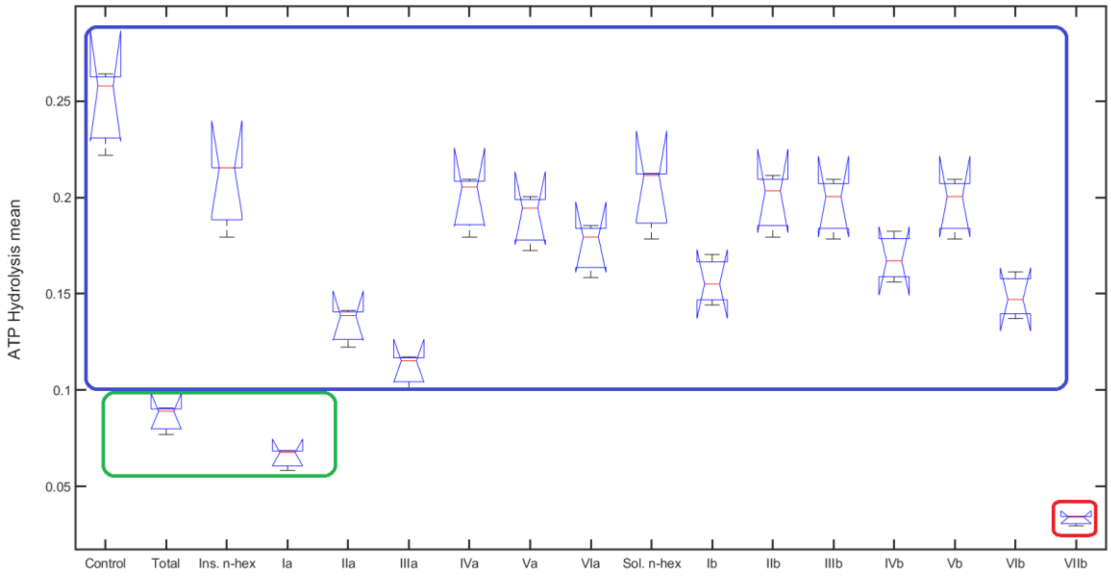

Figure S77. Effect of total extract, *n*-hexane insoluble and soluble fractions and relative main fractions (I_a-VI_a, I_b-VI_b) on ATP hydrolysis activity in rod Outer Segments (OS).
ATP hydrolysis by OS (5 µg) in the presence of total extract, *n*-hexane insoluble and soluble fractions and relative main fractions (I_a-VI_a, I_b-VI_b) (80 µg/mL). Positive control: 30 µM resveratrol. Activity is expressed as µmol ATP hydrolyzed/min/mg of total protein. All data were tested with one-way ANOVA (using MATLAB 2019a statistical toolkit), Mean and standard deviation of each measure is presented according to the standards of MATLAB boxplot. The application of Bonferroni method defined 3 groups ($p < 0.05$) indicated by colored rectangles.

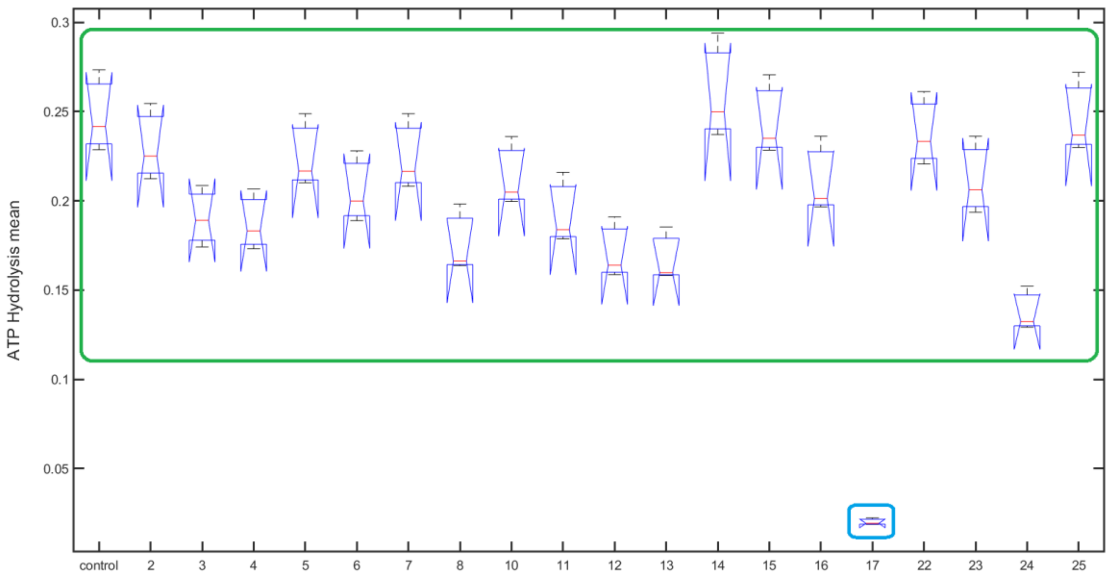

Figure S78. Effect of compounds on isolated from *S. tingitana* on ATP hydrolysis activity in rod Outer Segments (OS).
ATP synthesis by OS (5µg) in the presence of the pure compounds (80 µg/mL). Activity is expressed as µmol ATP produced/min/mg of total protein. All data were tested with one-way ANOVA (using MATLAB 2019a statistical toolkit). Mean and standard deviation of each measure are presented according to the standards of MATLAB boxplot. The application of Bonferroni method defined 2 groups ($p < 0.05$) indicated by colored rectangles. The most active group contains only manool (**17**).

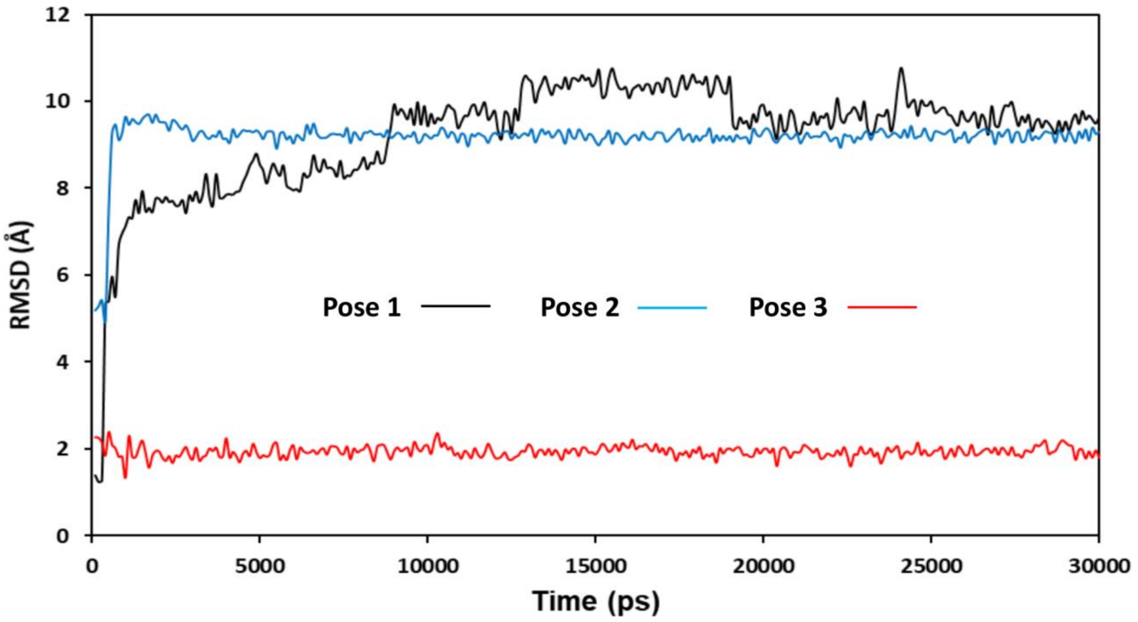

Figure S79. MD analysis of the three binding modes predicted by docking manool (17) into F1-ATPase. The plot shows the RMSD of the ligand disposition during the simulation with respect to its initial docking pose.

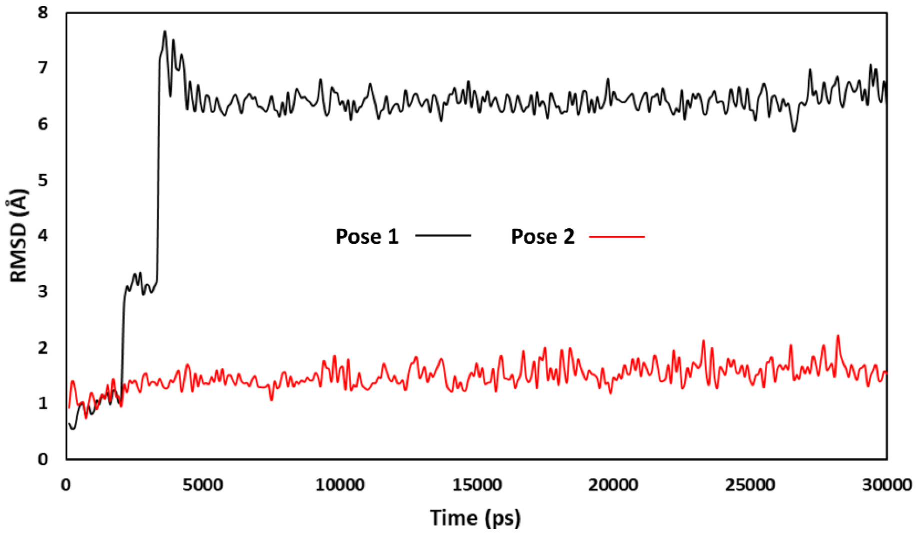

Figure S80. MD analysis of the two binding modes predicted by self-docking quercetin into the co-crystal structure of F1-ATPase.
The plot shows the RMSD of the ligand disposition during the simulation with respect to its initial docking pose.

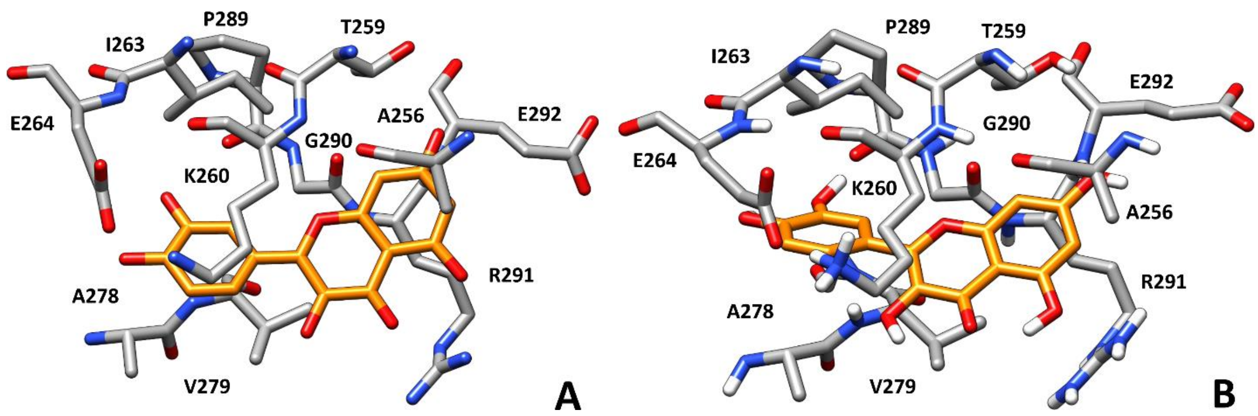

Figure S81. A) X-ray structure of F1-ATPase bound to quercetin (PDB code 2JJ2) and B) minimized average structure of F1-ATPase in complex with quercetin in binding mode 2.
Hydrogen bonds are represented as black dashed lines.

Table S1. MIC values of total extract *n*-hexane insoluble and soluble fractions and relative main fractions against representative clinical strains.

Microbial strains	**Total extract**	***n*-hexane Insol.**	**I_a**	**II_a**	**III_a**	**IV_a**	**V_a**	**VI_a**	***n*-hexane Sol.**	**I_b**	**II_b**	**III_b**	**IV_b**	**V_b**	**VI_b**
E. faecalis MB 1 (VRE)	128	128	>128	>128	>128	>128	>128	>128	>128	>128	128	64	128	>128	>128
E. faecium MB 152 (VRE)	128	>128	>128	>128	>128	>128	>128	>128	128	>128	128	128	64	>128	>128
S. aureus MB 18 (MRSA)	128	128	64	>128	>128	128	128	64	128	>128	>128	128	64	64	>128
S. epidermidis MB 165 (MRSE)	>128	>128	32	64	64	128	64	32	64	>128	>128	128	128	128	>128
S. agalactiae MB 149	>128	>128	>128	>128	>128	>128	>128	>128	>128	>128	>128	>128	>128	>128	>128
S. pneumoniae MB 35	>128	>128	>128	>128	>128	>128	>128	>128	>128	>128	>128	>128	>128	>128	>128
Proteus mirabilis MB 14	>128	>128	>128	>128	>128	>128	>128	>128	>128	>128	>128	>128	>128	>128	>128
E.coli MB123	>128	>128	>128	>128	>128	>128	>128	>128	>128	>128	>128	>128	>128	>128	>128
Moraxella catarrhalis MB 15	>128	>128	>128	>128	>128	>128	>128	>128	>128	>128	>128	>128	>128	>128	>128
Klebsiella pneumoniae MB 11	>128	>128	>128	>128	>128	>128	>128	>128	>128	>128	>128	>128	>128	>128	>128
C. albicans MB796	>128	>128	>128	>128	>128	>128	>128	>128	>128	>128	>128	>128	>128	>128	>128
C. glabrata MB796	>128	>128	>128	>128	>128	>128	>128	>128	>128	>128	>128	>128	>128	>128	>128

MIC values, expressed in μg/mL of total extract, *n*-hexane insoluble and soluble fractions and relative main fractions (I_a-VI_a, I_b-VI_b).
MRSA: Methicillin resistant *S. aureus*; MRSE: Methicillin resistant *S. epidermidis*; VRE: Vancomycin resistant *Enterococcus*.

Table S2. MM-PBSA results for the three different ligand protein complexes of manool (**17**) bound to F_1-ATPase.

	**EEL**	**VDW**	**ENPOLAR**	**EPB**	**ΔPBSA**
**Pose 1**	-5.12	-42.61	-4.16	42.75	-9.13
**Pose 2**	-9.27	-44.18	-4.04	45.37	-12.12
**Pose 3**	-11.42	-46.52	-3.81	40.36	-21.39

ΔPBSA is the sum of the electrostatic (EEL) and van der Waals (VDW), as well as polar (EPB) and non-polar (ENPOLAR) solvation free energy. Data are expressed as kcal/mol.

Table S3. MM-PBSA results for the two different ligand protein complexes of quercetin bound to F_1-ATPase.

**MM-PBSA Method**					
	**EEL**	**VDW**	**ENPOLAR**	**EPB**	**ΔPBSA**
**Pose 1**	-37,21	-35,03	-3,10	52,71	-22,63
**Pose 2**	-18,80	-44,98	-2,94	40,90	-25,82

ΔPBSA is the sum of the electrostatic (EEL) and van der Waals (VDW), as well as polar (EPB) and non-polar (ENPOLAR) solvation free energy. Data are expressed as kcal/mol.
